# Transcriptomic and glycomic analyses highlight pathway-specific glycosylation alterations unique to Alzheimer’s disease

**DOI:** 10.1038/s41598-023-34787-4

**Published:** 2023-05-15

**Authors:** Xinyu Tang, Jennyfer Tena, Jacopo Di Lucente, Izumi Maezawa, Danielle J. Harvey, Lee-Way Jin, Carlito B. Lebrilla, Angela M. Zivkovic

**Affiliations:** 1 Department of Nutrition, University of California, Davis, One Shields Ave, Davis, CA 95616 USA; 2 Department of Chemistry, University of California, Davis, Davis, CA USA; 3 Department of Pathology and Laboratory Medicine, School of Medicine, University of California, Davis, Sacramento, CA USA; 4 UC Davis MIND Institute, Sacramento, CA USA; 5 Division of Biostatistics, Department of Public Health Sciences, School of Medicine, University of California, Davis, Davis, CA USA

**Keywords:** Data mining, Glycobiology, Alzheimer's disease

## Abstract

Glycosylation has been found to be altered in the brains of individuals with Alzheimer’s disease (AD). However, it is unknown which specific glycosylation-related pathways are altered in AD dementia. Using publicly available RNA-seq datasets covering seven brain regions and including 1724 samples, we identified glycosylation-related genes ubiquitously changed in individuals with AD. Several differentially expressed glycosyltransferases found by RNA-seq were confirmed by qPCR in a different set of human medial temporal cortex (MTC) samples (n = 20 AD vs. 20 controls). *N*-glycan-related changes predicted by expression changes in these glycosyltransferases were confirmed by mass spectrometry (MS)-based *N*-glycan analysis in the MTC (n = 9 AD vs. 6 controls). About 80% of glycosylation-related genes were differentially expressed in at least one brain region of AD participants (adjusted p-values < 0.05). Upregulation of MGAT1 and B4GALT1 involved in complex N-linked glycan formation and galactosylation, respectively, were reflected by increased concentrations of corresponding *N*-glycans. Isozyme-specific changes were observed in expression of the polypeptide *N*-acetylgalactosaminyltransferase (GALNT) family and the alpha-*N*-acetylgalactosaminide alpha-2,6-sialyltransferase (ST6GALNAC) family of enzymes. Several glycolipid-specific genes (UGT8, PIGM) were upregulated. The critical transcription factors regulating the expression of N-glycosylation and elongation genes were predicted and found to include STAT1 and HSF5. The miRNA predicted to be involved in regulating N-glycosylation and elongation glycosyltransferases were has-miR-1-3p and has-miR-16-5p, respectively. Our findings provide an overview of glycosylation pathways affected by AD and potential regulators of glycosyltransferase expression that deserve further validation and suggest that glycosylation changes occurring in the brains of AD dementia individuals are highly pathway-specific and unique to AD.

## Introduction

Alzheimer’s disease (AD) is the most common form of dementia in the elderly, characterized by the accumulation of extracellular amyloid-β (Aβ) and intracellular hyperphosphorylated tau, as well as the accumulation of cholesterol-containing lipid droplets^1,2^. However, treatments to reduce Aβ load have failed to stop AD progression^3,4^, highlighting the need to discover new targets for preventing and treating AD.

The search for new therapeutic targets for AD has turned to glycobiology because of recent evidence showing major alterations in N- and O-glycosylation in the brain, serum, and cerebrospinal fluid (CSF) of AD patients^5–9^. Glycosylation affects biological processes across the major brain cell types. For example, in neurons, the number of glycosylation sites on neuronal and synaptic proteins is altered in AD brains, leading to aberrant neuronal adhesion and synaptic transmission^10^. Similarly, dysregulated N-glycosylation and sialylation patterns in astrocytes and microglia affect extracellular functions and inflammatory responses^10,11^.

Over 200 glycosyltransferases are involved in glycan biosynthesis, including sulfotransferases. Mutations in these enzymes cause Congenital Disorders of Glycosylation (CDG), and over 80% of CDGs are associated with neurological abnormalities^12^. For example, defects in alpha-1,3-mannosyl-glycoprotein 2-beta-*N*-acetylglucosaminyltransferase (MGAT1) are associated with neurologic defects concurrent with caspase3 activation and neuronal apoptosis^13^. Moreover, the specificity and non-redundant functions of isoenzyme families are still poorly understood. For example, dysfunction in the polypeptide *N*-acetylgalactosaminyltransferase 2 (GALNT2) isoenzyme, which belongs to the GALNT family of glycosyltransferases that initiate mucin-type O-glycosylation, reduced Aβ production by reducing O-GalNAc formation of the amyloid precursor protein (APP)^14^. In contrast, GALNT6 enzymatic activity caused reduced Aβ40 and Aβ42 generation with excess GalNAc-type O-glycosylation of APP^15^. Although several studies have explored the brain glycoproteome in AD^10,16^, it is not clear which glycosylation pathways are specifically affected in AD. Furthermore, the expression profiles of glycosylation genes in different brain regions remain uncharacterized.

In this study, the aim was to conduct an exploratory analysis using publicly available RNA-seq data to provide an overview of glycosylation-related pathways altered in AD across multiple brain regions, and to determine whether some of the predicted alterations could be confirmed by qPCR and *N*-glycomic analysis in one of these brain regions. We performed differential expression analysis of glycosylation genes in AD participants compared to controls and participants with other neurological conditions using a harmonized data set including RNA-seq data from the Mayo, Mount Sinai Brain Bank (MSBB), and Religious Orders Study/Memory and Aging Project (ROSMAP) studies. The RNA-seq data include seven brain regions: the dorsolateral prefrontal cortex (DLPFC), frontal pole (FP), inferior frontal cortex (IFG), superior temporal cortex (STG), parahippocampal gyrus (PHG), temporal cortex (TCX), and cerebellum (CER). Furthermore, we explored whether a subset of the differentially expressed genes discovered in the RNA-seq analysis also showed altered expression by qPCR analysis in a separate set of samples from AD participants vs. controls. We explored whether some of the gene expression differences observed by RNA-seq and qPCR are also observed by mass spectrometry-based *N*-glycan analysis of a separate set of samples from AD participants vs. controls. Finally, potential regulators of glycosyltransferase gene expression were inferred using curated databases and computational tools.

## Materials and methods

### Public RNA-seq data

RNA-seq data were obtained from the RNA-seq Harmonization Study, which harmonized RNA-seq data from the MSBB, Mayo, and ROSMAP studies by re-processing the reads with a uniform pipeline (The sample information is summarized in Supplementary Tables S1, S2 and the Supplementary Methods section in supporting information).

### RNA-seq data normalization and differential expression analysis

Raw gene count data were filtered and normalized with the trimmed mean of M-values (TMM) method with the edgeR package^17^ in R version 4.1.0 (R Foundation for Statistical Computing, Vienna, Austria). The filter step retains genes that have count-per-million (CPM) above 10 in 70% of samples. Only protein-coding genes were used for analysis. A negative binomial model with the quasi-likelihood (QL) F-test was applied to perform the differential expression analysis among diagnosis groups. The model for MSBB and ROSMAP was adjusted for sex, age, RNA Integrity Number (RIN), post-mortem interval (PMI), and batch. Since 34–35% of PMI information and all batch information were unavailable in Mayo study, the model for Mayo did not include these two covariates. ANOVA-like test and pairwise comparisons were performed as needed. p-values were adjusted for multiple hypothesis testing using Benjamini and Hochberg (BH) with a threshold of adjusted p-value ≤ 0.05 (Supplementary Methods).

### Glycosylation-related gene selection

A list of 219 glycosylation-related genes was obtained from a previous publication^18^.

### Human brain tissue collection

Human postmortem brain samples were drawn from the biorepository of the UC Davis Alzheimer’s Disease Research Center (ADRC). Written informed consent, including consent for autopsy, was obtained from study participants or, for those with substantial cognitive impairment, a caregiver, legal guardian or other proxy. Study protocols were reviewed and approved by the Institutional Review Board (IRB). For postmortem diagnosis, we followed the National Institute on Aging-Alzheimer’s Association guideline for the neuropathologic assessment of AD^19^. The samples for qPCR were from the medial temporal cortex (MTC). The specimens for the *N*-glycomics were from the MTC, lateral prefrontal cortex (LPFC), and lateral cerebellar cortex (LCBC). The brain tissue used for the current study was snap frozen during autopsy and was stored at − 80 °C before RNA extraction (see Supplementary Methods).

### Quantitative real-time PCR

DEGs identified by RNA-sequencing were selected for confirmation by qPCR based on the following criteria: (1) differentially expressed in multiple brain regions, (2) high fold-change in one or more brain regions, and (3) involved in *N*-glycan, *O*-GalcNAc-glycan, glycolipid biosynthesis or elongation, branching, or capping process. Total RNA from brain tissue samples (n = 20 AD vs. n = 20 controls) was extracted using RNeasy^®^ Plus Universal Mini Kit (Qiagen, Valencia, CA) according to the manufacturer’s protocol. The primer sequences used are listed in Supplementary Table S3. Gene expression was normalized to an endogenous reference gene, β-actin. Data were analyzed by the 2^−ΔΔCt^ method. All experiments were performed in duplicate (see Supplementary Methods).

### Cell membrane extraction for *N*-glycan analysis

A total of 51 tissue samples from 20 participants (n = 10 AD vs. n = 10 controls) (Supplementary Table S4) were analyzed. Tissue samples were homogenized, resuspended and lysed. Lysates were pelleted by centrifugation at 2000×*g* for 10 min to remove the nuclear fraction and cell debris. The supernatant was centrifuged at 200,000×*g*, 4 °C for 45 min in series to remove other nonmembrane subcellular fractions (see Supplementary Methods).

### Release and purification of *N*-glycans

Proteins were suspended and denatured. Glycans were released using 2 µL (500,000 units/mL) of peptide *N*-glycosidase F (glycerol-free; New England BioLabs, cat. no. P0705L). Ultracentrifugation at 200,000×*g* was used to separate the *N*-glycans and the membrane fraction. The released *N*-glycans were purified by solid-phase extraction. *N*-Glycans were eluted, dried and reconstituted in 30 µL of water before mass spectrometric analysis (see Supplementary Methods).

### Glycomic analysis by LC–MS/MS

Purified brain *N*-glycans were analyzed using an Agilent nano-LC/chip Q-ToF MS system. Relative abundances were determined by integrating peak areas for observed glycan masses, averaging abundances from instrumental triplicates and normalizing to the summed peak areas of all glycans detected (see Supplementary Methods).

### Transcription factors and miRNA-target interaction inference

Transcription factors (TF) and their binding motifs of glycosyltransferases were identified using the RcisTarget R package^20^. We used the motif collection based on human hg38 genome assembly and RefSeq genes and searched 10 kbp around the transcription start site (TSS). MicroRNAs and their interactions with glycosyltransferases were inferred based on three databases with experimentally validated miRNA-target interactions: miRecords, miRTarBase, and TarBase. The information was retrieved using the multiMiR package^21^. (see Supplementary Methods).

### Statistical analysis

For normally distributed data, groups were compared using Student’s t-test. The fractions of subtypes, sialylated and fucosylated *N*-glycans were calculated as the sum of relative abundances of single glycans. Since we obtained more than one brain tissue from the same participant, differential abundance analysis was performed as linear mixed models using the lmerTest package in R^22^ as shown here: glycan abundance ~ diagnosis × region + (1|subject_id), where subject-specific effects were modeled as random effects. Contrasts between AD and control diagnosis were evaluated for each brain region. These contrasts produced t-statistics and corresponding p-values.

### Ethics approval and consent to participate

Study protocols were reviewed and approved by the Institutional Review Board (IRB). Written informed consent, including consent for autopsy, was obtained from study participants or, for those with substantial cognitive impairment, a caregiver, legal guardian or other proxy.

## Results

### Glycosyltransferase profiling of human AD brain specimens

Human postmortem brain RNA-seq data covered seven distinct regions: the DLPFC, FP, IFG, STG, PHG, TCX, and CER. To identify the AD-associated transcriptome, we performed differential expression analysis on all protein-coding genes in each region independently, comparing AD participants to controls. Genes that did not meet the filtering criteria were excluded from differential expression analysis and included 13–17 genes in each brain region, representing approximately 5% of glycosylation-related genes in each region (Supplementary Table S5). The number of DEGs in each brain region varied with approximately 13,822 protein-coding genes differentially expressed in at least one brain region (adjusted p-value < 0.05, Supplementary Fig. S1A). Glycosylation-related genes represented 0.9–1.3% of total DEGs, and about 80% of glycosylation-related genes were differentially expressed in at least one brain region. The DLPFC and FP had fewer differentially expressed glycosyltransferases than other brain regions (Supplementary Fig. S1B). Two upregulated genes, including alpha-*N*-acetylgalactosaminide alpha-2,6-saialyltransferase 2 (ST6GALNAC2), and three downregulated genes, including ST6GALNAC5 were observed in all brain regions (Supplementary Fig. S1C). Genes encoding enzymes for O-glycosylation and sialylation had stronger signals than N-glycosylation and fucosylation in multiple brain regions of AD participants compared to controls (Supplementary Fig. S1D).

### Gene expression of MGAT1 was increased, with corresponding increases in complex N-glycans

**Figure 1:**
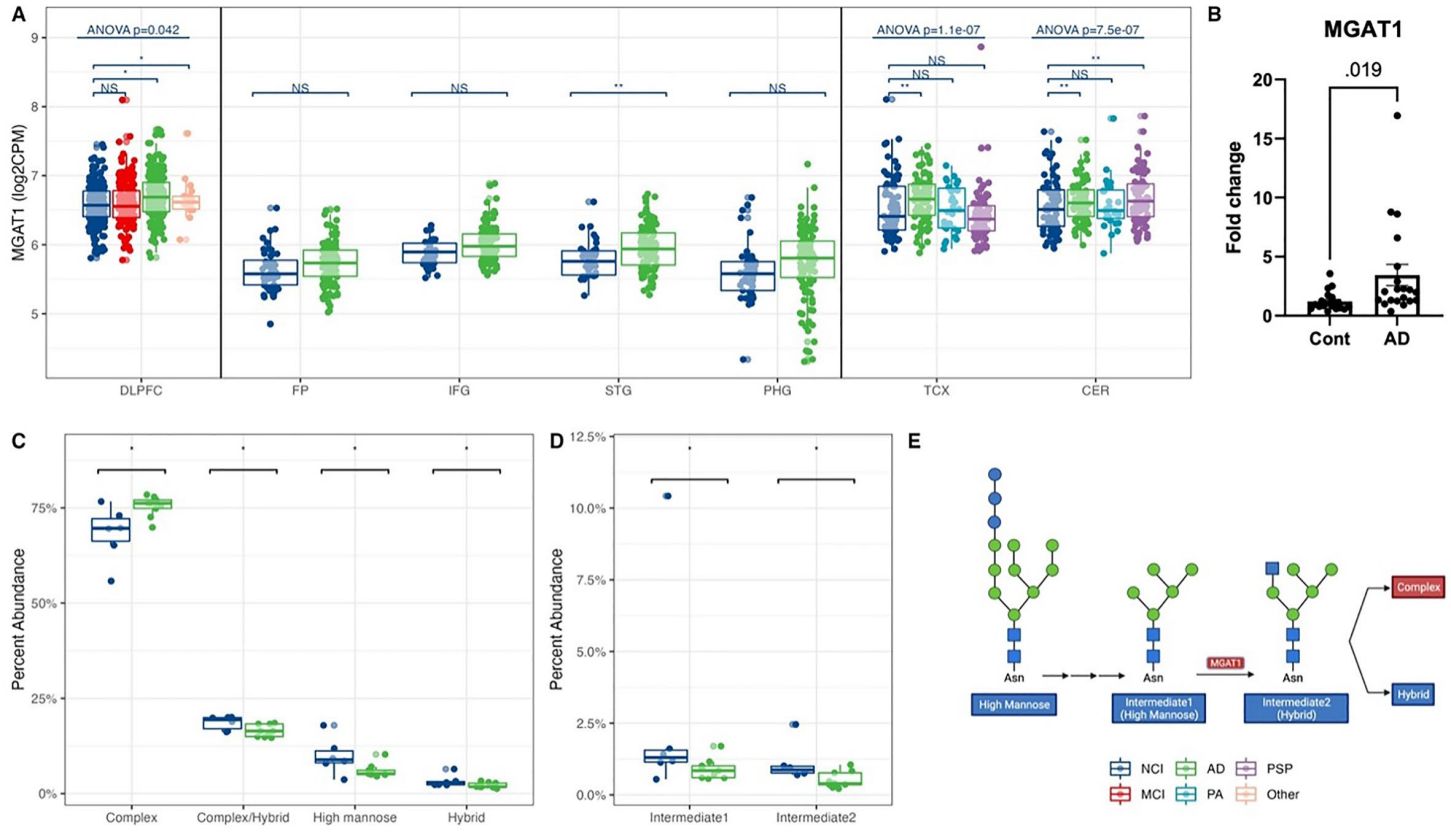
Increased MGAT1 expression corroborated by a raised complex and reduced high-mannose N-glycans abundance in AD. (**A**) MGAT1 gene expression from RNA-seq across human brains with different diagnoses. Data presented as log2 count per million (log2CPM). Data from different studies were separated into blocks. Adjusted p-values were calculated based on all protein-coding genes. NS p-value > 0.05, *p-value < 0.05 but adjusted p-value > 0.05, **p-value < 0.05 and adjusted p-value < 0.05. ANOVA p-values were after the multiple testing correction over all protein-coding genes. *DLPFC* dorsolateral prefrontal cortex, *FP* frontal pole, *IFG* inferior frontal gyrus, *STG* superior temporal gyrus, *PHG* parahippocampal gyrus, *TCX* temporal cortex, *CER* cerebellum. *NCI*: no cognitive impairment, *MCI* mild cognitive impairment, *AD* Alzheimer’s disease, *PA* pathological aging, *PSP* progressive supranuclear palsy, *Other* other types of dementia. (**B**) qPCR confirmation of MGAT1 expression in the medial temporal cortex (MTC) of AD and control participants. (**C**) The abundance of *N*-glycan subtypes from *N*-glycomics in MTC of AD and control participants. (**D**) The abundance of two intermediate structures (Hex:5 HexNAc: 2, Hex:5 HexNAc: 3) from *N*-glycomics in MTC of AD and control participants. *p-value < 0.05. (**E**) A schematic of the MGAT1 function. Red/blue boxes: higher/lower in AD than control. Glycan symbol key: green circles, mannose (Man); blue squares, *N*-acetylglucosamine (GlcNAc).

We first analyzed the expression of genes involved in N-glycosylation. We detected 27 genes encoding glycosyltransferases for *N*-glycan synthesis in at least one brain region (Supplementary Fig. S2A). Among these glycosyltransferases, MGAT1, essential for converting high-mannose structures to hybrid and complex glycans, had increased expression in the brains of AD participants compared to controls in the STG, TCX and CER, with the largest increase across studies in the TCX (Fig. 1A, Supplementary Fig. S2A). The increased gene expression of MGAT1 determined by RNA-seq was also observed by qPCR in an independent set of samples, showing approximately a fourfold increase in gene expression in AD participants vs. healthy controls (Fig. 1B).

We further sought to determine whether the increase in MGAT1 gene expression could also be observed as changes in the *N*-glycome in an additional independent set of brain samples. The *N*-glycomic analysis identified 429 *N*-glycans in the LPFC, MTC, and LCBC, with complex *N*-glycans being the most abundant structures in the human brain samples. The results showed that the percent abundance of complex *N*-glycans significantly increased in AD participants compared to controls in the MTC, and high-mannose *N*-glycans significantly decreased. The observed increases in the final product of the pathway (i.e. complex *N*-glycans), and decreases in the initial precursor of the pathway (i.e. high-mannose *N*-glycans) are consistent with an increased flux through this pathway, of which the expression of the MGAT1 enzyme was observed to be increased by RNA-seq and qPCR (Fig. 1C). The percent abundances of two intermediates of the pathway, the direct substrate for the MGAT1 enzyme, Hex:5 HexNAc:2 (Intermediate 1 in Fig. 1D,E), and the direct product of the MGAT1 enzyme, Hex:5 HexNAc:3 (Intermediate 2 in Fig. 1D,E), were both decreased in AD participants compared with controls. These data suggest a more complex regulation of flux through the entire pathway than a simple decrease in the immediate precursor and increase in the immediate product that would have been predicted by the increased expression of MGAT1 alone. Together these findings support an upregulation of complex *N*-glycan synthesis in the MTC of AD participants. On the other hand, although we found increased MGAT1 expression in the CER, no glycan subtype nor intermediate glycan was significantly changed in the LCBC in AD vs. controls (Supplementary Fig. S2B–D).

### Increased expression of B4GALT1 was accompanied by increased galactosylation of *N*-glycans in AD brains

**Figure 2:**
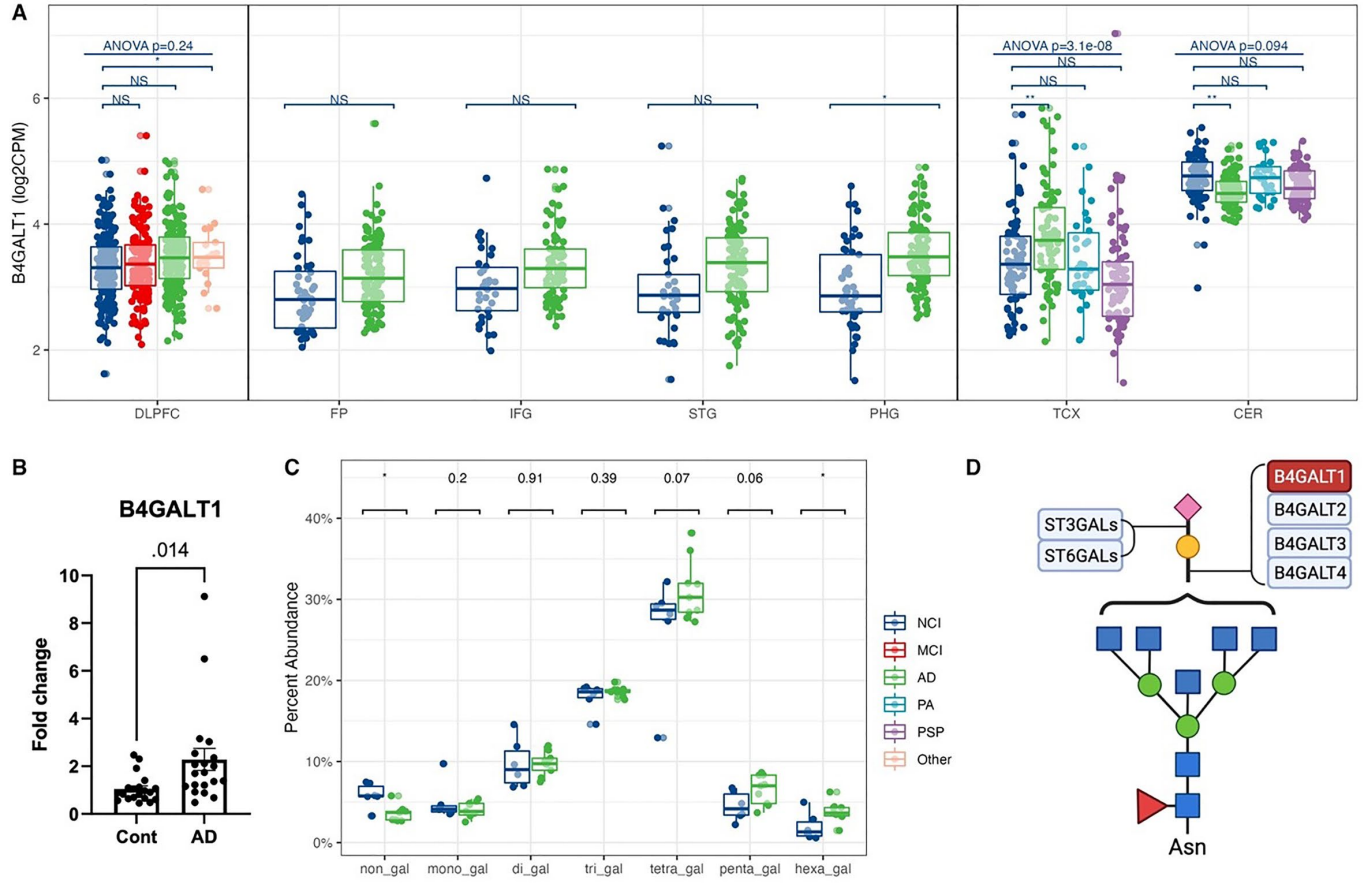
Increased B4GALT1 expression accompanied by a raised galactosylated *N*-glycans abundance in AD. (**A**) B4GALT1 gene expression from RNA-seq across human brains with different diagnoses. Data presented as log2 count per million (log2CPM). Data from different studies were separated into blocks. Adjusted p-values were calculated based on all protein-coding genes. NS p-value > 0.05, *p-value < 0.05 but adjusted p-value > 0.05, **p-value < 0.05 and adjusted p-value < 0.05. ANOVA p-values were after the multiple testing correction over all protein-coding genes. *DLPFC* dorsolateral prefrontal cortex, *FP* frontal pole, *IFG* inferior frontal gyrus, *STG* superior temporal gyrus, *PHG* parahippocampal gyrus, *TCX* temporal cortex, CER cerebellum. *NCI* no cognitive impairment, *MCI* mild cognitive impairment, *AD* Alzheimer’s disease, *PA* pathological aging, *PSP* progressive supranuclear palsy, *Other* other types of dementia. (**B**) qPCR confirmation of B4GALT1 expression in the medial temporal cortex (MTC) of AD and control participants. (**C**) The abundance of complex and hybrid *N*-glycans with different numbers of galactoses from *N*-glycomics in MTC of AD and control participants. *p-value < 0.05. (**D**) A schematic of galactosylation and sialylation steps. Red/blue boxes: over-/under-expressed in AD vs. control. Glycan symbol key: yellow circles, galactose (Gal); green circles, mannose (Man); blue squares, *N*-acetylglucosamine (GlcNAc); red triangles, fucose (Fuc); purple diamonds, *N*-acetylneuraminic acid (Neu5Ac).

The glycosylation process also involves elongation and branching biosynthetic steps. However, most glycosyltransferases for these steps belong to isoenzyme families with poorly understood non-redundant functions. We detected 17 glycosyltransferases for elongation and branching (Supplementary Fig. S3A). β-1,4-galactosyltransferase 1 (B4GALT1) adds terminal galactose to *N*-glycans to generate type 2 LacNAc units (Fig. 2D). The gene expression of B4GALT1 was substantially increased in AD participant brains in TCX (Fig. 2A, Supplementary Fig. S3A) and was not significantly changed in PA and PSP individuals, indicating that B4GALT1 may be an AD-specific target gene (Fig. 2A). The increased expression of B4GALT1 was also observed by qPCR in an independent set of tissue samples (Fig. 2B), showing approximately a twofold increase in gene expression in AD participants compared with controls. According to the glycomic data, the percent abundance of non-galactosylated *N*-glycans significantly decreased in AD participants in the MTC (Fig. 2C). Meanwhile, the proportion of *N*-glycans with six galactoses significantly increased in the MTC (Supplementary Fig. S3D), consistent with an increase in flux through the galactosylation pathway predicted by the increase in B4GALT1 expression detected by RNA-seq and qPCR. The terminal galactose residues on *N*-glycans can be further sialylated. The *N*-glycomic results also demonstrated increased total sialylation in the MTC from AD participants (Supplementary Fig. S3E). The increased sialylated *N*-glycans were mainly mono-sialylated. No significant *N*-glycan sialylation changes were observed in the LPFC and LCBC.

In addition to B4GALT1, the other two galactosyltransferases, B3GALT2 and B3GALT5, also significantly changed in AD in multiple brain regions, with results repeated by qPCR in the MTC (Supplementary Fig. S3B,C). β-1,3-GalTs generate type-1 LacNAc units, which are relatively high in *O*-glycans and glycosphingolipids of humans.

### Heterogeneous differential expression of isoenzymes involved in O-GalNAc-glycan initiation and alpha-2,6-sialylation on GalNAc residues

**Figure 3:**
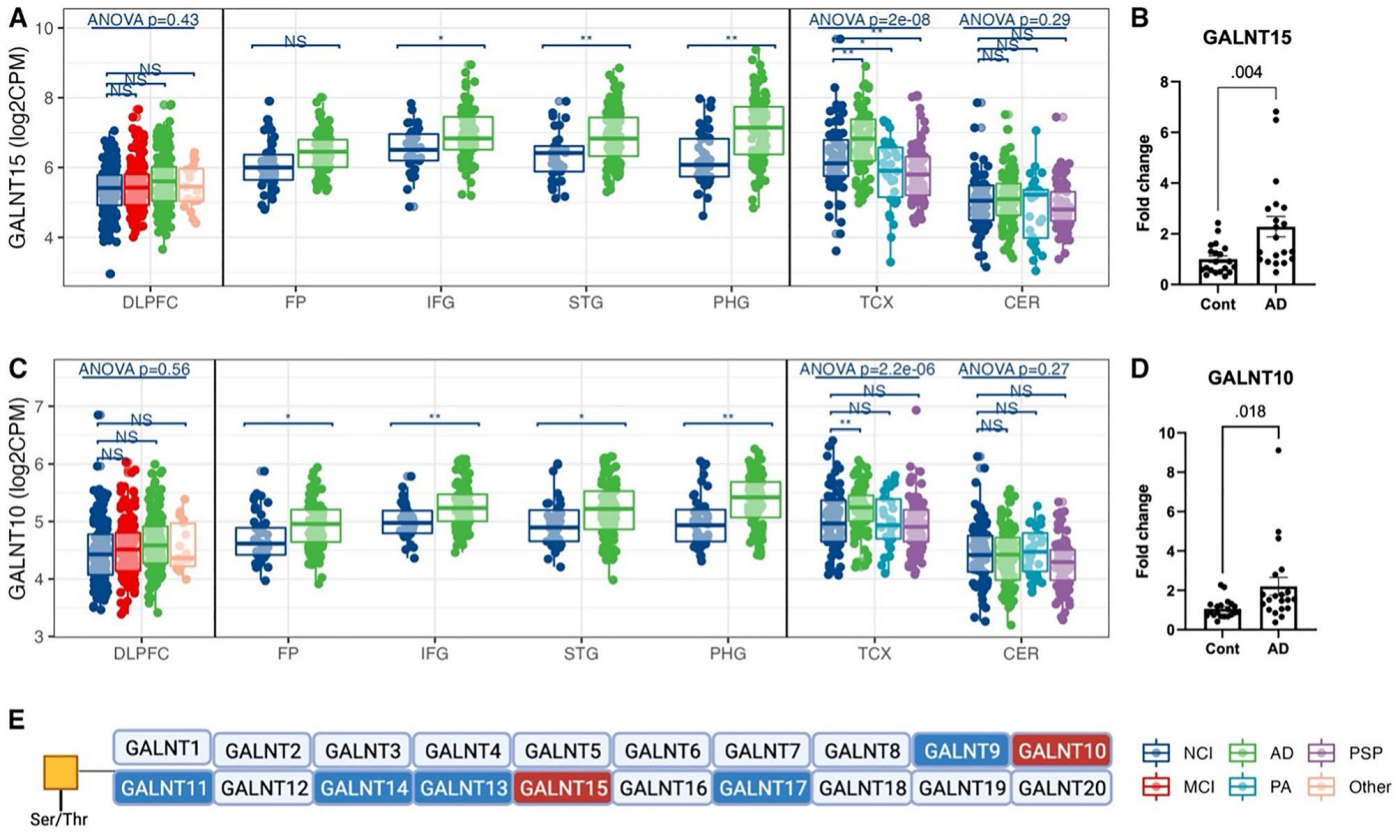
The differential expression of polypeptide *N*-acetylgalactosaminyltransferase (GALNT) gene family in AD across cortical regions. (**A**) GALNT15 gene expression from RNA-seq across human brains with different diagnoses. Data presented as log2 count per million (log2CPM). Data from different studies were separated into blocks. Adjusted p-values were calculated based on all protein-coding genes. NS p-value > 0.05, *p-value < 0.05 but adjusted p-value > 0.05, **p-value < 0.05 and adjusted p-value < 0.05. ANOVA p-values were after the multiple testing correction over all protein-coding genes. *DLPFC* dorsolateral prefrontal cortex, *FP* frontal pole, *IFG* inferior frontal gyrus, *STG* superior temporal gyrus, *PHG* parahippocampal gyrus, *TCX* temporal cortex, *CER* cerebellum. *NCI* no cognitive impairment, *MCI* mild cognitive impairment, *AD* Alzheimer’s disease, *PA* pathological aging, *PSP* progressive supranuclear palsy, *Other* other types of dementia. (**B**) qPCR confirmation of GALNT15 expression in the medial temporal cortex (MTC) of AD and control participants. (**C**) GALNT10 gene expression from RNA-seq across human brains with different diagnoses. (**D**) qPCR confirmation of GALNT10 expression in MTC of AD and control participants. (**E**) A schematic of the initiation step of *O*-GalNAc glycans. Red/blue boxes: over-/under-expressed in AD vs. control. Glycan symbol key: yellow squares, *N*-acetyl-galactosamine (GalNAc).

We observed heterogeneous differential expression within several notable isoenzyme families. For example, in the GALNT family of enzymes that initiate *O*-GalNAc-glycan biosynthesis (Fig. 3E), the gene expression of GALNT15 and GALNT10 increased, but the expression of GALNT11 and GALNT17 decreased in the cortical regions but not in the CER (Fig. 3A,C, Supplementary Fig. S4A–C) according to the RNA-seq analysis. Notably, the increased expression of these GALNT genes was unique to AD except for GALNT11 as they were not significantly different or changed in the opposite direction in MCI, PA, and PSP participants (Fig. 3A,C, Supplementary Fig. S4A–C). qPCR-based expression analysis in an independent sample set of tissues showed a twofold increase in gene expression of GALNT15 and GALNT10 but not GALNT11 in AD participants compared with controls (Fig. 3B,D, Supplementary Fig. S4D).

**Figure 4:**
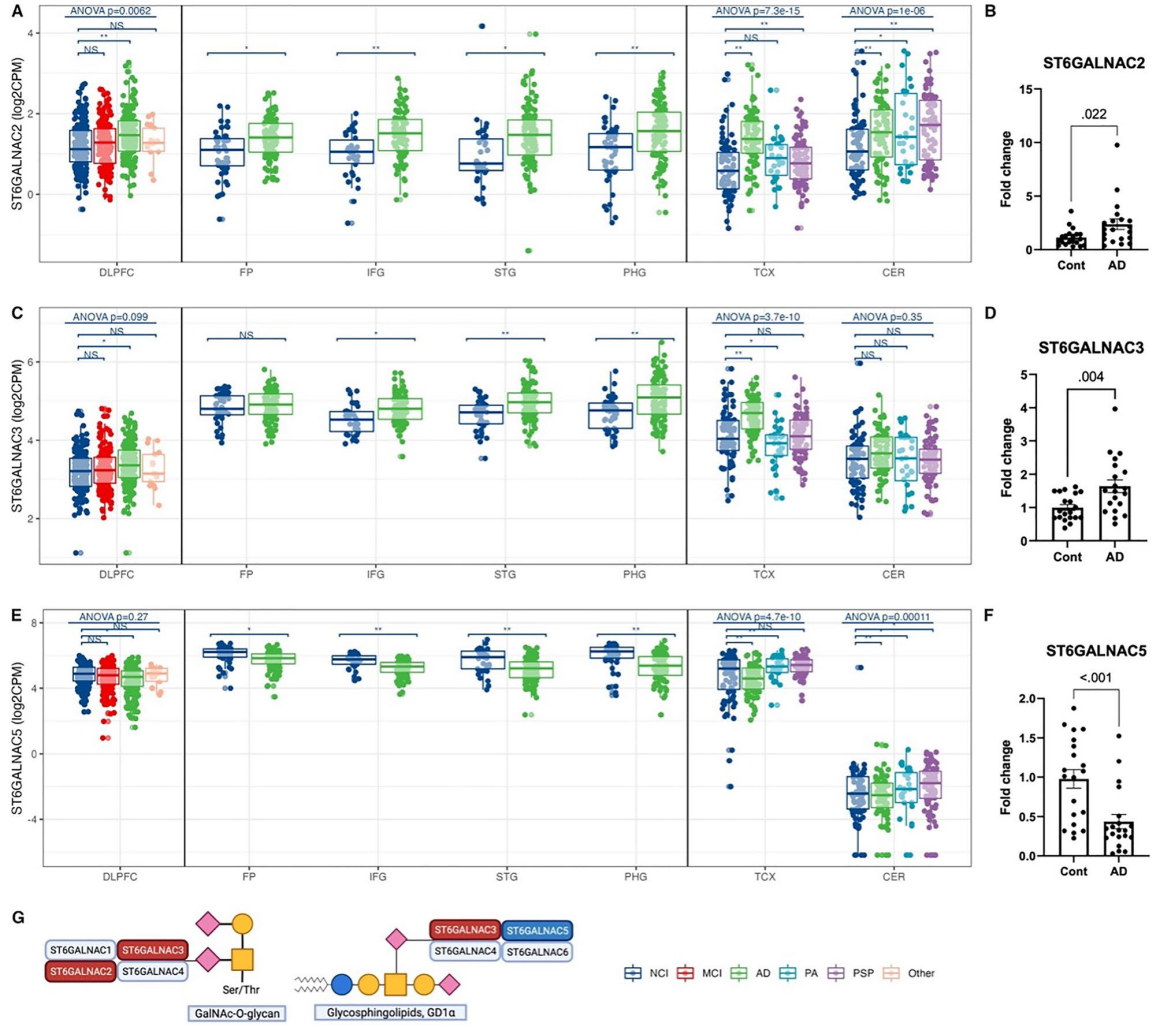
Differential expression of alpha-*N*-acetylgalactosaminide alpha-2,6-sialyltransferase (ST6GALNAC) gene family in AD across brain regions. (**A**) ST6GALNAC2 gene expression from RNA-seq across human brains with different diagnoses. Data presented as log2 count per million (log2CPM). Data from different studies were separated into blocks. Adjusted p-values were calculated based on all protein-coding genes. NS p-value > 0.05, *p-value < 0.05 but adjusted p-value > 0.05, **p-value < 0.05 and adjusted p-value < 0.05. ANOVA p-values were after the multiple testing correction over all protein-coding genes. *DLPFC* dorsolateral prefrontal cortex, *FP* frontal pole, *IFG* inferior frontal gyrus, *STG* superior temporal gyrus, *PHG* parahippocampal gyrus, *TCX* temporal cortex, *CER* cerebellum. *NCI* no cognitive impairment, *MCI* mild cognitive impairment, *AD* Alzheimer’s disease, *PA* pathological aging, *PSP* progressive supranuclear palsy, *Other* other types of dementia. (**B**) qPCR confirmation of ST6GALNAC2 expression in the medial temporal cortex (MTC) of AD and control participants. (**C**) ST6GALNAC3 gene expression from RNA-seq across human brains with different diagnoses. (**D**) qPCR confirmation of ST6GALNAC3 expression in the MTC of AD and control participants. (**E**) ST6GALNAC5 gene expression from RNA-seq across human brains with different diagnoses. (**F**) qPCR confirmation of ST6GALNAC5 expression in the MTC of AD and control participants. G) A schematic showing the function and specificity of ST6GALNACs. Red/blue boxes: over-/under-expressed in AD vs. control. Glycan symbol key: yellow circles, galactose (Gal); yellow squares, *N*-acetylgalactosamine (GalNAc); blue circles, *N*-glucose (Glc); purple diamonds, *N*-acetylneuraminic acid (Neu5Ac).

According to the RNA-seq results, in the ST6GALNAC family of enzymes, which transfer sialic acid onto *N*-acetylgalactosamine (GalNAc) in an alpha-2,6 linkage (Fig. 4G), ST6GALNAC2 and ST6GALNAC3 expression increased in AD participants, while ST6GALNAC5 decreased (Fig. 4A,C,E). ST6GALNAC3 and ST6GALNAC5 gene expression alterations were unique to AD participants and were also observed by qPCR in an independent set of samples (Fig. 4A–F). Notably, the expression level of ST6GALNAC5 was much lower in the CER than in the six cortical regions across all diagnosis groups (Fig. 4E). The gene expression of other isoenzyme families, such as the alpha-2,3-sialyltransferases (ST3GALs), alpha-2,6-sialyltransferases (i.e., ST6GALs), alpha-2,8-sialyltransferases (ST8SIAs), and fucosyltransferases (FUTs), were also altered in AD participants in multiple brain regions, with both increases and decreases in gene expression within the families. However, these results were either not confirmed by qPCR or observed in fewer brain regions (Supplementary Fig. S5A–D).

### Increased gene expression of key enzymes for GPI-anchor and galactocerebroside biosynthesis in AD participant brains

**Figure 5:**
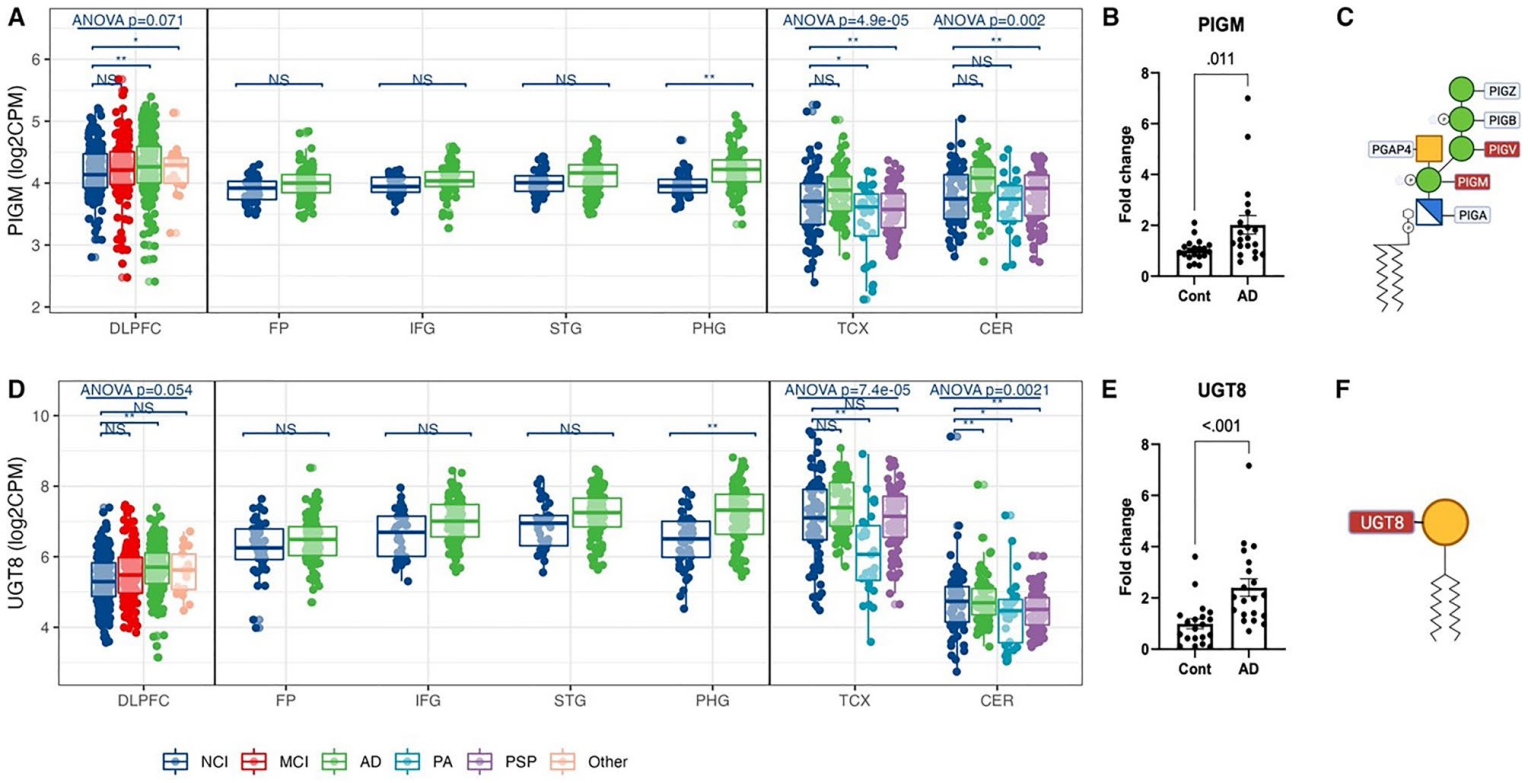
Differential expression of glycosyltransferases involved in GPI-anchor and glycosphingolipid biosynthesis in AD. (**A**) PIGM gene expression from RNA-seq across human brains with different diagnoses. Data presented as log2 count per million (log2CPM). Data from different studies were separated into blocks. Adjusted p-values were calculated based on all protein-coding genes. NS p-value > 0.05, *p-value < 0.05 but adjusted p-value > 0.05, **p-value < 0.05 and adjusted p-value < 0.05. ANOVA p-values were after the multiple testing correction over all protein-coding genes. *DLPFC* dorsolateral prefrontal cortex, *FP* frontal pole, *IFG* inferior frontal gyrus, *STG* superior temporal gyrus, *PHG* parahippocampal gyrus, *TCX* temporal cortex, *CER* cerebellum. *NCI* no cognitive impairment, *MCI* mild cognitive impairment, *AD* Alzheimer’s disease, *PA* pathological aging, *PSP* progressive supranuclear palsy, *Other* other types of dementia. (**B**) qPCR confirmation of PIGM expression in the medial temporal cortex (MTC) of AD and control participants. (**C**) The schema of the GPI-anchor biosynthesis. Red/blue boxes: over-/under-expressed in AD vs. control. (**D**) UGT8 gene expression from RNA-seq across human brains with different diagnoses. (**E**) qPCR confirmation of UGT8 expression in MTC of AD and control participants. (**F**) A schematic of galactocerebroside biosynthesis. Red/blue boxes: over-/under-expressed in AD vs. control. Glycan symbol key: yellow circles, galactose (Gal); yellow squares, *N*-acetylgalactosamine (GalNAc); green circles, mannose (Man); blue cross squares, glucosamine (GlcN).

In addition to glycosylation on proteins, we analyzed the enzymes specific to lipid glycosylation. Accroding to the RNA-seq results, the temporal regions had more altered genes than the frontal regions (Figure S6A). Among 14 detected lipid glycosylation genes, phosphatidylinositol glycan anchor biosynthesis class M (PIGM) and UDP glycosyltransferase 8 (UGT8) increased in DLPFC and PHG of AD participants compared with controls (Fig. 5A,D, Supplementary Fig. S6A). qPCR results confirmed a twofold increase in the expression of these genes in AD participants (Fig. 5B,E). Other critical genes were also upregulated in AD, such as PIGV (Supplementary Fig. S6A). Since PIGM and PIGV transfer the first and second mannose to the GPI backbone, increased expression may indicate increased biosynthesis of GPI-anchored structures in the brains of AD participants (Fig. 5C). Of the glycosphingolipid biosynthesis pathways, since UGT8 catalyzes a crucial step of galactocerebroside biosynthesis by transferring galactose to ceramide (Fig. 5F), increased UGT8 expression may cause an increase in galactocerebroside structures in cortical brain regions in AD participants.

Several genes involved in glucosylceramide biosynthesis were also changed in AD participants across multiple brain regions and confirmed by qPCR, including alpha 1,4-galactosyltransferase (A4GALT) and B4GALT6 (p-value = 0.05) (Supplementary Fig. S6B,D).

### Transcriptional and post-transcriptional regulation of glycosyltransferase genes

TFs and miRNAs regulate genes at the transcriptional and post-transcriptional level. We identified TF binding motifs over-represented on glycosyltransferase gene sets. The TF STAT1 had the highest Normalized Enrichment Score (NES) (NES = 5.28) for *N*-glycan core structure biosynthesis. MGAT1, MGAT3, MGAT4B, FUT8 and other genes were highly enriched for this TF binding motif (Supplementary Table S6, Supplementary Fig. S7A). ZNF595, E2F7, ELK3, FOXO1, RXRA and other TFs were also identified as potential transcriptional regulators interacting with genes in the N-glycosylation pathway. The TF HSF5 was the most significant regulator (NES = 6.01) for the elongation and branching pathways. Eight genes including B3GALT2, B3GALT5 were highly enriched for this binding motif (Supplementary Table S6, Supplementary Fig. S7B).

Using databases containing experimentally validated miRNA-target interactions, we identified miRNAs that possibly regulate various glycosylation pathways (Supplementary Table S7). For *N*-glycan biosynthesis, hsa-miR-1-3p and hsa-miR-124-3p regulated 21 and 16 out of 28 glycosyltransferases in this pathway, respectively. The targets of both miRNAs included MGAT1, MGAT2, MGAT4A/B, FUT8, and other genes (Supplementary Table S7, Supplementary Fig. S8A). For elongation and branching, hsa-miR-16-5p (n = 7) and hsa-miR-34a-5p (n = 6) regulated the highest number of glycosyltransferases in the pathway. B4GALT1 and other genes were regulated by these miRNAs (Supplementary Table S7, Supplementary Fig. S8B).

## Discussion

The brain is a complex organ with multiple brain regions performing distinct functions, such as memory, motor function, language, and learning, making it difficult to disentangle the pathogenesis and pathology of AD. Integrating publicly available RNA-seq data with qPCR and LC–MS/MS-based glycomics in separate sample sets, we performed a large-scale overview of the major alterations in N- and O-glycosylation, as well as lipid glycosylation in the context of AD across multiple human brain regions. We further identified candidate TFs and miRNAs that regulate the expression of glycosyltransferases for different glycosylation pathways.

In this study, we identified 13,882 protein-coding genes differentially expressed in at least one brain region, with the highest number of genes observed in TCX (n = 9271) and CER (n = 8113), followed by PHG (n = 4545) and IFG (n = 3275), and the lowest number of DEGs in FP (n = 154). The DEGs identified in each brain region were found to be unique, especially in TCX and CER, which had 2096 and 2396 unique DEGs, respectively (Supplementary Fig. S1A). Our results are comparable with previous studies that reported the presence of 2174, 559, and 71 DEGs in the prefrontal cortex (PFC), precuneus (PRE), and visual cortex (VIC), respectively^23,24^. These cortical regions range from severely to mildly affected by tau pathology and neuronal loss. We found that 2027 DEGs in PFC overlapped with one or multiple brain regions in our study (Supplementary Fig. S9). A previous analysis using transcriptomic data from the ROSMAP, MSBB, and Mayo data sets revealed 4485 DEGs with a fixed meta-analysis model^25^.

Among the DEGs, in this study we specifically focused on glycosyltransferase genes. Systematic studies on the differential expression of glycosylation-related genes in AD are limited. However, several papers focused on the glycosyltransferases involved in specific pathways. A previous study reported an increase in GALNT4/6/7/8/10 expression in AD^15^, while another study found upregulation of STT3B, MAGT1, ALG2/6/12, and downregulation of RPN2, DAD1, OST4, OSTC, TUSC3, ALG3/9/12 in AD^10^.

In this study, an increase in flux through the complex *N*-glycan synthesis pathway which could be predicted by an increase in the expression of the MGAT1 enzyme by both RNA-seq and qPCR, was corroborated by *N*-glycomic results showing decreases in high-mannose structures and concomitant increases in complex *N*-glycans in an independent set of brain tissues, procured from a separate cohort of AD participants vs. controls. High-mannose *N*-glycans are usually considered the precursor of hybrid and complex *N*-glycans. In the Golgi apparatus, the high-mannose structure is further trimmed and modified to form hybrid and complex *N*-glycans. MGAT1 initiates the biosynthesis of hybrid and complex structures. Decreased high-mannose *N*-glycans were also observed in the cerebrospinal fluid (CSF) of both males and females with AD^26^. Some studies have reported that complex *N*-glycans are essential for normal neurological development as MGAT1 defective mouse embryos failed in neural tube formation or died within eight weeks of birth^13,27^. Neuron-specific loss of MGAT1 function resulted in reduced complex and increased high-mannose *N*-glycans in hippocampal regions and in the spinal cord, concurrent with neuronal apoptosis and astrogliosis^13^. The most abundant *N*-glycan subtype observed in human brains in this study was complex *N*-glycans (~ 75% of total measured structures), which confirmed some previous studies^28^. However, other studies using different glycomic analytical approaches have reported the most abundant structures found were high mannose structures^7,29^.

Our findings also demonstrated increased galactosylated *N*-glycans in AD participant brain samples, which would be consistent with an increase in flux through the galactosylation pathway predicted by both RNA-seq and qPCR. B4GALT1 is a galactosyltransferase enzyme which adds galactose residues to *N*-glycans. Although few studies have investigated the role of B4GALT1 in AD, in rats after spinal cord injury, B4GALT1 protein partly colocalized with microglia, oligodendrocytes, and astrocytes and mediated pro-inflammatory reactions^30^. Conversely, B4GALT1 knockdown in mice resulted in an inhibition of glioblastoma development and increased survival^31^. Together this evidence suggests that galactosylation, particularly galactosylation mediated by B4GALT1, may be involved in regulating pro- vs. anti-inflammatory events in the central nervous system.

Studies using knockout models of glycogenes in animals and cell lines have demonstrated the presence of specific glycogenes can cause changes in the glycome^32,33^. Although direct evidence for specific regulation of the glycome by the expression of a single glycosyltransferase is limited, transcriptional regulation of the enzymes directly involved in glycan biosynthesis is likely to be the easiest way to study the regulation of the glycome^34,35^. In a previous glycomic study, the authors identified 62 glycosylation genes differentially expressed between the cortex and cerebellum and reported the correlation between these genes and glycomic patterns^29^. For example, the expression of Mgat3 was higher than the expression of other Mgat enzymes, consistent with a high abundance of bisecting *N*-glycans and a low abundance of complex, branched *N*-glycans. Moreover, Zhang et al. conducted a glycoproteomic study in which they compared their *N*-glycoproteomic data to four different transcriptome datasets for brain tissues^10^. They suggested that altered expression of enzymes in dolichol-oligosaccharide biosynthesis could contribute to the observed protein N-glycosylation changes in the AD brain.

An additional finding in this study is that isoenzyme-specific changes in enzymes that initiate O-glycosylation and sialylation, particularly those involving the GALNT and ST6GALNAC isoenzyme families respectively, may be involved in AD. Although the isoenzymes belonging to these isoenzyme families catalyze the same reactions (i.e. all the GALNTs initiate O-glycosylation and all the ST6GALNACs add an alpha-2,6 sialic acid), several studies have reported their specificity for protein substrates in SimpleCell lines^36–38^, emphasizing the need to study the non-redundant functions of these enzymes in different cell types under different conditions. The limited knowledge of isoenzyme non-redundant function also partly hampers the ability to correlate the abundance changes of glycan structures with differential expression. On the other hand, although most GALNTs and some ST6GALNACs have been studied in terms of their structure and enzyme kinetics^39–43^, their physiological role in the central nervous system is still limited. Furthermore, their protein targets have not been defined. The heterogeneous AD-associated differential expression of these isoenzymes suggests a precisely regulated, pathway-specific process, with specific isoenzymes acting on different cell types, proteins, or glycosylation sites.

In addition to ST6GALNACs for alpha-2,6 sialylation, the capping process also involves alpha-2,3 sialyltransferases, alpha-2,8 sialyltransferases, and fucosyltransferases. In this study, the gene expression of these other sialylatransferases and fucosyltransferases was not as highly differential or was not changed in as many brain regions as the ST6GALNACs in AD participant brains. Sialylation has been extensively investigated in neuroinflammation. CD33 is a sialic acid-binding immunoglobulin-like lectin (Siglec) expressed in microglia in the brain^44^. The CD33 gene is a genetic risk factor for AD in GWAS studies, with a rare allele leading to decreased expression of CD33M (major isoform) and increased expression of CD33m (minor isoform), which is associated with protection from AD^45,46^. CD33 overexpression in the human brain is associated with impaired cognition^45,47,48^. When alpha-2,3 and, preferentially, alpha-2,6 sialylated glycans bind CD33, the Aβ phagocytosis activity of microglia increases^11,49,50^. Importantly, ST6GALNACs only modify GalNAc residues found on *O*-glycans and glycosphingolipids, highlighting the need for future studies focused on the sialylation changes in *O*-glycans and glycosphingolipids in AD.

Additionally, we observed alterations in glycosphingolipid pathways, where gene expression of UGT8, PIGM, and PIGV for galactocerebroside and GPI-anchor biosynthesis changed in DLPFC and PHG. In Parkinson’s disease glycosphingolipid could control neuroinflammation and its metabolism was deregulated with decreased glucocerebrosidase activities in the brain of participants compared to control^51,52^. UGT8 initiates the biosynthesis of galactosylceramides. However, most glycosphingolipids in the mammalian brain derive from glucosylceramides, the precursor of complex gangliosides, including the four major gangliosides in the brain, GM1, GD1a, GD1b, and GT1b^52,53^. The changes in glycosphingolipid patterns are cell-specific as glucosylceramide-derived gangliosides are enriched in neurons, and galactosylceramides are enriched in oligodendrocytes and myelin^52–54^. The increased gene expression of UGT8 observed in this study highlights the involvement of oligodendrocytes and myelin in AD pathogenesis. In addition, in this study we identified increased gene expression of both PIGA and PIGM, which are both involved in GPI anchor formation, in the brains of AD participants. The GPI anchor attaches the immunoglobulin superfamily of cell adhesion modules (IgSF CAMs) to plasma membranes on neurons and functions in synapse formation, synaptic plasticity, and cell-to-cell communication^55,56^. Our findings support further research to better understand the involvement of glycolipid pathways in the pathogenesis of AD.

Finally, we identified several candidate regulators, including TFs and miRNAs that potentially regulate glycosyltransferase gene expression. STAT1 was a critical TF for glycosyltransferases, regulating MGAT1, MGAT3, FUT8, and other genes in the N-glycosylation pathway. STAT1 is one of the key regulators for inflammatory activation within microglia in AD brains^57,58^. Its expression impaired spatial learning and memory in rats and could be induced by Aβ in a dose-dependent manner^59^. Our study also identified hsa-miR-1-3p, hsa-miR-124-3, hsa-miR-16-5p, and hsa-miR-34a-5p as the top candidate miRNA regulators for genes involved in N-glycosylation and elongation. hsa-miR-124 and hsa-miR-34a are two of the most abundantly expressed miRNAs in mammalian brains. Studies have demonstrated that hsa-miR-124-3p suppressed B4GALT1 protein translation in multiple cell lines^60^. Moreover, the expression of hsa-miR-124 gradually decreased in AD, which led to up-regulated BACE1 expression, ultimately responsible for Aβ production^61,62^. hsa-miR-34a has been reported to regulate ALG13, FUT8, GALNT7, and ST3GAL5 in neuroblastoma and hepatocellular carcinoma^63–66^. It ubiquitously participates in neurodevelopment and neuropathological processes and is dysregulated in AD. Its overexpression in animal models of AD was associated with neuronal death, and the suppression of hsa-miR-34a was neuroprotective^67^. In addition, hsa-miR-34a-5p has been found to be dysregulated in AD and other neurodegenerative diseases^68^. hsa-miR-16-5p was upregulated by Aβ in AD and induced neuronal apoptosis^69^. Our analysis was able to capture glycosyltransferase genes and their putative transcriptional and posttranscriptional regulators. These findings highlight the key glycosylation-related pathways and their regulators that could be important for the discovery of potential new targets for the management, treatment and prevention of AD. However, these findings need to be further validated in future experiments.

In summary, we have developed a system-level view of alterations in glycosylation-related genes in multiple brain regions in the context of AD and identified putative regulators of glycosyltransferases from different pathways. Strengths of the study include investigation of RNA-seq data from multiple well-characterized AD cohorts spanning multiple brain regions, and the fact that several of the differential gene expression results found by RNA-seq are supported by qPCR in an independent set of brain samples. Given that typically 15–20% of protein-coding genes are non-concordant between RNA-seq and qPCR even within the same samples, our orthogonal findings in an independent sample set strengthen the likelihood that the observed changes may be important in AD^70^. We further studied the *N*-glycome with MS-based analysis in another independent set of samples. As expected, the transcriptomic alterations do not completely agree with the glycosylation changes observed by LC–MS/MS-based glycan analysis. This discrepancy may be caused by several other factors that potentially contribute to glycomic changes, including alterations in the expression of the most abundant glycoproteins in the brain, post-transcriptional and post-translational modifications of glycosyltransferase genes, donor sugar availability, competition among enzymes, co-factors, pH, chaperones and glycosidases, as well as glycome measurement methods. However, the fact that we found strong support for the transcription-level changes by measuring glycans directly in brain tissue homogenates indicates that these changes in glycosylation are consistent and important in AD etiology. There are also several critical limitations of this study. First, and most importantly, cellular heterogeneity in gene expression can be masked with the bulk RNA-seq approach. It is well appreciated that brain tissue homogenates from different development periods and regions of the brain comprise different relative abundances of cell types^71^. Furthermore, because neurons and oligodendrocytes represent the majority of cells in any given brain tissue homogenate, bulk RNA-seq, qPCR and glycomic analysis is most likely to yield information about these most abundant cells, and changes in less abundant cell types (e.g. microglia) are most likely to be masked all together. Even further changes can be apparent between different subsets or phenotypes of cells (e.g. glutaminergic vs. GABAergic neurons, disease-associated microglia vs. resting microglia), and these also cannot be observed with the bulk approach in this study. Future studies are needed to address these important differences, including single-cell RNA-seq analysis, and various approaches to enrich specific cell types prior to gene expression and/or glycan analysis. Additionally, the differences in glycan profiles using different glycomic methods should be considered^72^. Future glycoproteomic studies are needed to investigate the abundance changes of glycosylated proteins to confirm whether the glycan changed observed here are due to the changes of the most abundant glycoproteins carrying those glycans. Site-specific glycoprofiles are also required in addition to the overall glycosylation patterns of cleaved glycans, since the glycosylation of proteins at different sites affect different aspects of protein function, metabolism, and structure. The association between sialylation, in particular alpha-2,6-sialylation, and neuroinflammation mediated by siglecs expressed in microglia and other cell types may also be an important future area of research. The findings of our current study, which provides a broad overview of key alterations in glycosylation pathways in the brain in AD participants and their putative regulators, support further research of glycosylation-related pathways to understand the pathology of AD.

## Conclusion

The present study implies pathway-specific glycosylation alterations in the brains of AD dementia individuals. Differentially expressed glycosyltransferases mediate the aberrant brain glycan profile. And the putative transcriptional and post-transcriptional regulators for glycosyltransferases involved in the *N*-glycan core structure biosynthesis, elongation, and other pathways potentially facilitate the future development of AD therapeutics.

## Supplementary Information

Supplementary Information 1.

Supplementary Information 2.

Supplementary Information 3.

## Data Availability

The bulk RNA-seq datasets analyzed during the current study are available in the Accelerating Medicines Partnership Alzheimer’s Disease Project (AMP-AD) Knowledge Portal under the RNAseq Harmonization Study (rnaSeqReprocessing), https://www.synapse.org/#!Synapse:syn17010685, but an access request is required. The N-glycomic datasets generated and analyzed are included in the supplementary information files of this article. Mass spectrometry data for glycomics (https://doi.org/10.25345/C5J67920T, MSV000090677) is available on MassIVE data repository upon reasonable request (contact: xctang@ucdavis.edu).

## Abbreviations

AD: Alzheimer’s disease
MCI: Mild cognitive impairment
NCI: No cognitive impairment
MGAT1: Alpha-1,3-mannosyl-glycoprotein 2-beta-*N*-acetylglucosaminyltransferase
B4GALT: β-1,4-Galactosyltransferase 1
GALNT: Polypeptide *N*-acetylgalactosaminyltransferase
ST6GALNAC: Alpha-*N*-acetylgalactosaminide alpha-2,6-sialyltransferase
UGT8: UDP glycosyltransferase 8
PIGM: Phosphatidylinositol glycan anchor biosynthesis class M
STAT1: Signal transducer and activator of transcription 1
HSF5: Heat shock transcription factor 5
DLPFC: Dorsolateral prefrontal cortex
FP: Frontal pole
IFG: Inferior frontal gyrus
STG: Superior temporal gyrus
PHG: Parahippocampal gyrus
TCX: Temporal cortex
CER: Cerebellar cortex
MTC: Medial temporal cortex
LPFC: Lateral prefrontal cortex
LCBC: Lateral cerebellar cortex
TF: Transcription factors
TSS: Transcription start site
NES: Normalized Enrichment Score
Aβ: Amyloid β
APP: Amyloid precursor protein
MSBB: Mount Sinai Brain Bank
ROSMAP: Religious orders study/memory and aging project
