# Supplementary material for: Transcriptomic and glycomic analyses highlight pathway-specific glycosylation alterations unique to Alzheimer’s disease
Source: Sci Rep. 2023 May 15;13:7816. doi: 10.1038/s41598-023-34787-4 (PMC10185676; doi:10.1038/s41598-023-34787-4)
Supplement: Supplementary file 1 — Supplementary Information 1. [file 41598_2023_34787_MOESM1_ESM.docx]

Supplemental Data:

**Transcriptomic and glycomic analyses highlight pathway-specific glycosylation alterations unique to Alzheimer’s disease**

**Authors**

Xinyu Tang^1*^, Jennyfer Tena^2*^, Jacopo Di Lucente^3,4^, Izumi Maezawa^3,4^, Danielle Harvey^5^, Lee-Way Jin^3,4^, Carlito B. Lebrilla^2^, Angela M. Zivkovic^1**^

**Institutions**

^1^Department of Nutrition, University of California, Davis, Davis, CA, USA

^2^Department of Chemistry, University of California, Davis, Davis, CA, USA

^3^Department of Pathology and Laboratory Medicine, School of Medicine, University of California, Davis, Sacramento, CA, USA

^4^UC Davis MIND Institute, Sacramento, CA, USA

^5^Division of Biostatistics, Department of Public Health Sciences, School of Medicine, University of California, Davis, Davis, CA, USA

*These two authors have equal contribution

**Corresponding author: Angela M. Zivkovic, email: [amzivkovic@ucdavis.edu](mailto:amzivkovic@ucdavis.edu), One Shields Ave, Department of Nutrition, University of California, Davis, Davis, CA, USA

Table of Contents

[Supplementary Methods 3](#_Toc132644672)

[*Public RNA-seq data* 3](#_Toc132644673)

[*RNA-seq data normalization* 4](#_Toc132644674)

[*Differential expression analysis of public RNA-seq datasets* 4](#_Toc132644675)

[*Human Brain Tissue Collection* 5](#_Toc132644676)

[*Quantitative real-time PCR* 5](#_Toc132644677)

[*Cell Membrane Extraction* 6](#_Toc132644678)

[*Enzymatic Release and Purification of N-Glycans* 6](#_Toc132644679)

[*Glycomics Analysis by LC-MS/MS* 7](#_Toc132644680)

[*Transcription Factors and miRNA-target Interaction Inference* 8](#_Toc132644681)

[*Statistical Analysis* 8](#_Toc132644682)

[Supplementary Tables 9](#_Toc132644683)

[Supplemental Table S1: Samples size information. 9](#_Toc132644684)

[Supplemental Table S2: Demographic characteristics of the ROSMAP, MSBB, and Mayo Alzheimer’s disease cohorts. 10](#_Toc132644685)

[Supplemental Table S3: qPCR primer information. 14](#_Toc132644686)

[Supplemental Table S4: Demographic data of participants in the glycomic analysis. 15](#_Toc132644687)

[Supplemental Table S5: Glycosyltransferases excluded for low expression. 16](#_Toc132644688)

[Supplemental Table S6: Potential transcription factors and their target genes. 17](#_Toc132644689)

[Supplemental Table S7: miRNAs targeting the genes of glycosyltransferases according to validated databases (miRecords, miRTarBase and TarBase). 17](#_Toc132644690)

[Supplementary Figures 18](#_Toc132644691)

[References 29](#_Toc132644692)

# **Supplementary Methods**

## ***Public RNA-seq data***

RNA-seq data in this study originated from the MSBB, Mayo, and ROSMAP studies. They were obtained from the RNA-seq Harmonization Study (<https://adknowledgeportal.synapse.org/Explore/Studies/DetailsPage?Study=syn9702085>), which harmonized RNA-seq data from all three studies by re-processing the reads with a uniform pipeline.

The MSBB study[1] has RNA-seq data from four brain regions: frontal pole (FP, Brodmann area 10, n=162), superior temporal gyrus (STG, Brodmann area 22, n=152), parahippocampal gyrus (PHG, Brodmann area 36, n=159), and inferior frontal gyrus (IFG, Brodmann area 44, n=125). Since MSBB didn’t provide the diagnoses, we referred to the classification in a publish paper[2]. Participants with Clinical Dementia Rating (CDR) <= 0.5, CERAD = 1, and Braak score <= 3 were identified as having no cognitive impairment (NCI). Participants with CDR > 0.5, CERAD > 1, and Braak score > 3 were defined as having Alzheimer’s Disease (AD).

The ROSMAP study[3] has RNA-seq data from the dorsolateral prefrontal cortex (DLPFC) from 581 participants who are categorized as having NCI, mild cognitive impairment (MCI), AD, and other types of dementia (Other).

The Mayo RNA-seq study[4] has data from the temporal cortex (TCX) and cerebellum (CER) from 274 and 271 participants respectively, and who are categorized as having NCI, pathological aging (PA), progressive supranuclear palsy (PSP), and AD. The sample size information is summarized in **Table S1**.

## ***RNA-seq data normalization***

Only protein-coding genes were used for analysis. Low-expressed genes were dropped using the filterByExpr() function from the edgeR package [5] in R version 4.1.0 (R Foundation for Statistical Computing, Vienna, Austria). The filter step keeps genes that have count-per-million (CPM) above 10 in 70% of samples. The library sizes were recalculated after filtering. Sample-specific effects were removed by normalizing library sizes with the trimmed mean of M-values (TMM) method using the calcNormFactors() function from the edgeR package.

## ***Differential expression analysis of public RNA-seq datasets***

A negative binomial model with the quasi-likelihood (QL) F-test in edgeR package was applied to perform the differential expression analysis among diagnosis groups. First, gene counts with normalized library sizes were fitted to a negative binomial generalized linear model adjusted for sex, age, and RNA Integrity Number (RIN), post-mortem interval (PMI), and batch. Due to the large missing values, the model for Mayo didn’t include PMI and batch as covariates. Then, the dispersion was estimated using the estimateDisp() function in edgeR. The QL dispersion estimation was calculated using the glmQLFit() function, followed by the glmQLFTest() function that conducted the quasi-likelihood (QL) F-test. Finally, we used the topTags() function to output significantly differentially expressed genes. For datasets with more than two diagnosis groups, we first performed an ANOVA-like test for any differences between diagnosis groups with coefficients related to the diagnosis. Then pairwise comparisons between disease and control groups (NCI) were performed. P-values were adjusted for multiple hypothesis testing using Benjamini & Hochberg (BH) False Discovery Rate (FDR). Genes with an adjusted p-value below or equal to 0.05 were identified as differentially expressed genes. Genes with a raw p-value below or equal to 0.05 but adjusted p-value greater than 0.05 were identified as probably differentially expressed genes.

## ***Human Brain Tissue Collection***

Human postmortem brain samples were drawn from the brain repositories of UC Davis Alzheimer’s Disease Research Center (ADRC). Written informed consent, including consent for autopsy, was obtained from study participants or, for those with substantial cognitive impairment, a caregiver, legal guardian or other proxy. Study protocols were reviewed and approved by the Institutional Review Board (IRB). For postmortem diagnosis, we followed the National Institute on Aging-Alzheimer’s Association guideline for the neuropathologic assessment of AD[6]. The samples for qPCR were from the medial temporal cortex (MTC). The specimens for the N-glycomics were from the MTC, lateral prefrontal cortex (LPFC), and lateral cerebellar cortex (LCBC). The brain tissue used for the current study was snap frozen during autopsy and was stored in -80 °C before RNA extraction.

## ***Quantitative real-time PCR***

Total RNA from medial temporal cortex (MTC) tissue samples (20 ADs vs. 20 controls) were extracted using RNeasy® Plus Universal Mini Kit (Qiagen, Valencia, CA) according to manufacturer’s protocol. cDNA was synthesized using iScript Reverse Transcription Supermix (Bio-Rad, Hercules, CA). RNA purity and concentrations were assessed by measuring the absorbance at 260 nm, and 280 nm through a NanoDrop 2000C Spectrophotometer (Thermo Scientific, Waltham, MA). Quantitative PCR (qPCR) was performed using the Sso Fast EvaGreen Supermix (Bio-Rad) in the CFX96 Touch Real-Time PCR Detection System (Bio-Rad). The primer sequences used are listed in **Table S2**. Gene expression was normalized to an endogenous reference gene, β-actin. Data were analyzed by the 2-ΔΔCt method. All experiments were performed in duplicate.

## ***Cell Membrane Extraction***

A total of 51 samples from 20 participants (10 ADs vs. 10 controls) (**Table S3**), collected from LCBC, LPFC, MTC, were analyzed. All tissue samples were obtained from the brain repositories of UC Davis ADRC. Tissue samples were homogenized and resuspended in homogenization buffer containing 0.25 M sucrose, 20 mM HEPES-KOH (pH 7.4), and a 1:100 protease inhibitor cocktail. Cells were lysed on ice using a probe sonicator operated with alternating on and off pulses of 5 and 10 s, respectively. Lysates were pelleted by centrifugation at 2000xg for 10 min to remove the nuclear fraction and cell debris. The supernatant was transferred to high speed tubes, loaded onto a Beckman Optima TLX Ultra-centrifuge at 4˚C, and centrifuged at 200,000 x g for 45 min in series to remove other nonmembrane subcellular fractions. The resulting cell membrane pellet was stored at 20˚C until further processing.

## ***Enzymatic Release and Purification of N-Glycans***

Proteins were suspended with 100 µL of 100 mM NH_4_HCO_3_ in 5 mM dithiothreitol and heated at 100˚C for 10s to thermally denature the proteins. To release the glycans, 2 µL (500,000 units/ml) of peptide N-glycosidase F (glycerol-free; New England BioLabs, cat. no. P0705L) were added to the samples, followed by incubation in a microwave reaction at 60°C for 10 min to accelerate N-glycans release. Samples were incubated for 18 h at 37°C to hydrolyze the N-glycans. The reaction was quenched with 350 μL of water followed by ultracentrifugation at 200,000 x g to separate the N-glycans and the membrane fraction (MF) containing our lipids and de-glycosylated proteins. The released N-glycans were purified by solid-phase extraction using porous graphitized carbon (PGC) packed cartridges. The cartridges were first equilibrated with nanopure water and a solution of 80% (v/v) acetonitrile and 0.05% (v/v) trifluoroacetic acid in water. The dried samples were solubilized, loaded onto the cartridge, and washed with nanopure water to remove salts and buffer. N-Glycans were eluted with a solution of 40% (v/v) acetonitrile and 0.05% (v/v) trifluoroacetic acid in water, dried and reconstituted in 30µl of water prior to mass spectrometric analysis.

## ***Glycomics Analysis by LC-MS/MS***

Purified brain N-glycans were analyzed using an Agilent nano-LC/chip Q-ToF MS system. The nano-LC system employs a binary solvent consisting of A (0.1% formic acid in 3% acetonitrile in water (v/v)) and B (0.1% for-mic acid in 90% acetonitrile in water (v/v)). Samples were enriched and separated on the Agilent HPLC-Chip comprised of a 40 nL enrichment column and a 75 μm x 43 mm ID analytical column both packed with porous graphitized carbon in 5 μm particle size. The sample was delivered by the capillary pump to the enrichment column at a flow rate of 3 μL/min and separated on the analytical column by the nano-pump at a flow rate of 0.3 μL/min with a gradient that was previously optimized for N-glycans: 0% B, 0-2.5 min; 0-16% B, 2.5-20 min; 16-44% B, 20-30 min; 44-100% B, 30-35 min; and 100% B, 35-45 min followed by pure A for 20 min of equilibration. MS spectra were acquired at 1.5 s per spectrum over a mass range of m/z 600–2000 in positive ionization mode. Mass inaccuracies were corrected with reference mass m/z of 1221.991.

N-Glycan compositions were identified using MS and MS/MS data as well as an in-house retrosynthetic library based on the mammalian N-glycan biosynthetic pathway. Deconvoluted masses were compared to theoretical masses using a mass tolerance of 20 ppm and a false discovery rate of 0.5% on the Agilent MassHunter software version B.7. Relative abundances were determined by integrating peak areas for observed glycan masses, averaging abundances from instrumental triplicates and normalizing to the summed peak areas of all glycans detected.

## ***Transcription Factors and miRNA-target Interaction Inference***

Transcription factors (TF) and their binding motifs of glycosyltransferases were identified using the RcisTarget R package [7]. We used the motif collection based on human hg38 genome assembly and RefSeq genes and searched 10kbp around the transcription start site (TSS). Motifs with normalized enrichment scores (NES) greater than 3 were considered significant. I-cisTarget method was used to identify genes highly ranked for each significant motif.

MicroRNAs and their interactions with glycosyltransferases were inferred based on three databases with experimentally validated miRNA-target interactions: miRecords, miRTarBase, and TarBase. The information was retrieved using the multiMiR package [8].

## ***Statistical Analysis***

For normally distributed data, groups were compared using Student’s t-test. The fractions of subtypes, sialylated and fucosylated N-glycans were calculated as the sum of relative abundances of single glycans. Since we obtained more than one brain tissue from the same participant, differential abundance analysis was performed as linear mixed models using the lmerTest package in R[9] as shown here: glycan abundance ~diagnosis*region+(1|subject_id), where subject-specific effects were modeled as random effects. Contrasts between AD and control diagnosis were evaluated for each brain region. These contrasts produced t-statistics and corresponding p-values.

# **Supplementary Tables**

**Supplemental Table S1**: Samples size information.

| AD Study | Tissue | Diagnosis | | | | | | Total Samples |
| --- | --- | --- | --- | --- | --- | --- | --- | --- |
|  |  | NCI | MCI | AD | PA | PSP | Other |  |
| ROSMAP | DLPFC | 195 | 161 | 208 | - | - | 17 | 581 |
| MSBB | FP | 48 | - | 114 | - | - | - | 162 |
|  | IFG | 34 | - | 91 | - | - | - | 125 |
|  | STG | 37 | - | 115 | - | - | - | 152 |
|  | PHG | 49 | - | 110 | - | - | - | 159 |
| Mayo | TCX | 78 | - | 82 | 30 | 84 | - | 274 |
|  | CER | 77 | - | 82 | 28 | 84 | - | 271 |
| Total |  | 518 | 161 | 802 | 58 | 168 | 17 | 1724 |

Notes: DLPFC dorsolateral prefrontal cortex, FP frontal pole, IFG inferior frontal gyrus, STG superior temporal gyrus, PHG parahippocampal gyrus, TCX temporal cortex, CER cerebellum. NCI: no cognitive impairment; MCI: mild cognitive impairment; AD: Alzheimer’s disease; PA: pathological aging; PSP: progressive supranuclear palsy; Other: other types of dementia.

**Supplemental Table S2**: Demographic characteristics of the ROSMAP, MSBB, and Mayo Alzheimer’s disease cohorts.

| **ROSMAP: DLPFC** | **NCI** | **MCI** | **AD** | **Other** |
| --- | --- | --- | --- | --- |
| N | 195 | 161 | 208 | 17 |
| Age (years) (median [IQR]) | 86.11[81.84-90] | 89.42[85.38-90] | 90[87.64-90] | 88.96[85.90-90] |
| Sex=Male (%) | 77(39.49%) | 55(34.16%) | 65(30.29%) | 10(58.82%) |
| Edu (years) (median [IQR]) | 16[13.5-19] | 16[14-18] | 16[14-18] | 18[15-21] |
| APOE genotype | |  |  |  |
| e22 (%) | 2(1.03%) | 1(0.62%) | 1(0.48%) | 0(0%) |
| e23 (%) | 34(17.44%) | 14(8.75%) | 21(10.1%) | 3(17.65%) |
| e24 (%) | 2(1.03%) | 4(2.5%) | 8(3.85%) | 1(5.88%) |
| e33 (%) | 126(64.62%) | 114(71.25%) | 108(51.92%) | 9(52.94%) |
| e34 (%) | 30(15.38%) | 27(16.88%) | 68(32.69%) | 3(17.65%) |
| e44 (%) | 1(0.51%) | 0(0%) | 2(0.96%) | 1(5.88%) |
| Ethnicity |  |  |  |  |
| American Indian or Alaska Native (%) | 0(0%) | 1(0.62%) | 0(0%) | 0(0%) |
| Black (%) | 2(1.03%) | 1(0.62%) | 2(0.96%) | 0(0%) |
| White (%) | 193(98.97%) | 159(98.76%) | 206(99.04%) | 17(100%) |
| MMSE score (median [IQR]) | 28.75[27-29.5] | 26[24-28] | 15[5-20] | 16[11-20] |
| **MSBB: FP** | **NCI** | **AD** |  |  |
| N | 48 | 114 |  |  |
| Age (years) (median [IQR]) | 82[74-89.25] | 86[80-90] |  |  |
| Sex=Male (%) | 23(47.92%) | 39(34.21%) |  |  |
| APOE genotype | |  |  |  |
| e22 (%) | 1(4.17%) | 0(0%) |  |  |
| e23 (%) | 5(20.83%) | 2(2.94%) |  |  |
| e24 (%) | 0(0%) | 1(1.47%) |  |  |
| e33 (%) | 14(58.33%) | 37(54.41%) |  |  |
| e34 (%) | 4(16.67%) | 25(36.76%) |  |  |
| e44 (%) | 0(0%) | 3(4.41%) |  |  |
| Ethnicity |  |  |  |  |
| American Native or Alaskan Native (%) | 1(2.08%) | 0(0%) |  |  |
| Black (%) | 6(12.5%) | 10(8.77%) |  |  |
| Hispanic (%) | 6(12.5%) | 4(3.51%) |  |  |
| White (%) | 35(72.92%) | 99(86.84%) |  |  |
| Unknown (%) | 0(0%) | 1(0.88%) |  |  |
| CDR score (median [IQR]) | 0.25[0-0.5] | 3[2-4] |  |  |
| **MSBB: IFG** | **NCI** | **AD** |  |  |
| N | 34 | 91 |  |  |
| Age (years) (median [IQR]) | 82.5[74.25-90] | 86[80-90] |  |  |
| Sex=Male (%) | 17(50%) | 30(32.97%) |  |  |
| APOE genotype | |  |  |  |
| e22 (%) | 0(0%) | 0(0%) |  |  |
| e23 (%) | 4(30.77%) | 1(1.89%) |  |  |
| e24 (%) | 0(0%) | 1(1.89%) |  |  |
| e33 (%) | 8(61.54%) | 33(62.26%) |  |  |
| e34 (%) | 1(7.69%) | 15(28.3%) |  |  |
| e44 (%) | 0(0%) | 3(5.66%) |  |  |
| Ethnicity |  |  |  |  |
| American Native or Alaskan Native (%) | 0(0%) | 0(0%) |  |  |
| Black (%) | 4(11.76%) | 8(8.79%) |  |  |
| Hispanic (%) | 6(17.65%) | 2(2.2%) |  |  |
| White (%) | 24(70.59%) | 80(87.91%) |  |  |
| Unknown (%) | 0(0%) | 1(1.1%) |  |  |
| CDR score (median [IQR]) | 0.5[0-0.5] | 3[2-4] |  |  |
| **MSBB: STG** | **NCI** | **AD** |  |  |
| N | 34 | 91 |  |  |
| Age (years) (median [IQR]) | 82[74-89] | 85[78.5-90] |  |  |
| Sex=Male (%) | 16(43.24%) | 41(35.65%) |  |  |
| APOE genotype | |  |  |  |
| e22 (%) | 0(0%) | 0(0%) |  |  |
| e23 (%) | 5(27.78%) | 4(6.35%) |  |  |
| e24 (%) | 0(0%) | 1(1.59%) |  |  |
| e33 (%) | 10(55.56%) | 36(57.14%) |  |  |
| e34 (%) | 3(16.67%) | 20(31.75%) |  |  |
| e44 (%) | 0(0%) | 2(3.17%) |  |  |
| Ethnicity |  |  |  |  |
| American Native or Alaskan Native (%) | 0(0%) | 0(0%) |  |  |
| Black (%) | 6(16.22%) | 13(11.3%) |  |  |
| Hispanic (%) | 6(16.22%) | 3(2.61%) |  |  |
| White (%) | 25(67.57%) | 98(85.22%) |  |  |
| Unknown (%) | 0(0%) | 1(0.87%) |  |  |
| CDR score (median [IQR]) | 0[0-0.5] | 3[3-5] |  |  |
| **MSBB: PHG** | **NCI** | **AD** |  |  |
| N | 49 | 110 |  |  |
| Age (years) (median [IQR]) | 79[74-89] | 85[80-90] |  |  |
| Sex=Male (%) | 24(48.98%) | 40(36.36%) |  |  |
| APOE genotype | |  |  |  |
| e22 (%) | 1(3.57%) | 0(0%) |  |  |
| e23 (%) | 7(25%) | 3(5.88%) |  |  |
| e24 (%) | 0(0%) | 1(1.96%) |  |  |
| e33 (%) | 16(57.14%) | 30(58.82%) |  |  |
| e34 (%) | 4(14.29%) | 14(27.45%) |  |  |
| e44 (%) | 0(0%) | 3(5.88%) |  |  |
| Ethnicity |  |  |  |  |
| American Native or Alaskan Native (%) | 1(2.04%) | 0(0%) |  |  |
| Black (%) | 8(16.33%) | 11(10%) |  |  |
| Hispanic (%) | 8(16.33%) | 2(1.82%) |  |  |
| White (%) | 32(65.31%) | 95(86.36%) |  |  |
| Unknown (%) | 0(0%) | 2(1.82%) |  |  |
| CDR score (median [IQR]) | 0[0-0.5] | 3[2-5] |  |  |
| **Mayo: TCX** | **NCI** | **AD** | **PA** | **PSP** |
| N | 78 | 82 | 30 | 84 |
| Age (years) (median [IQR]) | 86[79-89.75] | 85[78.25-89] | 86.5[83.25-88] | 74[69-79] |
| Sex=Male (%) | 41(52.56%) | 33(40.24%) | 13(43.33%) | 51(60.71%) |
| APOE genotype | |  |  |  |
| e22 (%) | 0(0%) | 0(0%) | 1(3.33%) | 0(0%) |
| e23 (%) | 12(15.38%) | 4(4.88%) | 3(10%) | 15(17.86%) |
| e24 (%) | 1(1.28%) | 0(0%) | 0(0%) | 1(1.19%) |
| e33 (%) | 57(73.08%) | 35(42.68%) | 16(53.33%) | 56(66.67%) |
| e34 (%) | 8(10.26%) | 36(43.9%) | 10(33.33%) | 11(13.1%) |
| e44 (%) | 0(0%) | 7(8.54%) | 0(0%) | 1(1.19%) |
| Ethnicity |  |  |  |  |
| White (%) | 78 (%) | 82(%) | 30(%) | 84(%) |
| **Mayo: CER** | **NCI** | **AD** | **PA** | **PSP** |
| N | 77 | 82 | 28 | 84 |
| Age (years) (median [IQR]) | 85[79-89] | 84.5[78.25-89] | 86[82.25-88] | 74[69-79] |
| Sex=Male (%) | 40(51.95%) | 34(41.46%) | 12(42.86%) | 51(60.71%) |
| APOE genotype | |  |  |  |
| e22 (%) | 0(0%) | 0(0%) | 1(3.57%) | 0(0%) |
| e23 (%) | 10(12.99%) | 4(4.88%) | 3(10.71%) | 14(16.67%) |
| e24 (%) | 1(1.3%) | 0(0%) | 0(0%) | 1(1.19%) |
| e33 (%) | 57(74.03%) | 35(42.68%) | 15(53.57%) | 57(67.86%) |
| e34 (%) | 9(11.69%) | 38(46.34%) | 9(32.14%) | 11(13.1%) |
| e44 (%) | 0(0%) | 5(6.1%) | 0(0%) | 1(1.19%) |
| Ethnicity |  |  |  |  |
| White (%) | 77 (100%) | 82 (100%) | 28 (100%) | 84 (100%) |

Notes: Participants aged >90 were truncated at age 90 in this table. DLPFC dorsolateral prefrontal cortex, FP frontal pole, IFG inferior frontal gyrus, STG superior temporal gyrus, PHG parahippocampal gyrus, TCX temporal cortex, CER cerebellum. NCI: no cognitive impairment; MCI: mild cognitive impairment; AD: Alzheimer’s disease; PA: pathological aging; PSP: progressive supranuclear palsy; Other: other types of dementia.

**Supplemental Table S3**: qPCR primer information.

| Gene Name | Sequence |
| --- | --- |
| B4GALT1 | FW: CTATATCTCGCCCAAATGCTG  RV: GTGCAATTCGGTCAAACCTC |
| B3GALT5 | FW: ATCAGGCAGCCATTCAGCAA  RV: ACGTCGCCAGAAAACACGTA |
| GALNT15 | FW: TGTCCTGAGGGTGCTGCT  RV: AAGAGGAGTTGACCCCGTTC |
| GALNT11 | FW: TGTCCAGGTCGCAGATGTAA  RV: GTCGGACTGGGGCTGTG |
| GALNT10 | FW: GCCCATCTCCCAAGAAAAA  RV: GCTTTGGGCGCTGTACC |
| b-Actin | FW: TCAAGATCATTGCTCCTCCTGAG  RV: ACATCTGCTGGAAGGTGGACA |

| Gene Name | Unique Assay ID |
| --- | --- |
| ST6GALNAC5 | qHsaCID0007142 |
| ST3GAL3 | qHsaCED0036502 |
| ALG1 | qHsaCID0016546 |
| ST6GALNAC3 | qHsaCID0036897 |
| ST3GAL5 | qHsaCID0013823 |
| ST6GALNAC2 | qHsaCID0016785 |
| GALNTL6 | qHsaCED0057234 |
| B3GALT2 | qHsaCID0023450 |
| NEU3 | qHsaCED0045967 |
| ST6GAL2 | qHsaCID0007061 |
| UGT8 | qHsaCID0017816 |
| B4GALT6 | qHsaCID0015245 |
| B3GALNT1 | qHsaCED0046621 |
| A4GALT | qHsaCED0002403 |
| PIGM | qHsaCED0018886 |
| B4GALT5 | qHsaCID0015112 |

**Supplemental Table S4**: Demographic data of participants in the glycomic analysis.

|  | LCBC | | LPFC | | MTC | |
| --- | --- | --- | --- | --- | --- | --- |
|  | AD | Control | AD | Control | AD | Control |
| n | 7 | 10 | 10 | 9 | 9 | 6 |
| Age (years) (median [IQR]) | 80[77.5-86] | 85.5[79.75-90] | 81[77.25-90] | 87[79-90] | 80[77-90] | 88.5[84.75-90] |
| Sex=Male (%) | 2(29) | 3(30) | 3(30) | 3(33) | 3(33) | 2(33) |
| Ethnicity | | | | | | |
| Black (%) | 3(43) | 1(10) | 3(30) | 1(11) | 3(33) | 1(17) |
| White (%) | 4(57) | 9(90) | 7(70) | 8(89) | 6(67) | 5(83) |

Notes: LCBC lateral cerebellum cortex, LPFC lateral prefrontal cortex, MTC medial temporal cortex. Participants aged >90 were truncated at age 90 in this table.

**Supplemental Table S5**: Glycosyltransferases excluded for low expression.

| DDLPFC | MGAT2/MGAT4D/GALNT4/GALNT5/LARGE2/B3GNT3/A4GNT/B4GALNT2/FUT5/FUT6/CHST4/HS3ST6/NDST4/NEU2 |
| --- | --- |
| FP | MGAT4D/GALNT5/B3GNT6/LARGE2/B3GNT3/A4GNT/B4GALNT2/FUT3/FUT5/FUT6/FUT7/CHST13/HS3ST6/NEU2 |
| STG | MGAT2/MGAT4D/GCNT3/B3GNT6/LARGE2/B3GNT3/A4GNT/B4GALNT2/FUT3/FUT5/FUT6/CHST13/HS3ST3A1/HS3ST6/NEU2 |
| PHG | MGAT4D/GALNT4/GALNT5/GCNT3/B3GNT6/LARGE2/B3GNT3/A4GNT/B4GALNT2/FUT3/FUT5/FUT6/ST8SIA2/CHST13/HS3ST3A1/HS3ST6/NEU2 |
| IFG | MGAT4D/GALNT5/GCNT3/B3GNT6/LARGE2/B3GNT3/A4GNT/B4GALNT2/FUT3/FUT5/FUT6/CHST13/HS3ST6/NEU2 |
| CER | MGAT2/MGAT4D/GALNT4/GALNTL5 (T20)/B3GNT6/B3GNT3/A4GNT/B4GALNT2/FUT5/FUT6/CHST13/CHST4/HS3ST3A1/HS3ST6/NDST4/NEU2 |
| TCX | MGAT2/MGAT4D/GALNT5/B3GNT6/LARGE2/B3GNT3/A4GNT/B4GALNT2/FUT5/FUT6/CHST4/HS3ST6/NEU2 |

Note: The gene expression cutoff is a minimum CPM of 10 in at least 70% of samples.

**Supplemental Table S6**: Potential transcription factors and their target genes.

See the PDF file: SupplementaryTableS6TF_motif.pdf.

geneSet: The glycosylation pathway to which the glycosyltransferes belong.

Motif: ID of the motif.

NES: Normalized enrichment score of the motif in the gene-set. It was calculated based on the AUC distribution of all the motifs for the gene-set [(x-mean)/sd].

AUC: area under the curve.

TF.highConf: Transcription factors with high confident.

nEnrGenes: The number of genes that are highly ranked for the given motif.

enrichedGenes: hgnc_symbol of genes that are highly ranked for the given motif.

**Supplemental Table S7**: miRNAs targeting the genes of glycosyltransferases according to validated databases (miRecords, miRTarBase and TarBase).

See the excel file: SupplementaryTableS7miRNA-targets.xlsx.

A: mature miRNAs targeting glycosyltransferases (GTs) for lipid glycosylation

B: mature miRNAs targeting glycosyltransferases (GTs) for N-glycosylation

C: mature miRNAs targeting glycosyltransferases (GTs) for mucin O-glycosylation

D: mature miRNAs targeting glycosyltransferases (GTs) for non-mucin O-glycosylation

E: mature miRNAs targeting glycosyltransferases (GTs) for Secretory C-mannosylation

F: mature miRNAs targeting glycosyltransferases (GTs) for elongation and branching

G: mature miRNAs targeting glycosyltransferases (GTs) for capping

H: mature miRNAs targeting glycosyltransferases (GTs) for sulfation

I: mature miRNAs targeting glycosyltransferases (GTs) for hydrolases

# **Supplementary Figures**

**
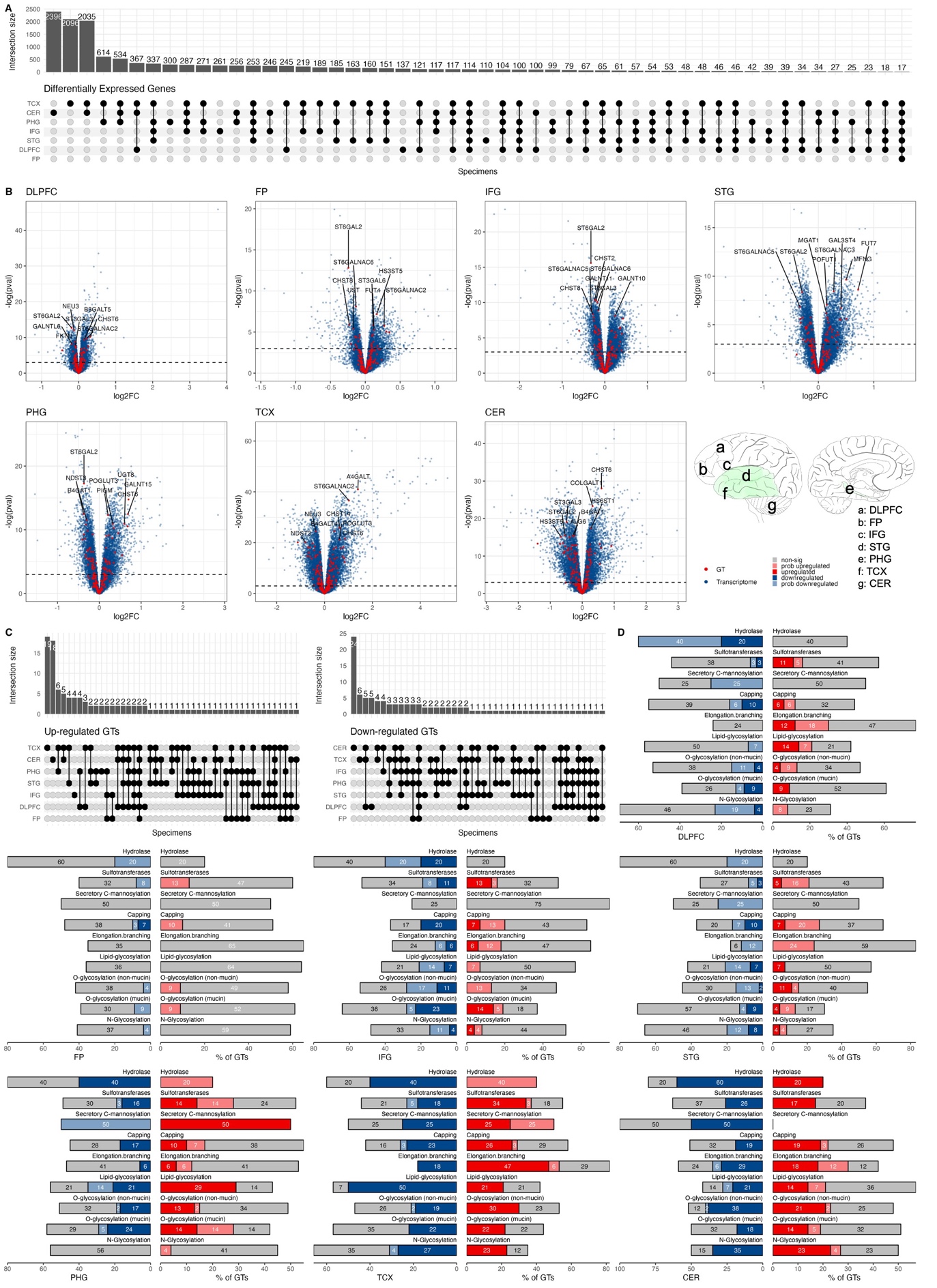
**

**Supplemental Figure S1:** The overview of differentially expressed genes (DEGs) and glycosyltransferases (GTs) in AD vs. NCI using public RNA-seq data from MSBB, Mayo, and ROSMAP. A) The upset plot showing the number of DEGs overlapped between multiple regions (adjusted p-value < 0.05). B) Volcano plots of seven brain regions showing the distribution of genes and the magnitude of the differences. Red dots, glycosyltransferases (GTs); blue dots: protein-coding genes; the dashed line shows the significance cutoff (0.05). GTs with the lowest p-values were marked in the volcano plots. C) Upset plots showing the number of up-/down-regulated GTs overlapped between multiple regions (unadjusted p-value <0.05). The color showed the number of differentially expressed GTs. D) The percentage of GTs in each glycosylation pathway affect by AD in each brain region. Dark red/blue bars: GTs up-/down-regulated in AD vs. control with adjusted p-values<0.05; light red/blue bars: GTs probably upregulated in AD vs. control with p-values<0.05 but adjusted p-values>0.05; grey bars: GTs that didn’t significantly changed in AD vs. control (p-values>0.05). Figures generated using R 4.1.0 (R Foundation for Statistical Computing, Vienna, Austria).


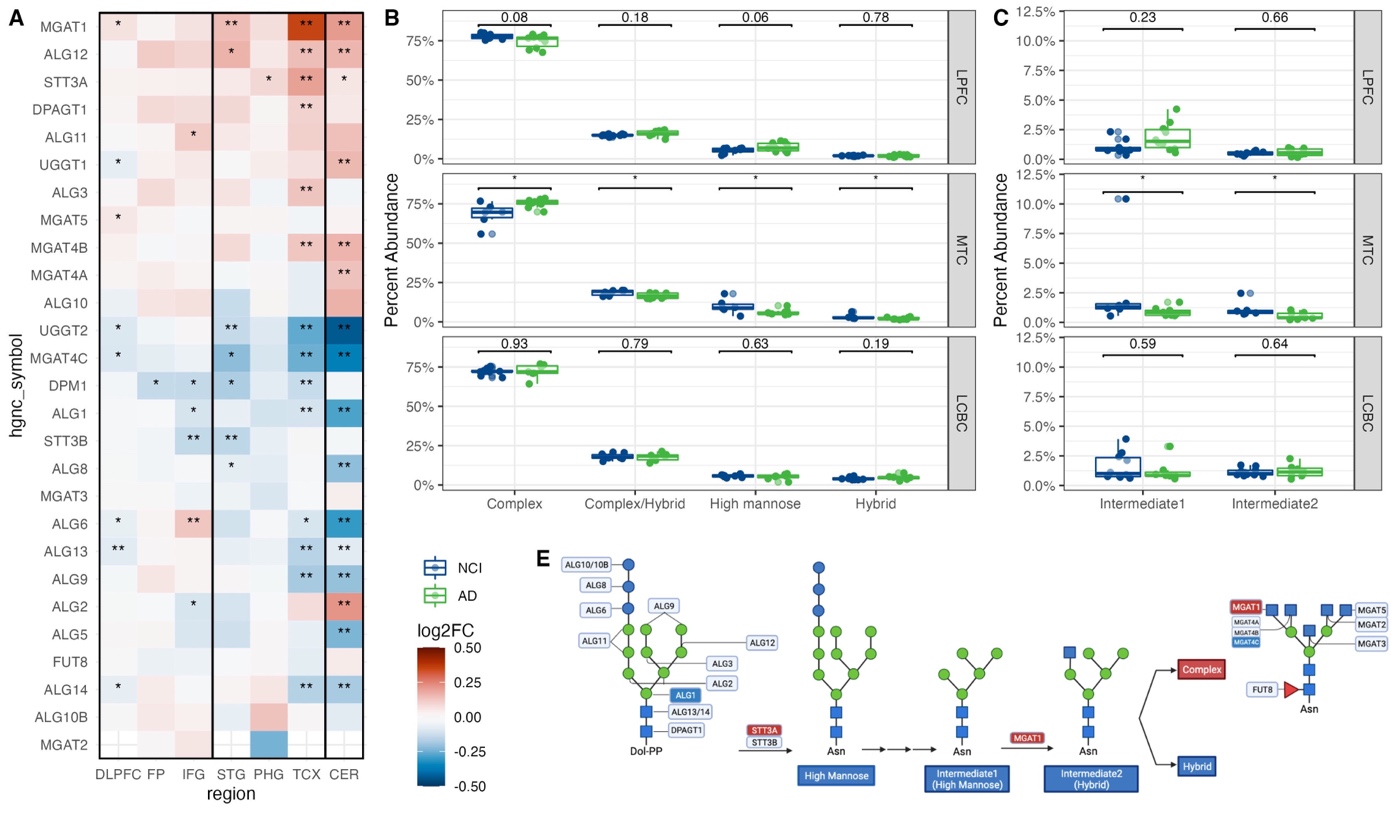


**Supplemental Figure S2**: Differential expression of N-glycan genes and altered N-glycan abundances in brain specimens. A) The heatmap showed the glycosyltransferases (GTs) differential expression specific to N-glycosylation. Red/blue: up-/down-regulated in AD vs. NCI. * p-value <0.05 but adjusted p-value>0.05, ** p-value<0.05 and adjusted p-value<0.05. adjusted p-value were calculated based on all protein-coding genes. B) The abundance of N-glycan subtypes from N-glycomics in three brain regions of AD and control participants. C) The abundance of two intermediate structures (Hex:5 HexNAc: 2, Hex:5 HexNAc: 3) from N-glycomics in the three brain regions of AD and control participants. *p-value<0.05. LPFC: lateral prefrontal cortex; MTC: medial temporal cortex; LCBC: lateral cerebellar cortex. D) A schematic of the N-glycosylation process. Glycan symbol key: green circles, mannose (Man); blue squares, N-acetylglucosamine (GlcNAc); red triangles, fucose (Fuc). Figures were generated using R 4.1.0 (R Foundation for Statistical Computing, Vienna, Austria). The schematic diagram of glycans were created with BioRender.com.


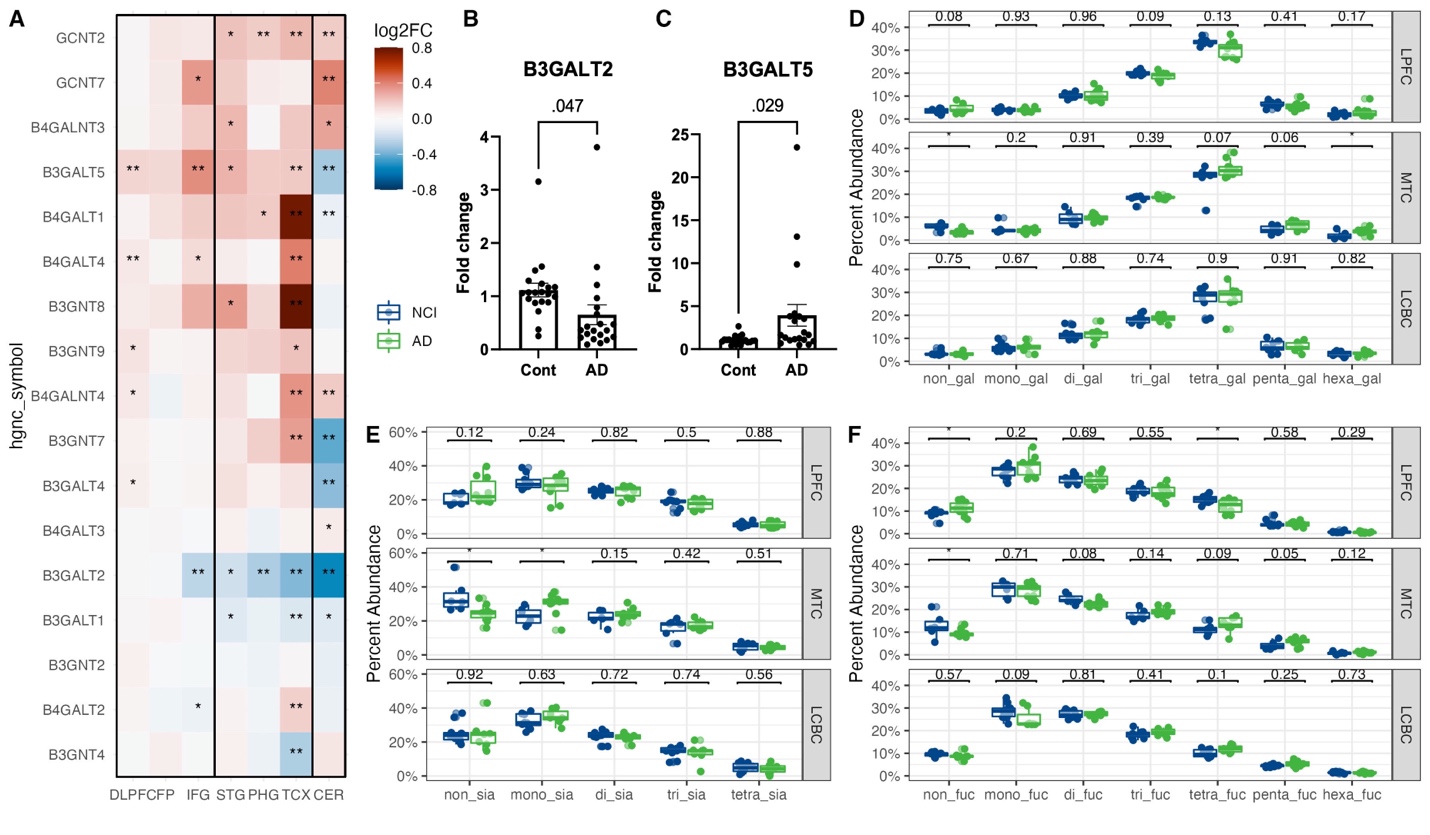


**Supplemental Figure S3**: Differential expression of elongation genes and altered N-glycans abundances in brain specimens. A) The heatmap showed the glycosyltransferases (GTs) differential expression for elongation. Red/blue: up-/down-regulated in AD vs. NCI. * p-value <0.05 but adjusted p-value>0.05, ** p-value<0.05 and adjusted p-value<0.05. Adjusted p-values were calculated based on all protein-coding genes. B-C) The qPCR confirmation of B3GALT2 (B) and B3GALT5 (C). D) The abundance of N-glycans with different numbers of galactoses from N-glycomics in three brain regions of AD and control participants. E-F) The abundance of N-glycans with different numbers of Neu5Acs (E) and fucoses (F) from N-glycomics in three brain regions of AD and control participants. *p-value<0.05. LPFC: lateral prefrontal cortex; MTC: medial temporal cortex; LCBC: lateral cerebellar cortex. Figures were generated using R 4.1.0 (R Foundation for Statistical Computing, Vienna, Austria).


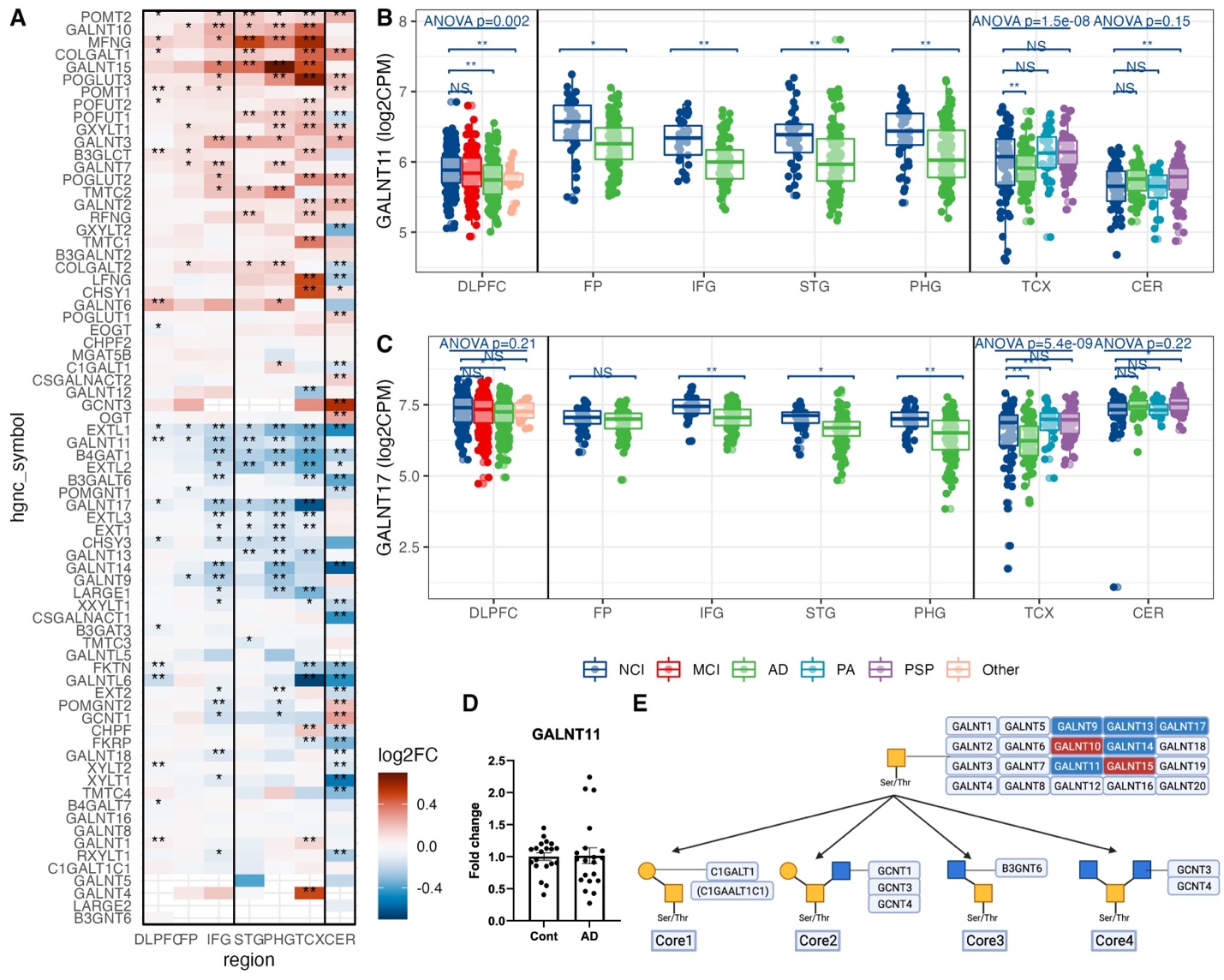


**Supplemental Figure S4**: Differential expression of O-glycosylation genes in brain specimens. A) The heatmap showed the glycosyltransferases (GTs) differential expression for O-glycosylation. Red/blue: up-/down-regulated in AD vs. NCI. * p-value <0.05 but adjusted p-value>0.05, ** p-value<0.05 and adjusted p-value<0.05. Adjusted p-values were calculated based on all protein-coding genes. B-C) The changes of GALNT11 (B) and GALNT17 (C) across human brains with different diagnoses. Data presented as log2 count per million (log2CPM). Data from different studies were separated into blocks. Adjusted p-value were calculated based on all protein-coding genes. NS p-value >0.05, * p-value <0.05 but adjusted p-value>0.05, ** p-value<0.05 and adjusted p-value<0.05. ANOVA p-values were after the multiple testing correction over all protein-coding genes. DLPFC dorsolateral prefrontal cortex, FP frontal pole, IFG inferior frontal gyrus, STG superior temporal gyrus, PHG parahippocampal gyrus, TCX temporal cortex, CER cerebellum. NCI: no cognitive impairment; MCI: mild cognitive impairment; AD: Alzheimer's disease; PA: pathological aging; PSP: progressive supranuclear palsy; Other: other types of dementia. D) qPCR results of GALNT11 expression in MTC of AD and control participants. E) A schematic of the O-GalNAc-glycosylation process. Glycan symbol key: yellow circles, galactose (Gal); yellow squares, N-acetylgalactosamine (GalNAc); blue squares, N-acetylglucosamine (GlcNAc). Figures were generated using R 4.1.0 (R Foundation for Statistical Computing, Vienna, Austria). The schematic diagram of glycans were created with BioRender.com.


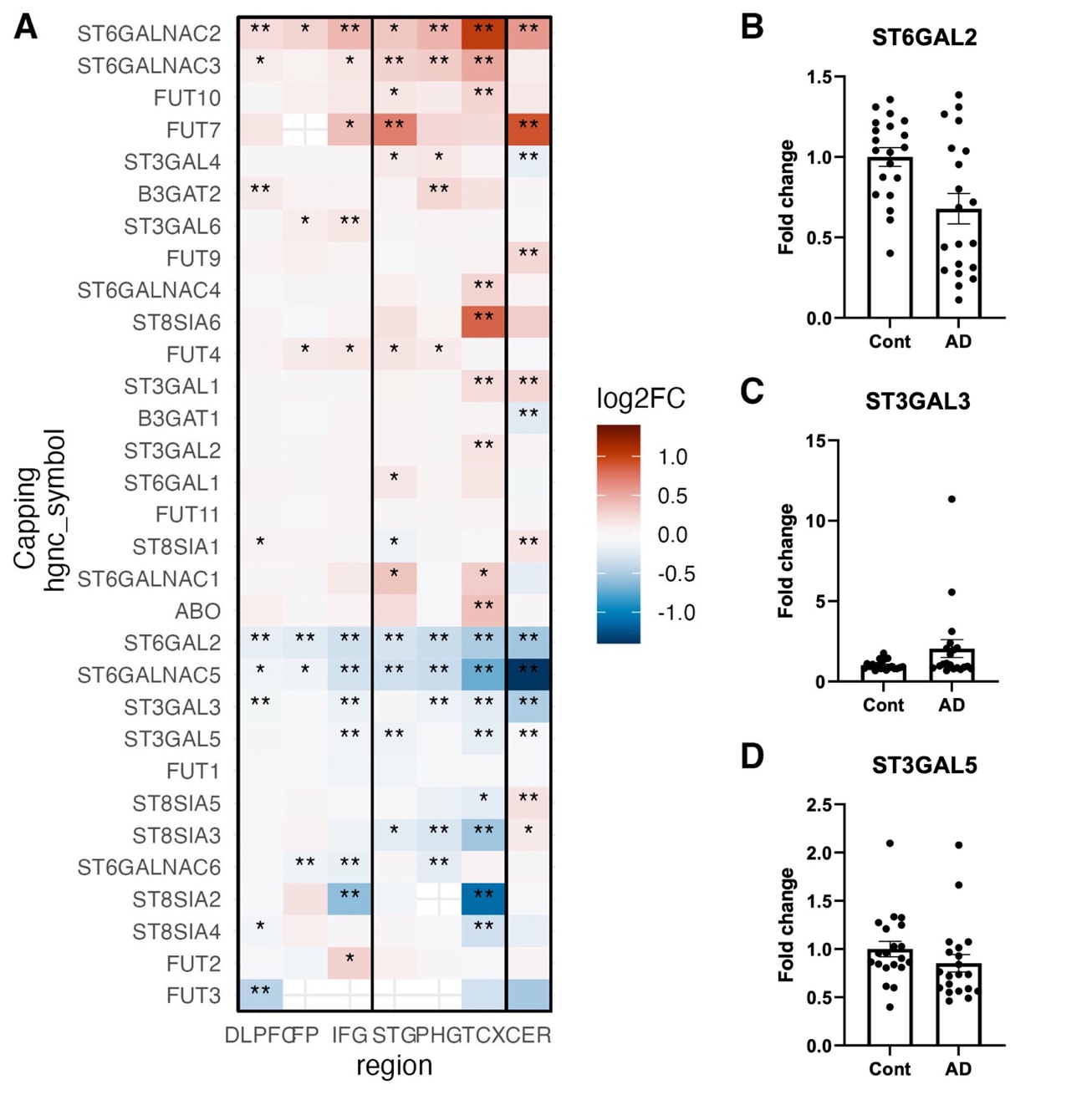


**Supplemental Figure S5**: Differential expression of capping genes in brain specimens. A) The heatmap showed the glycosyltransferases (GTs) differential expression for capping. Red/blue: up-/down-regulated in AD vs. NCI. * p-value <0.05 but adjusted p-value>0.05, ** p-value<0.05 and adjusted p-value<0.05. Adjusted p-values were calculated based on all protein-coding genes. B-D) qPCR results of ST6GAL2 (B), ST3GAL3 (C), and ST3GAL5 (D) expression in MTC of AD and control participants. Figures were generated using R 4.1.0 (R Foundation for Statistical Computing, Vienna, Austria).


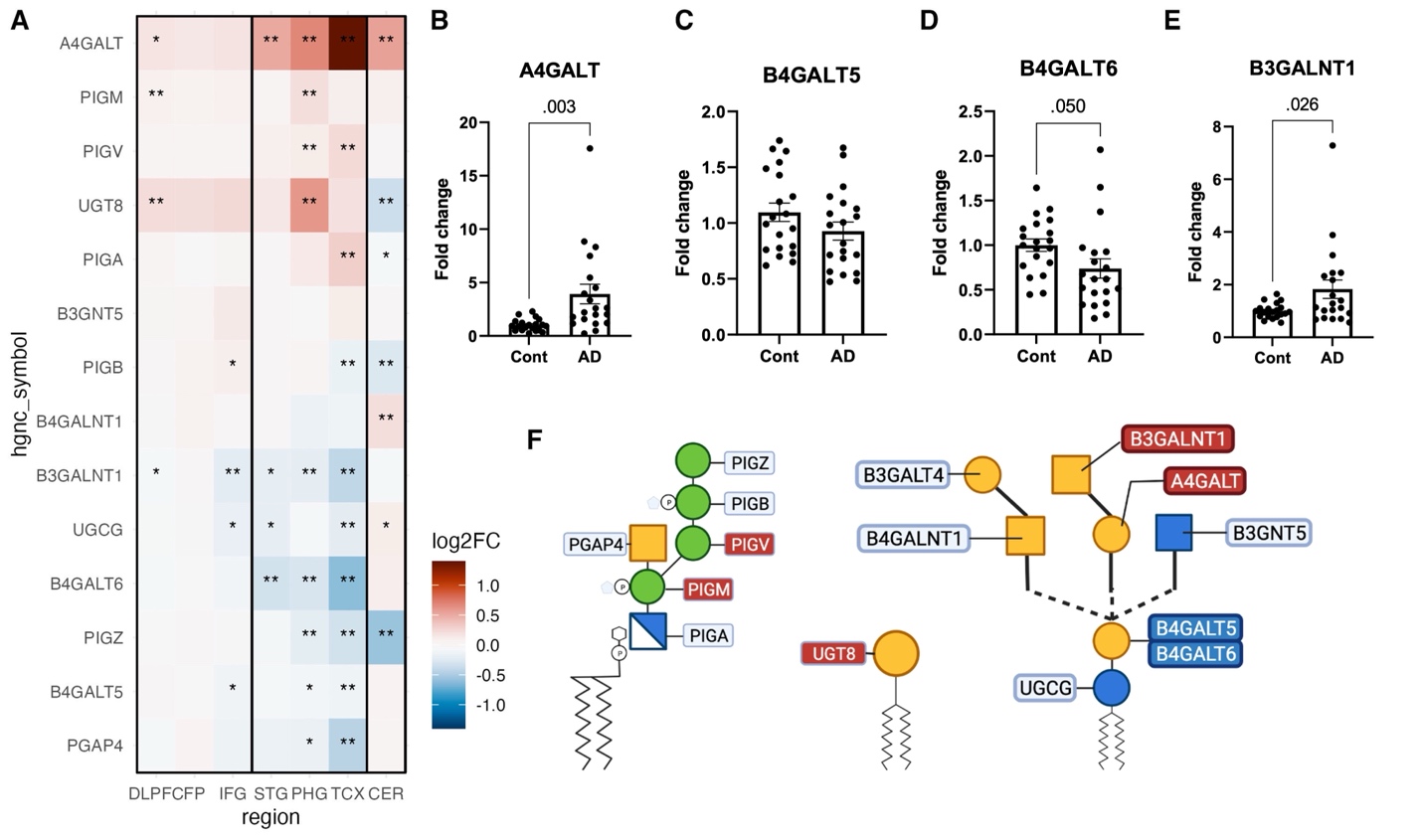


**Supplemental Figure S6**: Differential expression of lipid glycosylation genes in brain specimens. A) The heatmap showed the glycosyltransferases (GTs) differential expression for lipid glycosylation. Red/blue: up-/down-regulated in AD vs. NCI. * p-value <0.05 but adjusted p-value>0.05, ** p-value<0.05 and adjusted p-value<0.05. Adjusted p-values were calculated based on all protein-coding genes. B-E) qPCR results of A4GALT (B), B4GALT5 (C), B4GALT6 (D), and B3GALNT1 (E) expression in MTC of AD and control participants. F) A schematic of the GPI-anchor, galactosylceramides, and glycosphingolipids biosynthesis. Glycan symbol key: yellow circles, galactose (Gal); yellow squares, N-acetylgalactosamine (GalNAc); blue circles, N-glucose (Glc); blue squares, N-acetylglucosamine (GlcNAc); green circles, mannose (Man); blue cross squares, glucosamine (GlcN). Figures were generated using R 4.1.0 (R Foundation for Statistical Computing, Vienna, Austria). The schematic diagram of glycans were created with BioRender.com.


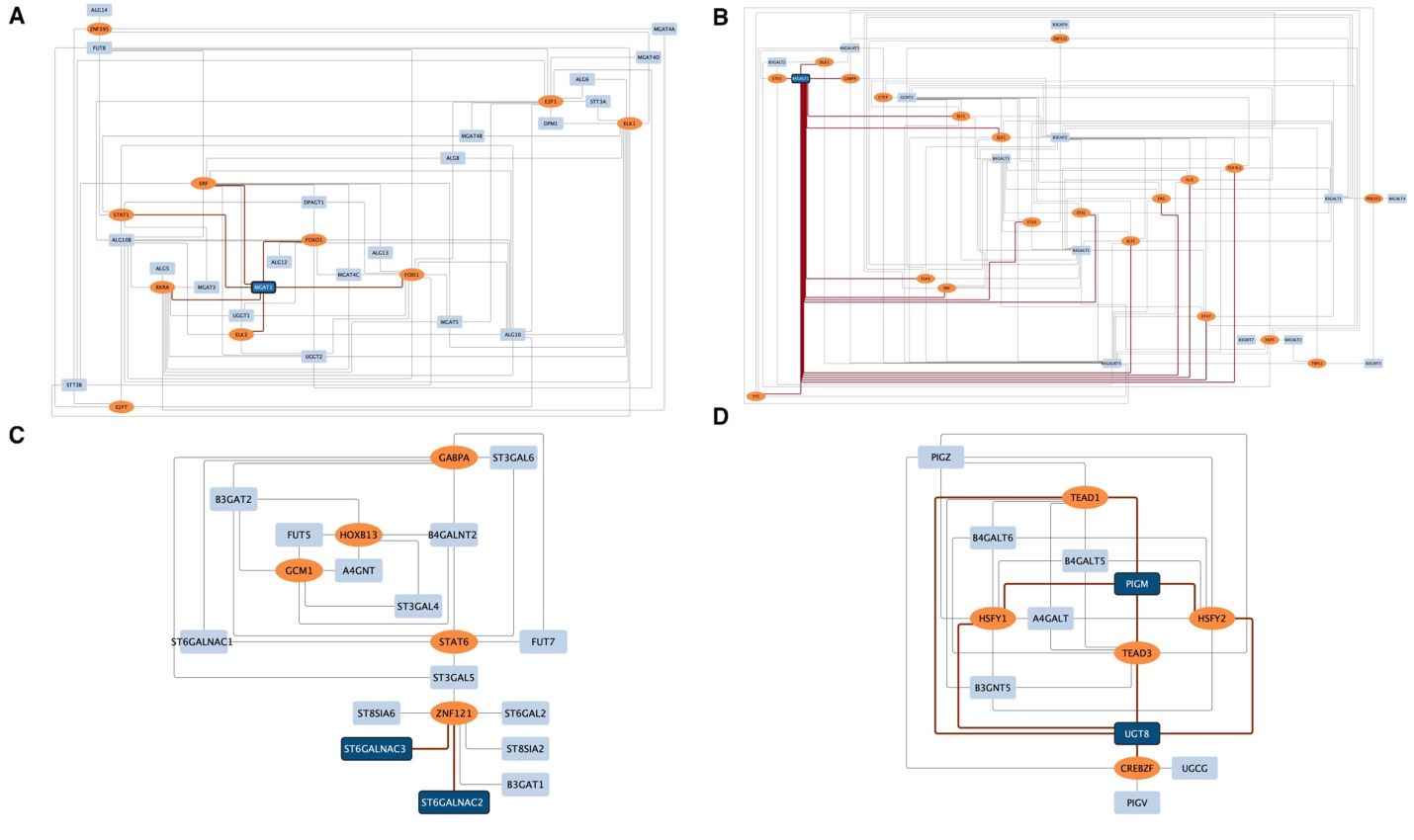


**Supplemental Figure S7**: Predicted transcription factors (TF) responsible for glycosylation. Regulatory networks for A) N-glycan core structure biosynthesis, B) elongation and branching, C) capping, and D) glycolipid biosynthesis. TFs with normalized enrichment score (NES) > 4.5 were shown in the network. TFs were represented as orange ellipse; glycosyltransferases were represented as blue rectangles. The significant glycosyltransferases were highlighted with in a dark shade of blue. The brown edges showed interactions between significant genes and TFs. Figures were generated using R 4.1.0 (R Foundation for Statistical Computing, Vienna, Austria).


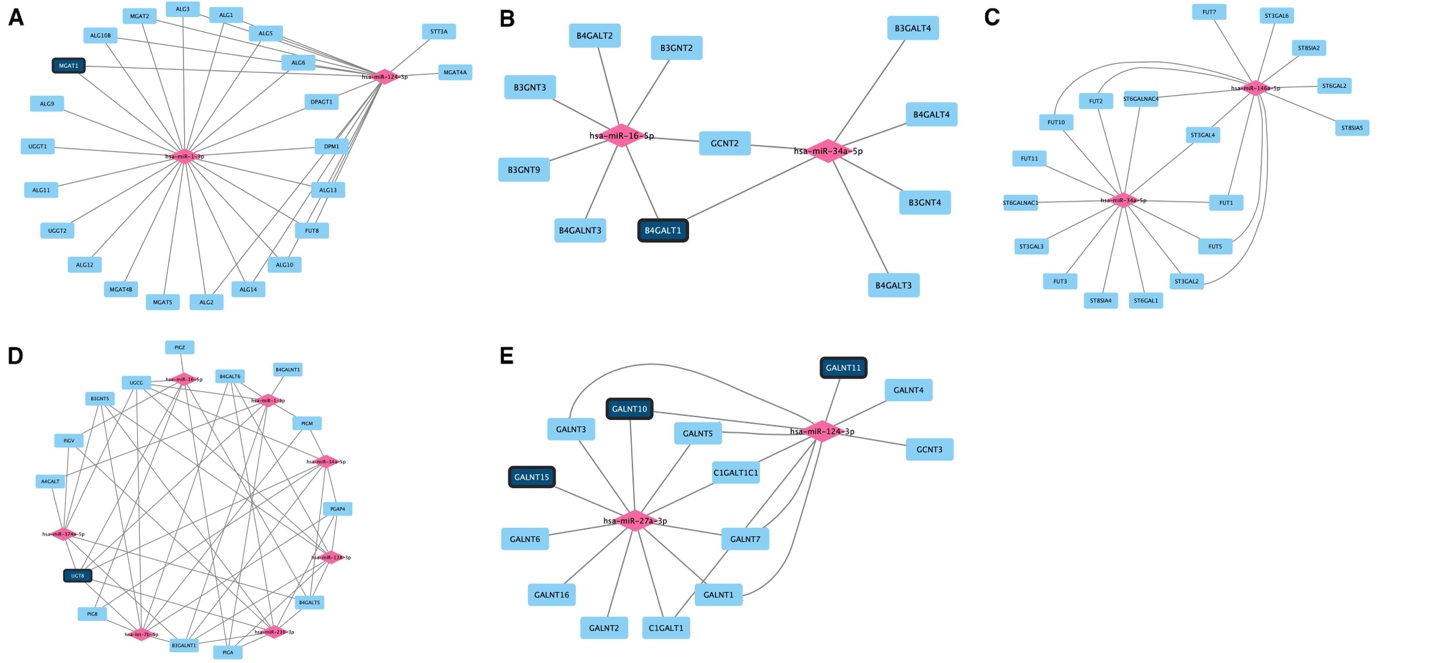


**Supplemental Figure S8**: Top 2 miRNA regulated the most number of glycosyltransferases and their validated targets. Regulatory networks for A) N-glycan core structure biosynthesis, B) elongation and branching, C) capping, and D) glycolipid biosynthesis, and E) O-GalNAc-glycan core structure biosynthesis. miRNAs were represented as purple diamonds; glycosyltransferases were represented as blue rectangles. The significant glycosyltransferases were highlighted with in a dark shade of blue. The brown edges showed interactions between significant genes and miRNAs. Figures were generated using R 4.1.0 (R Foundation for Statistical Computing, Vienna, Austria).


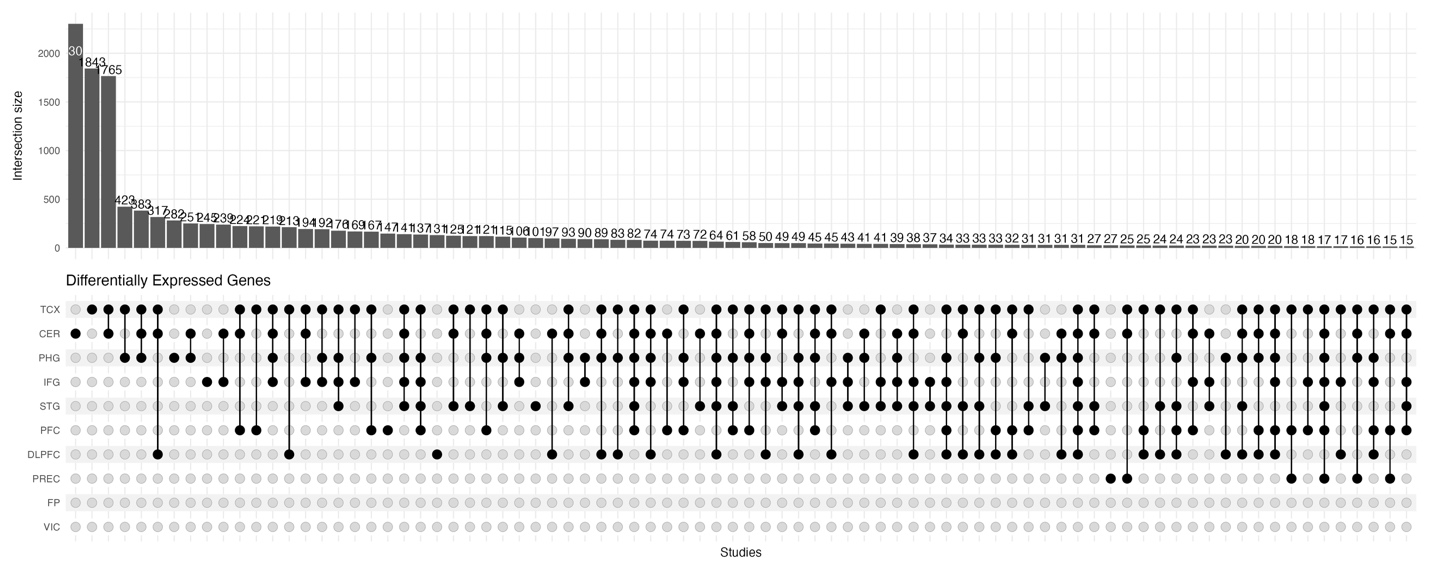


**Supplemental Figure S9**: The upset plot showing the number of DEGs overlapped between our study (adjusted p-value < 0.05) and other publications [10,11]. Figures were generated using R 4.1.0 (R Foundation for Statistical Computing, Vienna, Austria).

# **References**

[1] Wang M, Beckmann ND, Roussos P, Wang E, Zhou X, Wang Q, et al. The Mount Sinai cohort of large-scale genomic, transcriptomic and proteomic data in Alzheimer’s disease. Sci Data 2018;5. https://doi.org/10.1038/sdata.2018.185.

[2] Wan YW, Al-Ouran R, Mangleburg CG, Perumal TM, Lee T V., Allison K, et al. Meta-Analysis of the Alzheimer’s Disease Human Brain Transcriptome and Functional Dissection in Mouse Models. Cell Rep 2020;32. https://doi.org/10.1016/j.celrep.2020.107908.

[3] De Jager PL, Ma Y, McCabe C, Xu J, Vardarajan BN, Felsky D, et al. Data descriptor: A multi-omic atlas of the human frontal cortex for aging and Alzheimer’s disease research. Sci Data 2018;5. https://doi.org/10.1038/sdata.2018.142.

[4] Allen M, Carrasquillo MM, Funk C, Heavner BD, Zou F, Younkin CS, et al. Human whole genome genotype and transcriptome data for Alzheimer’s and other neurodegenerative diseases. Sci Data 2016;3. https://doi.org/10.1038/sdata.2016.89.

[5] McCarthy DJ, Chen Y, Smyth GK. Differential expression analysis of multifactor RNA-Seq experiments with respect to biological variation. Nucleic Acids Res 2012;40:4288–97. https://doi.org/10.1093/nar/gks042.

[6] Montine TJ, Phelps CH, Beach TG, Bigio EH, Cairns NJ, Dickson DW, et al. National institute on aging-Alzheimer’s association guidelines for the neuropathologic assessment of Alzheimer’s disease: A practical approach. Acta Neuropathol 2012;123:1–11. https://doi.org/10.1007/s00401-011-0910-3.

[7] Aibar S, González-Blas CB, Moerman T, Huynh-Thu VA, Imrichova H, Hulselmans G, et al. SCENIC: Single-cell regulatory network inference and clustering. Nat Methods 2017;14:1083–6. https://doi.org/10.1038/nmeth.4463.

[8] Ru Y, Kechris KJ, Tabakoff B, Hoffman P, Radcliffe RA, Bowler R, et al. The multiMiR R package and database: Integration of microRNA-target interactions along with their disease and drug associations. Nucleic Acids Res 2014;42. https://doi.org/10.1093/nar/gku631.

[9] Kuznetsova A, Brockhoff PB, Christensen RHB. lmerTest Package: Tests in Linear Mixed Effects Models. J Stat Softw 2017;82:1–26. https://doi.org/10.18637/JSS.V082.I13.

[10] Guennewig B, Lim J, Marshall L, McCorkindale AN, Paasila PJ, Patrick E, et al. Defining early changes in Alzheimer’s disease from RNA sequencing of brain regions differentially affected by pathology. Sci Rep 2021;11. https://doi.org/10.1038/s41598-021-83872-z.

[11] Williams JB, Cao Q, Yan Z. Transcriptomic analysis of human brains with Alzheimer’s disease reveals the altered expression of synaptic genes linked to cognitive deficits. Brain Commun 2021;3. https://doi.org/10.1093/braincomms/fcab123.
