# Supplementary material for: Transcriptomic and glycomic analyses highlight pathway-specific glycosylation alterations unique to Alzheimer’s disease
Source: Sci Rep. 2023 May 15;13:7816. doi: 10.1038/s41598-023-34787-4 (PMC10185676; doi:10.1038/s41598-023-34787-4)
Supplement: Supplementary file 2 — Supplementary Information 2. [file 41598_2023_34787_MOESM2_ESM.pdf]

Supplemental Table S6: Potential transcription factors and their target genes.

logo	geneSet	motif	NES	AUC	TF.highConf	nEnrGenes	enrichedGenes
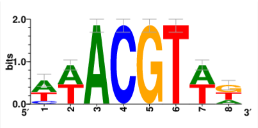	Lipid-glycosylation	cisbp.M0181	4.68	0.2880	CREBZF (inferredBy.Ort	4	PIGV; PIGZ; UGCG; UGT8
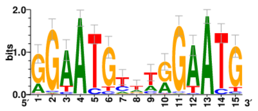	Lipid-glycosylation	metacluster.171.1	4.58	0.2830	HSFY1; HSFY2; TEAD1; TEAD1; TEAD3 (directAnnotation).	7	A4GALT; B3GNT5; B4GALT5; B4GALT6; PIGM; PIGZ; UGT8
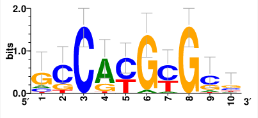	Lipid-glycosylation	transfac.pro.M00322	4.41	0.2750	MAX; MYC (directAnnotation).	5	B3GALNT1; B3GNT5; B4GALT6; UGCG; UGT8
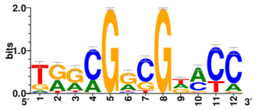	Lipid-glycosylation	transfac.pro.M05432	4.20	0.2640	ZSCAN18 (directAnnotation).	7	B3GALNT1; B3GNT5; B4GALT5; B4GALT6; PIGV; UGCG; UGT8

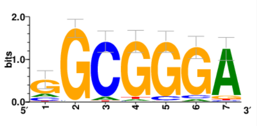	Lipid-glycosylation	metacluster.15.2	4.16	0.2620	E2F1; E2F1; E2F1; E2F1; E2F1; E2F3; E2F4; E2F4; E2F4; E2F6; E2F6; E2F6; E2F6; E2F6; E2F6; E2F6; E2F7; TFDP1; TFDP2; ZNF566; ZNF566; ZNF574 (directAnnotation). E2F3; E2F3; E2F4 (inferredBy.Ort[illegible]	5	B3GNT5; B4GALT5; B4GALT6; UGCG; UGT8
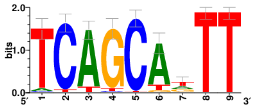	Lipid-glycosylation	cisbp.M08186	4.10	0.2590	BACH1 (directAnnotation).	5	B3GALNT1; B3GNT5; B4GALNT1; PIGV; UGT8
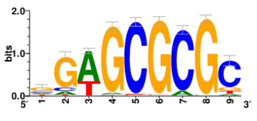	Lipid-glycosylation	hocomoco.ZI[illegible] HUMAN.H11M[illegible]	4.04	0.2560	ZBTB14 (directAnnotation).	10	A4GALT; B3GALNT1; B3GNT5; B4GALNT1; B4GALT6; PIGA; PIGM; PIGZ; UGCG; UGT8

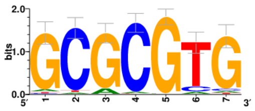	Lipid-glycosylation	metacluster. 198.5	4.02	0.2550	TCFL5; TCFL5 (directAnnotation).	3	B3GALNT1; UGCG; UGT8
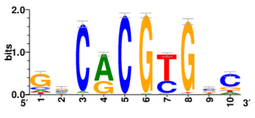	Lipid-glycosylation	taipale. tf. pairs.HES1. GN-CACGT-GNC. HT	4.00	0.2540	HES1 (directAnnotation).	4	B3GALNT1; B4GALT6; UGCG; UGT8
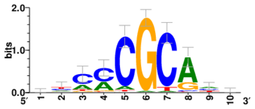	Lipid-glycosylation	cisbp.M00775	3.95	0.2510	ZBTB1 (inferredBy.Orthology).	4	B3GNT5; B4GALT6; UGCG; UGT8
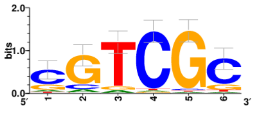	Lipid-glycosylation	metacluster. 67.2	3.94	0.2510	ZNF501 (directAnnotation). ACAA1; E2F2; E2F2; E2F3; E2F3; FOXN4 (inferredBy.Ort[illegible]	4	B3GNT5; B4GALT6; UGCG; UGT8
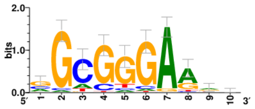	Lipid-glycosylation	homer.GGCGGGAARN E2F6	3.93	0.2500	E2F6 (directAnnotation).	5	B3GNT5; B4GALT5; B4GALT6; UGCG; UGT8
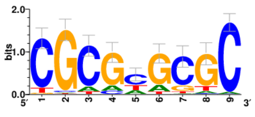	Lipid-glycosylation	transfac. pro.M04866	3.90	0.2490	HDAC2 (directAnnotation).	4	B3GALNT1; B4GALT6; UGCG; UGT8

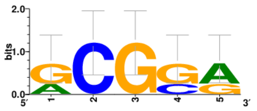	Lipid-glycosylation	hdpi.NRL	3.81	0.2440	NRL (directAnnotation).	4	B3GNT5; B4GALT6; UGCG; UGT8
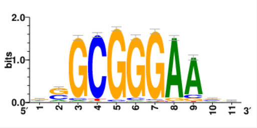	Lipid-glycosylation	jaspar.MA11	3.78	0.2430	TFDP1 (directAnnotation).	10	B3GALNT1; B3GNT5; B4GALNT1; B4GALT5; B4GALT6; PIGM; PIGV; PIGZ; UGCG; UGT8
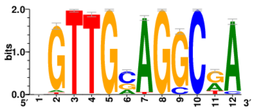	Lipid-glycosylation	transfac.pro.M05505	3.76	0.2420	ZNF2 (inferredBy.Orthology).	4	B4GALT6; PIGA; PIGV; UGCG
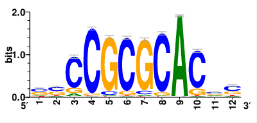	Lipid-glycosylation	jaspar.MA16	3.76	0.2420	ZBTB14 (directAnnotation).	5	B3GALNT1; B3GNT5; B4GALT6; UGCG; UGT8
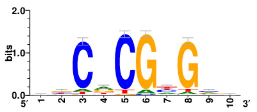	Lipid-glycosylation	transfac.pro.M08005	3.75	0.2410	TCFL5 (inferredBy.Orthology).	5	B3GALNT1; B3GNT5; B4GALT6; UGCG; UGT8
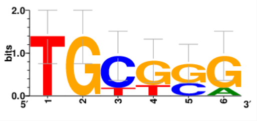	Lipid-glycosylation	hdpi.TGIF1	3.72	0.2390	TGIF1 (directAnnotation).	5	B3GALNT1; B3GNT5; B4GALT6; UGCG; UGT8

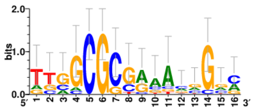	Lipid-glycosylation	transfac.pro.M00938	3.71	0.2390	E2F1 (directAnnotation).	4	B3GNT5; B4GALT6; UGCG; UGT8
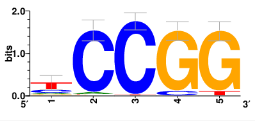	Lipid-glycosylation	metacluster.135.11	3.65	0.2360	NR2C2 (directAnnotation).	4	B3GNT5; B4GALT5; B4GALT6; UGCG
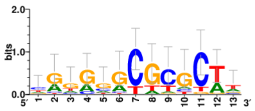	Lipid-glycosylation	transfac.pro.M00333	3.63	0.2350	ZBTB14 (directAnnotation).	3	B4GALT6; UGCG; UGT8
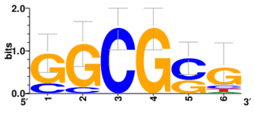	Lipid-glycosylation	transfac.pro.M00803	3.61	0.2340	E2F1; E2F3; E2F4; TFDP1 (directAnnotation).	7	B3GALNT1; B3GNT5; B4GALNT1; B4GALT6; PIGZ; UGCG; UGT8
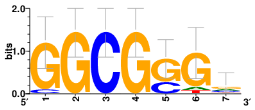	Lipid-glycosylation	transfac.pro.M02089	3.60	0.2340	E2F3 (directAnnotation).	8	B3GALNT1; B3GNT5; B4GALNT1; B4GALT5; B4GALT6; PIGZ; UGCG; UGT8
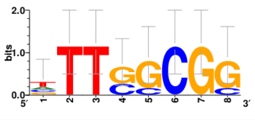	Lipid-glycosylation	transfac.pro.M00430	3.54	0.2310	E2F1 (directAnnotation).	4	B3GNT5; B4GALT6; UGCG; UGT8

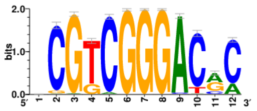	Lipid-glycosylation	transfac.pro.M06126	3.50	0.2290	ZNF43 (directAnnotation).	5	A4GALT; B3GNT5; B4GALT6; UGCG; UGT8
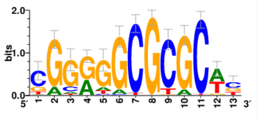	Lipid-glycosylation	swissregulon.	3.45	0.2260	ZBTB14 (directAnnotation).	5	B3GALNT1; B4GALT6; PIGA; UGCG; UGT8
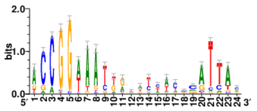	Lipid-glycosylation	taipale. tf. pairs.ELK1. HOXA3. NCCGG-WNNNNNNNNNNNSCAT-TAN. CAP. repr	3.44	0.2260	ELK1; HOXA3 (directAnnotation).	4	A4GALT; B3GALNT1; PIGM; PIGZ
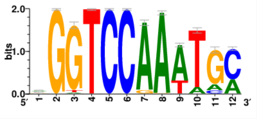	Lipid-glycosylation	transfac.pro.M06059	3.44	0.2250	ZNF727 (directAnnotation).	4	A4GALT; B4GALT5; PIGA; PIGZ
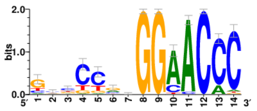	Lipid-glycosylation	dbtfbs.ZBED5 HepG2. ENCSR656SIB. merged. N1	3.43	0.2250	ZBED5 (directAnnotation).	4	B3GNT5; B4GALT5; PIGA; UGCG

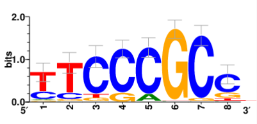	Lipid-glycosylation	metacluster. 15.1	3.41	0.2240	E2F4; E2F4; E2F4; E2F4; E2F7; ZFP69B (directAnnotation). E2F4 (inferredBy.Ort	8	B3GALNT1; B3GNT5; B4GALT5; B4GALT6; PIGM; PIGV; UGCG; UGT8

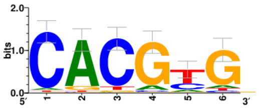	Lipid-glycosylation	metacluster.128.2	3.39	0.2230	BHLHE40;<br>HES1;<br>HES1;<br>HES1;<br>HES1;<br>HES1;<br>HES2;<br>HES5;<br>HES5;<br>HES5;<br>HES7;<br>HES7;<br>HEY1;<br>HEY1;<br>HEY1;<br>HEY1;<br>HEY1;<br>HEY1;<br>HEY2;<br>HEY2;<br>HEY2;<br>HEY2;<br>HEY2;<br>HEY2;<br>HEY2;<br>HIF1A;<br>MGA;<br>MYC;<br>MYCN;<br>SOHLH2 (directAnnotation).<br>BHLHE40;<br>BHLHE40;<br>BHLHE40;<br>BHLHE41;<br>BHLHE41;<br>HELT;<br>HELT;<br>HES1;<br>HES1;<br>HES1;<br>HES1;<br>HES2;<br>HES2;<br>HES3;<br>HES3;<br>HES4;<br>HES4;<br>HES5;<br>HES5;<br>HES5;<br>HES5;<br>HES5;<br>HES5;<br>HES5;<br>HES5;	5	B3GALNT1;<br>B3GNT5;<br>B4GALT6;<br>UGCG;<br>UGT8

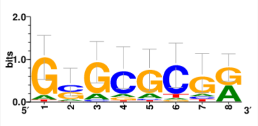	Lipid-glycosylation	transfac.pro.M00716	3.39	0.2230	ZBTB14 (directAnnotation).	8	B3GALNT1; B3GNT5; B4GALNT1; B4GALT5; B4GALT6; PIGZ; UGCG; UGT8
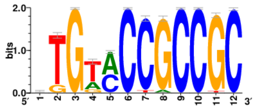	Lipid-glycosylation	transfac.pro.M06163	3.39	0.2230	ZNF121 (directAnnotation).	4	A4GALT; B3GNT5; B4GALT6; PIGZ
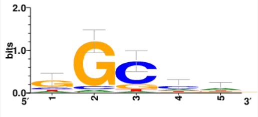	Lipid-glycosylation	metacluster.45.4	3.35	0.2210	E2F2 (directAnnotation).	5	B3GALNT1; B3GNT5; B4GALT6; UGCG; UGT8
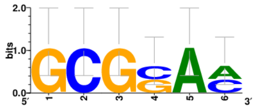	Lipid-glycosylation	hdpi.HTATIP2	3.35	0.2210	HTATIP2 (directAnnotation).	3	B4GALT6; UGCG; UGT8
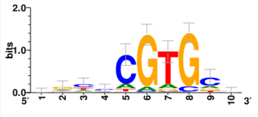	Lipid-glycosylation	cisbp.M0173[illegible]	3.34	0.2200	CCDC169-SOHLH2; SOHLH2 (inferredBy.Ort[illegible]	5	B3GALNT1; B3GNT5; B4GALT6; UGCG; UGT8

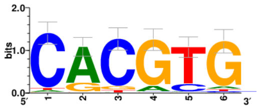	Lipid-glycosylation	metacluster. 198.2	3.29	0.2180	AHR; AHR; ARNT; BHLHE40; MAX; MYC; ZNF780A (directAnnotation). MTF1 (inferredBy.Orthology).	4	B3GALNT1; B4GALT6; UGCG; UGT8
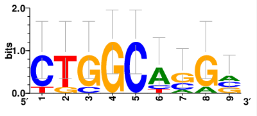	Lipid-glycosylation	transfac. pro.M07051	3.29	0.2180	NFIB (directAnnotation).	4	B3GNT5; B4GALT6; UGCG; UGT8
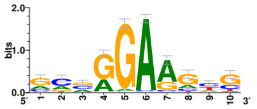	Lipid-glycosylation	hocomoco.FEV HUMAN.H11MO.0.B	3.28	0.2180	FEV (directAnnotation).	5	B3GNT5; B4GALT5; B4GALT6; UGCG; UGT8
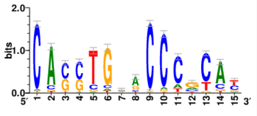	Lipid-glycosylation	taipale. tf. pairs.GCM1. FIGLA. CASSTGNNCCCGCAY. CAP. repr	3.27	0.2170	FIGLA; GCM1 (directAnnotation).	4	B4GALT5; B4GALT6; UGCG; UGT8
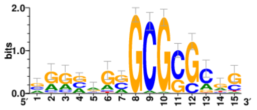	Lipid-glycosylation	cisbp.M07938	3.23	0.2150	E2F1 (directAnnotation).	9	B3GALNT1; B3GNT5; B4GALNT1; B4GALT5; B4GALT6; PIGV; PIGZ; UGCG; UGT8

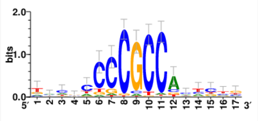	Lipid-glycosylation	transfac. pro.M02898	3.22	0.2140	SMAD3 (directAnnotation).	5	B3GNT5; B4GALT5; B4GALT6; UGCG; UGT8
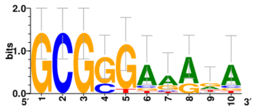	Lipid-glycosylation	transfac. pro.M02090	3.21	0.2140	E2F4 (directAnnotation).	9	B3GALNT1; B3GNT5; B4GALNT1; B4GALT5; B4GALT6; PIGA; PIGM; UGCG; UGT8
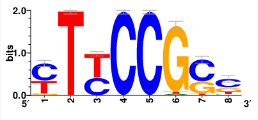	Lipid-glycosylation	dbtfbs.E2F8. representative. N1	3.18	0.2120	E2F8 (directAnnotation).	9	B3GALNT1; B3GNT5; B4GALT5; B4GALT6; PIGB; PIGM; PIGV; PIGZ; UGCG
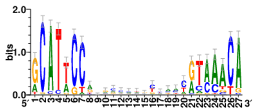	Lipid-glycosylation	taipale. tf. pairs.TEAD4. FOXI1. RCATWCC-NNNNNNNNNNNNNRT-MAACA. CAP. repr	3.15	0.2110	FOXI1; TEAD4 (directAnnotation).	4	B3GNT5; PIGV; UGCG; UGT8

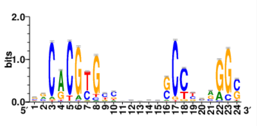	Lipid-glycosylation	taipale. tf. pairs.TFAP2 HES7. NNCRC-GYGNNNNI C-NNNGGS. CAP. repr	3.15	0.2110	HES7; TFAP2C (directAn-notation).	3	B3GNT5; B4GALT6; UGCG
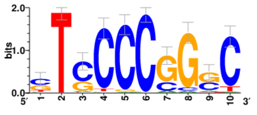	Lipid-glycosylation	cisbp.M00341	3.13	0.2100	PAX6 (directAn-notation).	8	A4GALT; B3GNT5; B4GALNT1; B4GALT5; B4GALT6; PIGZ; UGCG; UGT8
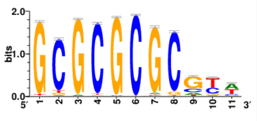	Lipid-glycosylation	taipale. cyt. meth.E2F2. GCGCGCG(GYW. eDBD. repr	3.11	0.2090	E2F2 (directAn-notation).	3	B3GALNT1; B4GALT6; UGCG
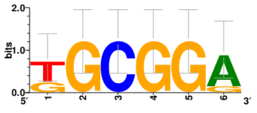	Lipid-glycosylation	hdpi.HIP2	3.10	0.2080	UBE2K (directAn-notation).	4	B3GNT5; B4GALT6; UGCG; UGT8
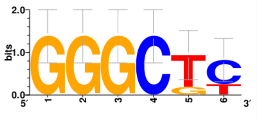	Lipid-glycosylation	hdpi.GTF3C	3.08	0.2070	GTF3C2 (directAn-notation).	4	B3GNT5; B4GALT6; UGCG; UGT8

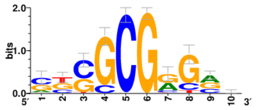	Lipid-glycosylation	transfac. pro.M04817	3.07	0.2070	ZBTB33 (directAn-notation).	8	B3GALNT1; B3GNT5; B4GALNT1; B4GALT5; B4GALT6; PIGZ; UGCG; UGT8
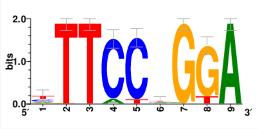	N-Glycosylatio	transfac. pro.M04721	5.28	0.1610	STAT1 (directAn-notation).	7	ALG10; ALG10B; DPAGT1; FUT8; MGAT1; MGAT3; MGAT4B
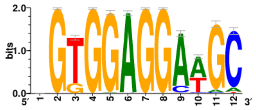	N-Glycosylation	transfac. pro.M06483	5.18	0.1590	ZNF595 (directAn-notation).	5	ALG14; FUT8; MGAT4A; MGAT4D; STT3B
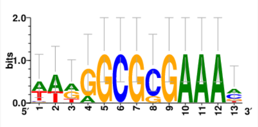	N-Glycosylatio	swissregulon.	5.10	0.1570	E2F7 (in-ferredBy.Ort	4	ALG10; ALG10B; FUT8; STT3B
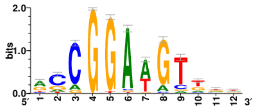	N-Glycosylation	taipale. tf. pairs.FOXO1. ELK3. RCCG-GAWGTKKW. CAP	4.89	0.1510	ELK3; FOXO1 (directAn-notation).	5	ALG10; ALG10B; MGAT1; UGGT1; UGGT2

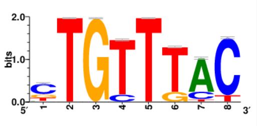	N-Glycosylatio	swissregulon.	4.87	0.1510	FOXO1 (directAn-notation).	6	ALG10; ALG10B; ALG12; DPAGT1; MGAT1; MGAT4C
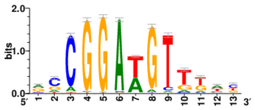	N-Glycosylation	taipale. tf. pairs.ERF. FOXI1. RSCG-GATGT-TKWN. CAP. repr	4.76	0.1480	ERF; FOXI1 (directAn-notation).	12	ALG10; ALG10B; ALG13; ALG8; DPAGT1; FUT8; MGAT1; MGAT4C; MGAT5; STT3B; UGGT1; UGGT2
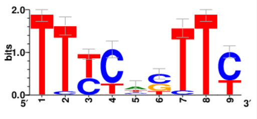	N-Glycosylatio	transfac. pro.M04860	4.71	0.1470	RXRA (directAn-notation).	10	ALG10; ALG10B; ALG5; MGAT1; MGAT3; MGAT4A; MGAT4C; MGAT5; UGGT1; UGGT2
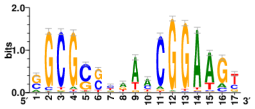	N-Glycosylation	taipale. tf. pairs.E2F1. ELK1. SGCGC-NNNNNCG-GAAGN. CAP. repr	4.54	0.1420	E2F1; ELK1 (directAn-notation).	12	ALG10; ALG10B; ALG6; ALG8; DPM1; FUT8; MGAT4B; MGAT4D; MGAT5; STT3A; STT3B; UGGT2

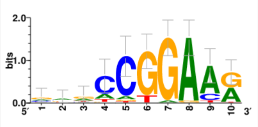	N-Glycosylatio	swissregulon.	4.32	0.1370	ELK1 (directAn-notation).	4	ALG10; ALG10B; FUT8; UGGT2
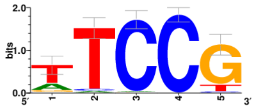	N-Glycosylation	metacluster. 191.5	4.32	0.1370	SPDEF (in-ferredBy.Orthology).	5	ALG10; ALG10B; FUT8; STT3B; UGGT2
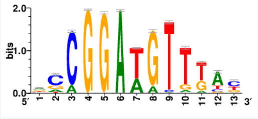	N-Glycosylatio	taipale. tf. pairs.FLI1. FOXI1. RCCG-GATGT-TKWY. CAP	4.31	0.1370	FLI1; FOXI1 (directAn-notation).	11	ALG10; ALG10B; ALG13; ALG8; FUT8; MGAT1; MGAT3; MGAT4C; MGAT5; UGGT1; UGGT2
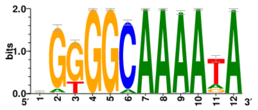	N-Glycosylation	transfac. pro.M06671	4.18	0.1330	ZNF285 (directAn-notation).	5	ALG10; ALG10B; ALG13; ALG14; MGAT4C
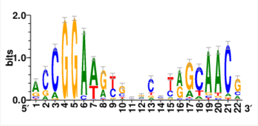	N-Glycosylatio	taipale. tf. pairs.ETV2. RFX5. RNCG-GAANNNN CAACN. CAP. repr	4.12	0.1320	ETV2; RFX5 (directAn-notation).	7	ALG1; FUT8; MGAT3; MGAT4A; MGAT4B; STT3B; UGGT1

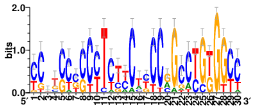	N-Glycosylation	kznf.ZIM2. Im-beault2017. RP. ChIP-seq	4.07	0.1300	ZIM2 (directAn-notation).	4	ALG10; ALG10B; ALG8; STT3B
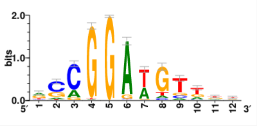	N-Glycosylatioı	taipale. tf. pairs.FOXO1 ETV1. RCCG-GAWGTKK: CAP	4.06	0.1300	ETV1; FOXO1 (directAn-notation).	9	ALG10; ALG10B; DPM1; FUT8; MGAT1; MGAT5; STT3B; UGGT1; UGGT2
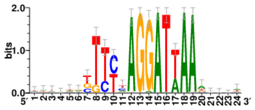	N-Glycosylation	tfdimers.MD00[illegible]	4.06	0.1300	ARID3A; BCL6 (directAn-notation).	5	ALG1; ALG10; ALG10B; MGAT4A; MGAT5
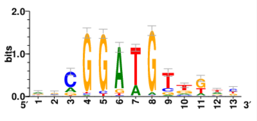	N-Glycosylatioı	taipale. tf. pairs.ETV5. FOXO1. RNCG-GATGT-TKWN. CAP	4.03	0.1290	ETV5; FOXO1 (directAn-notation).	12	ALG10; ALG10B; ALG8; DPM1; FUT8; MGAT1; MGAT4C; MGAT4D; MGAT5; STT3B; UGGT1; UGGT2

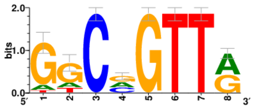	N-Glycosylation	homer.GGCVGTTR. MYB	3.89	0.1260	MYB (inferredBy.Orthology).	14	ALG1; ALG10; ALG10B; ALG11; ALG13; ALG2; ALG3; ALG5; DPM1; FUT8; MGAT2; MGAT4C; STT3B; UGGT2
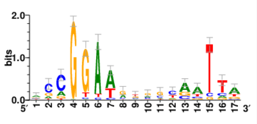	N-Glycosylatio	taipale. tf. pairs.ERF. DLX3. RSCG-GAANNNNY CAP. repr	3.84	0.1240	DLX3; ERF (directAnnotation).	4	ALG10; ALG10B; DPAGT1; UGGT2

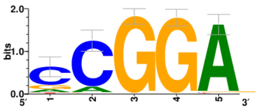	N-Glycosylation	metacluster.138.2	3.82	0.1240	ELF1; ELF2; ELF4; ELK1; ELK1; ELK1; ELK1; ELK3; ELK4; ELK4; ELK4; ELK4; ELK4; ELK4; ELK4; ERF; ERG; ERG; ERG; ERG; ETS1; ETS1; ETS2; ETS2; ETV1; ETV1; ETV1; ETV2; ETV3; ETV4; ETV5; ETV5; FEV; FLI1; FLI1; GABPA; GABPA; KMT2B; SPDEF; SPDEF; SPDEF; ZBTB11; ZNF518A; ZNF580 (directAnnotation). ELF1; ELF2; ELF4; ELK1; ELK1; ELK3; ELK4; ERG; ETS1; ETS2; ETV1;	10	ALG10; ALG10B; ALG6; ALG8; DPM1; FUT8; MGAT1; STT3B; UGGT1; UGGT2

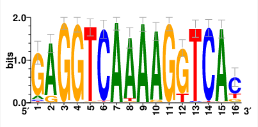	N-Glycosylatio	metacluster. 84.14	3.79	0.1230	RARG (directAnnotation).	5	ALG10; ALG10B; ALG6; FUT8; MGAT4C
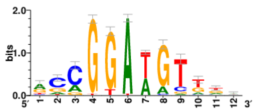	N-Glycosylation	taipale. tf. pairs.FOXO1. ETV4. RCCG-GAWGTKKN. CAP	3.78	0.1230	ETV4; FOXO1 (directAnnotation).	6	ALG10; ALG10B; MGAT1; MGAT5; UGGT1; UGGT2
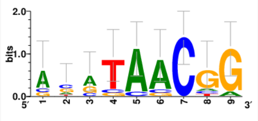	N-Glycosylatio	transfac. pro.M08894	3.77	0.1230	OVOL1; OVOL2 (directAnnotation).	4	ALG10; ALG10B; FUT8; UGGT2
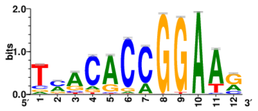	N-Glycosylation	taipale. tf. pairs.ERF. TBX21. TMACAC-CGGAAG. CAP	3.76	0.1220	ERF; TBX21 (directAnnotation).	4	ALG10; ALG10B; DPM1; UGGT2
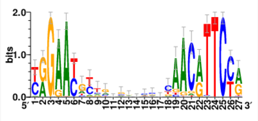	N-Glycosylatio	metacluster. 62.3	3.73	0.1220	HSF5 (directAnnotation).	5	ALG10B; DPAGT1; MGAT1; MGAT4A; MGAT4C
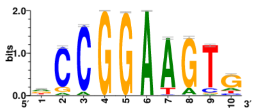	N-Glycosylation	taipale. tf. pairs.ETS2. RCCG-GAAGTG. HT	3.72	0.1220	ETS2 (directAnnotation).	5	ALG10; ALG10B; MGAT1; UGGT1; UGGT2

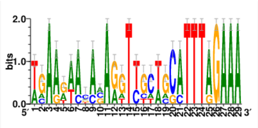	N-Glycosylatio	kznf.ZNF25. Imbeault2017. RP. ChIP-seq	3.72	0.1210	ZNF25 (directAnnotation).	9	ALG10; ALG10B; ALG12; ALG14; ALG2; DPM1; FUT8; MGAT4C; MGAT5
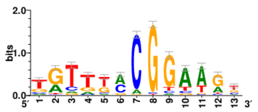	N-Glycosylation	taipale. tf. pairs.ERF. FOXI1. TGTTKM-CG-GAWRN. CAP. repr	3.71	0.1210	ERF; FOXI1 (directAnnotation).	5	ALG10; ALG10B; STT3B; UGGT1; UGGT2
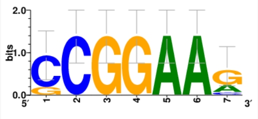	N-Glycosylatio	hdpi.ELF2	3.68	0.1200	ELF2 (directAnnotation).	6	ALG10; ALG10B; FUT8; STT3B; UGGT1; UGGT2
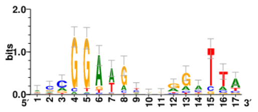	N-Glycosylation	taipale. tf. pairs.ERF. PITX1. NSCG-GANNNNNG-GMTTA. CAP	3.66	0.1200	ERF; PITX1 (directAnnotation).	5	ALG10; ALG10B; FUT8; STT3B; UGGT2
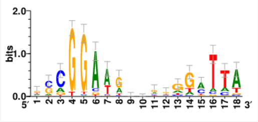	N-Glycosylatio	taipale. tf. pairs.ERF. PITX1. NSCG-GANNNNN GMTTA. CAP	3.60	0.1190	ERF; PITX1 (directAnnotation).	5	ALG10; ALG10B; FUT8; STT3B; UGGT2

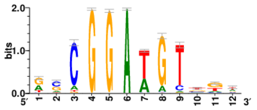	N-Glycosylation	taipale. tf. pairs.ETV5. FOXI1. RSCG-GAT-GTTGN. CAP	3.60	0.1190	ETV5; FOXI1 (directAn-notation).	4	ALG10; ALG10B; MGAT1; UGGT1
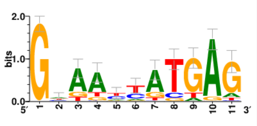	N-Glycosylation	metacluster. 172.8	3.58	0.1180	SIX1 (directAn-notation).	5	ALG10; ALG10B; ALG3; MGAT4B; STT3B
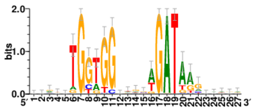	N-Glycosylation	tfdimers.MD00[illegible]	3.5[illegible]	0.1170	GATA1; GATA2; GATA3; GATA4; GATA5; GATA6; ING4 (directAn-notation).	6	ALG10; ALG10B; ALG2; MGAT3; MGAT4D; STT3B
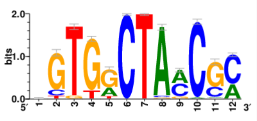	N-Glycosylation	transfac. pro.M06188	3.48	0.1150	ZNF135 (directAn-notation).	6	ALG12; FUT8; MGAT3; MGAT4A; STT3B; UGGT1
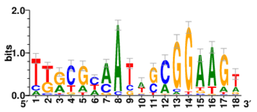	N-Glycosylation	taipale. tf. pairs.CEBPG. ELF1. TTGC-GYAANNSCG-GAAGN. CAP. repr	3.39	0.1130	CEBPG; ELF1 (directAn-notation).	4	ALG10; ALG10B; MGAT3; MGAT4C

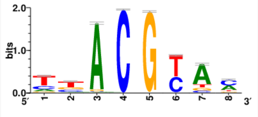	N-Glycosylatio	swissregulon.	3.38	0.1130	GMEB2 (directAn-notation).	9	ALG1; ALG10; ALG13; ALG14; DPM1; MGAT1; MGAT4A; MGAT4B; MGAT5
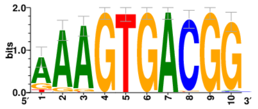	N-Glycosylation	metacluster. 141.13	3.37	0.1130	METTL14 (in-ferredBy.Orthology).	4	ALG10; ALG10B; ALG12; ALG9
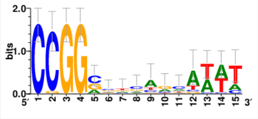	N-Glycosylatio	transfac. pro.M01298	3.36	0.1120	MECP2 (directAn-notation).	4	ALG10; ALG10B; STT3B; UGGT2
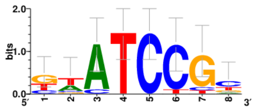	N-Glycosylation	transfac. pro.M07612	3.32	0.1110	SFPQ (directAn-notation).	10	ALG10; ALG10B; ALG6; FUT8; MGAT1; MGAT4B; MGAT4D; STT3A; STT3B; UGGT2
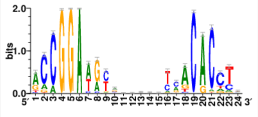	N-Glycosylatio	taipale. tf. pairs.ETV2. EOMES. RCCG-GANNNNN CTN. CAP. repr	3.28	0.1100	EOMES; ETV2 (directAn-notation).	3	ALG10; STT3B; UGGT2

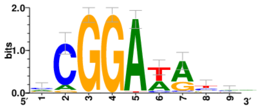	N-Glycosylation	cisbp.M02645	3.28	0.1100	ETS1 (directAnnotation).	6	ALG10; ALG10B; DPAGT1; FUT8; UGGT1; UGGT2
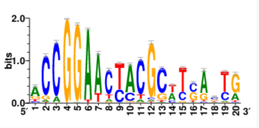	N-Glycosylatio	taipale. tf. pairs.ETV2. PAX5. ACCG-GAN-NTACGC-NNNNNYR. CAP	3.25	0.1100	ETV2; PAX5 (directAnnotation).	4	ALG10; ALG10B; ALG12; STT3B
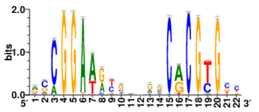	N-Glycosylation	taipale. tf. pairs.ETV2. HES7. RSCG-GAANNNNNNNCACGT-GNN. CAP. repr	3.23	0.1090	ETV2; HES7 (directAnnotation).	6	ALG1; ALG5; FUT8; MGAT3; MGAT4A; UGGT2
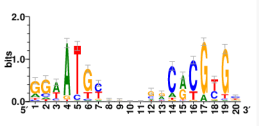	N-Glycosylatio	taipale. tf. pairs.TEAD4 HES7. GGWATG-YNNNNNNC CGYGY. CAP	3.22	0.1090	HES7; TEAD4 (directAnnotation).	6	ALG10; ALG10B; ALG6; FUT8; MGAT4D; UGGT2

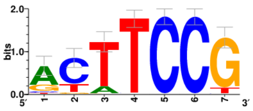	N-Glycosylation	metacluster.191.3	3.22	0.1090	EHF; ELF1; ELF1; ELF1; ELF4; ELK1; ELK3; ELK3; ELK4; ELK4; ERF; ETS1; ETS2; ETV3; ETV4; ETV5; FEV; FEV; FLI1; FLI1; GABPA; GABPA; GABPA; GABPA; GABPA; GABPA; SPDEF; ZNF200 (directAnnotation). ELK1; ELK1; ELK3; ELK3; ELK4; ELK4; ETV1; ETV1; ETV4; ETV4; ETV4; ETV5; GABPA; GABPA (inferredBy.Orthology).	4	ALG10; ALG10B; UGGT1; UGGT2

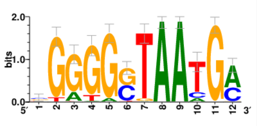	N-Glycosylatio	metacluster. 152.8	3.19	0.1080	ZNF208 (directAnnotation).	5	ALG8; DPAGT1; MGAT4A; MGAT4C; STT3B
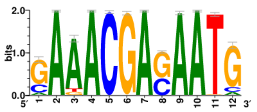	N-Glycosylation	transfac. pro.M06662	3.17	0.1070	ZNF426 (directAnnotation).	2	ALG10; ALG10B
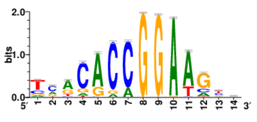	N-Glycosylatio	taipale. tf. pairs.ELK1. TBX21. TNRCACCG-GAAGNN. CAP. repr	3.15	0.1070	ELK1; TBX21 (directAnnotation).	4	ALG10; ALG10B; DPM1; UGGT2
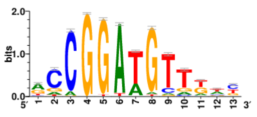	N-Glycosylation	taipale. tf. pairs.ETV2. FOXO6. RCCG-GATGT-TKWN. CAP	3.14	0.1070	ETV2; FOXO6 (directAnnotation).	11	ALG10; ALG10B; ALG13; ALG3; ALG8; DPAGT1; FUT8; MGAT1; MGAT4C; UGGT1; UGGT2
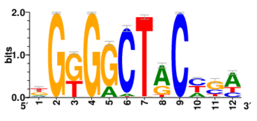	N-Glycosylatio	transfac. pro.M06222	3.14	0.1070	ZNF273 (directAnnotation).	9	ALG11; ALG6; ALG8; FUT8; MGAT1; MGAT4A; MGAT4B; MGAT4D; STT3B

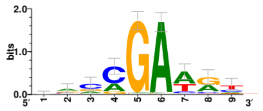	N-Glycosylation	metacluster. 183.4	3.12	0.1060	ETS1 (inferredBy.Orthology).	4	ALG10; ALG10B; FUT8; UGGT2
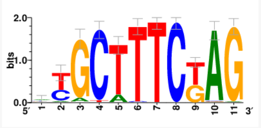	N-Glycosylation	metacluster. 93.4	3.11	0.1060	BCL6B (directAnnotation).	4	FUT8; MGAT3; MGAT4C; UGGT1
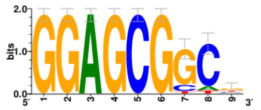	N-Glycosylation	kznf.ZNF610. Imbeault2017. OM. RCADE	3.09	0.1050	ZNF610 (directAnnotation).	4	FUT8; MGAT4A; STT3B; UGGT2
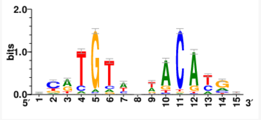	N-Glycosylation	taipale. cyt. meth.IRX5. NCRTGTNNNACA eDBD. repr	3.07	0.1050	IRX5 (directAnnotation).	5	ALG10; ALG10B; DPM1; MGAT1; MGAT5
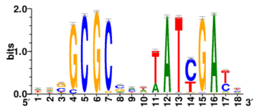	N-Glycosylation	taipale. tf. pairs.E2F3. ONECUT2. NNSGCGCSNNNATCGAYN. CAP. repr	3.07	0.1050	E2F3; ONECUT2 (directAnnotation).	6	ALG10; ALG10B; ALG5; ALG9; FUT8; STT3B

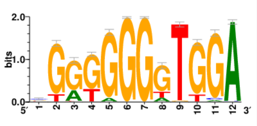	N-Glycosylatio	transfac. pro.M06051	3.06	0.1050	ZNF208 (directAnnotation).	7	ALG10; ALG10B; ALG14; DPM1; FUT8; MGAT1; UGGT1
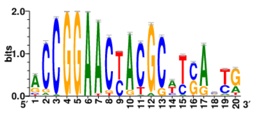	N-Glycosylation	taipale. tf. pairs.ELK1. PAX5. RSCG-GAACY-ACGCWYSANTG. CAP. repr	3.05	0.1050	ELK1; PAX5 (directAnnotation).	5	ALG10; ALG10B; ALG12; ALG5; STT3B
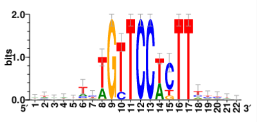	N-Glycosylatio	tfdimers.MD	3.04	0.1040	IRF1; NR3C1 (directAnnotation).	6	ALG10; ALG10B; ALG13; MGAT3; MGAT5; STT3B
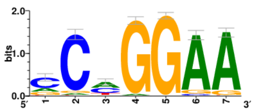	N-Glycosylation	transfac. pro.M01776	3.03	0.1040	NR2C2 (directAnnotation).	3	ALG10; ALG10B; FUT8
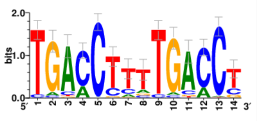	N-Glycosylatio	metacluster. 84.12	3.02	0.1040	RARB; RARG (directAnnotation).	3	ALG10; ALG10B; MGAT4C
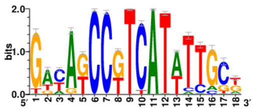	N-Glycosylation	transfac. pro.M06880	3.02	0.1040	ZFP82 (inferredBy.Orthology).	5	ALG10; ALG10B; MGAT4A; STT3B; UGGT2

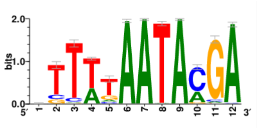	N-Glycosylatio	transfac.pro.M05799	3.02	0.1040	ZNF605 (directAnnotation).	15	ALG10; ALG10B; ALG12; ALG6; DPAGT1; DPM1; FUT8; MGAT1; MGAT3; MGAT4A; MGAT4D; MGAT5; STT3A; STT3B; UGGT2
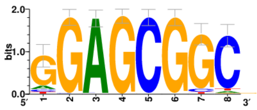	N-Glycosylation	metacluster.144.5	3.01	0.1030	ZNF610; ZNF610; ZNF610 (directAnnotation).	3	FUT8; MGAT4A; STT3B
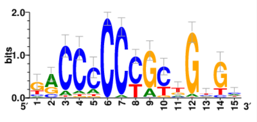	O-glycosylation (mucin)	metacluster.80.6	3.87	0.1900	ZIC4 (directAnnotation).	6	C1GALT1; C1GALT1C1; GALNT13; GALNT3; GALNT7; GALNT9
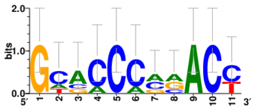	O-glycosylation (mucin)	transfac.pro.M04631	3.45	0.1750	RREB1 (directAnnotation).	4	C1GALT1; GALNT13; GALNT3; GALNT9

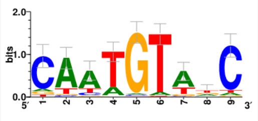	O-glycosylation (mucin)	metacluster.69.2	3.42	0.1730	DMRT1; DMRT1 (directAnnotation). DMRTB1; DMRTB1 (inferredBy.Ort	6	GALNT10; GALNT11; GALNT2; GALNT3; GALNT6; GALNT9
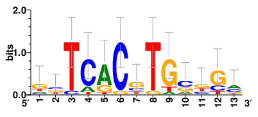	O-glycosylation (mucin)	transfac.pro.M08871	3.39	0.1720	BHLHE41 (directAnnotation).	4	C1GALT1; GALNT13; GALNT3; GALNTL6
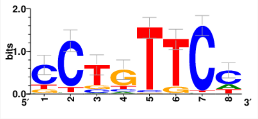	O-glycosylation (mucin)	metacluster.167.3	3.33	0.1700	ZNF189; ZNF84; ZNF84 (directAnnotation).	10	B3GNT6; C1GALT1; C1GALT1C1; GALNT13; GALNT16; GALNT3; GALNT7; GALNT8; GALNTL6; GCNT1
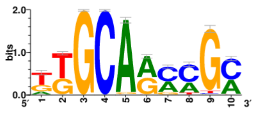	O-glycosylation (mucin)	transfac.pro.M06069	3.32	0.1690	ZNF253 (directAnnotation).	4	C1GALT1; GALNT14; GALNT15; GALNTL6
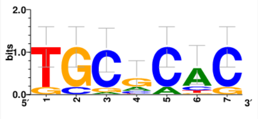	O-glycosylation (mucin)	transfac.pro.M01243	3.28	0.1680	MTF1 (directAnnotation).	5	C1GALT1; GALNT13; GALNT3; GALNT9; GALNTL6

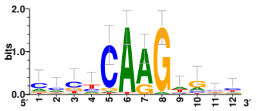	O-glycosylation (mucin)	transfac. pro.M00794	3.28	0.1680	NKX2-1 (directAn-notation).	4	C1GALT1; GALNT13; GALNT3; GALNTL6
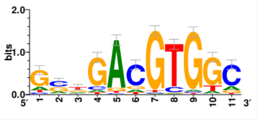	O-glycosylation (mucin)	hocomoco.XI HU-MAN.H11M(	3.26	0.1670	XBP1 (directAn-notation).	4	C1GALT1; GALNT13; GALNT3; GALNT9
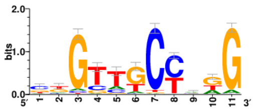	O-glycosylation (mucin)	hocomoco.RFX6. MOUSE.H11MO.0.C	3.17	0.1640	RFX6 (in-ferredBy.Orthology).	4	C1GALT1; GALNT3; GALNT9; GALNTL6
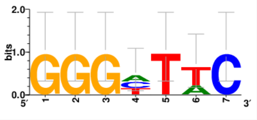	O-glycosylation (mucin)	hdpi.HCFC2	3.16	0.1630	HCFC2 (directAn-notation).	3	GALNT3; GALNT9; GALNTL6
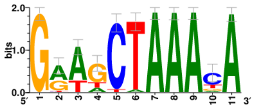	O-glycosylation (mucin)	metacluster. 134.5	3.13	0.1620	ZNF184 (directAn-notation).	4	GALNT10; GALNT12; GALNT5; GALNTL6
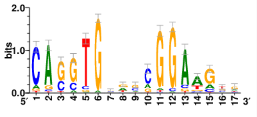	O-glycosylation (mucin)	taipale. tf. pairs.ETV5. TCF3. CASST-GNRNNG-GAAGNN. CAP	3.10	0.1610	ETV5; TCF3 (directAn-notation).	4	C1GALT1; GALNT13; GALNT3; GALNTL6
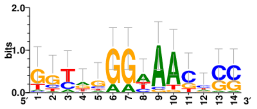	O-glycosylation (mucin)	hocomoco.ZEP2. HU-MAN.H11MO.0.D	3.09	0.1610	HIVEP2 (directAn-notation).	3	GALNT3; GALNT9; GALNTL6

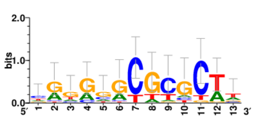	O-glycosylation (mucin)	transfac.pro.M00333	3.04	0.1590	ZBTB14 (directAnnotation).	4	C1GALT1; GALNT13; GALNT3; GALNTL6
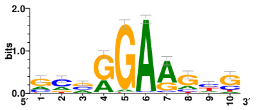	O-glycosylation (mucin)	hocomoco.FEV HUMAN.H11MO.0.B	3.01	0.1580	FEV (directAnnotation).	4	C1GALT1; GALNT13; GALNT3; GALNTL6
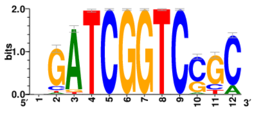	O-glycosylation (non-mucin)	transfac.pro.M05931	5.28	0.1260	ZNF556 (directAnnotation).	11	CHPF2; CHSY1; CSGALNACT2; EOGT; EXT1; EXT2; GXYLT2; POFUT2; TMTC2; TMTC3; XXYLT1
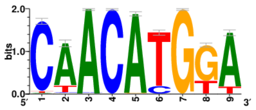	O-glycosylation (non-mucin)	transfac.pro.M05961	5.28	0.1260	ZNF407 (directAnnotation).	8	CHSY3; CSGALNACT1; CSGALNACT2; GXYLT1; GXYLT2; POMGNT1; POMT1; TMTC1

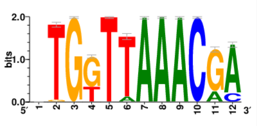	O-glycosylation (non-mucin)	transfac. pro.M06150	4.80	0.1170	ZNF81 (directAnnotation).	7	B3GALT6; CHSY3; COLGALT2; CSGALNACT1; EXTL3; POFUT2; TMTC2
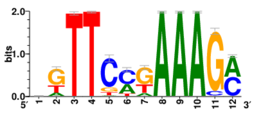	O-glycosylation (non-mucin)	transfac. pro.M06677	4.66	0.1150	ZNF285 (directAnnotation).	9	CHPF2; MGAT5B; POFUT2; POGLUT1; POMGNT1; TMTC1; TMTC3; XXYLT1; XYLT1
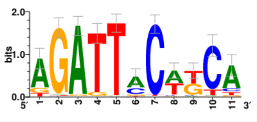	O-glycosylation (non-mucin)	metacluster. 48.1	4.32	0.1080	ZNF85; ZNF85 (directAnnotation).	4	B3GALNT2; B4GALT7; EXTL3; TMTC4
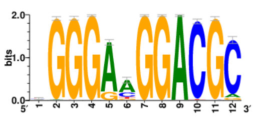	O-glycosylation (non-mucin)	transfac. pro.M06074	4.20	0.1060	ZNF732 (directAnnotation).	5	CHSY1; EOGT; GXYLT1; TMTC2; XYLT2
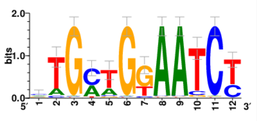	O-glycosylation (non-mucin)	metacluster. 46.3	4.14	0.1050	ZNF85 (directAnnotation).	8	B3GALNT2; B4GALT7; CHSY1; EXTL3; OGT; POFUT1; TMTC2; XYLT1

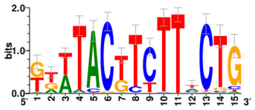	O-glycosylation (non-mucin)	metacluster. 187.3	3.99	0.1020	SPDEF; SPDEF (directAnnotation).	12	B3GALNT2; CHSY1; CSGALNACT2; EXT1; EXTL3; GXYLT2; OGT; POGLUT1; POMGNT2; POMT1; TMTC4; XXYLT1
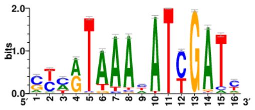	O-glycosylation (non-mucin)	taipale. tf. pairs.CUX1. HOXB13. SYMR-TAAANATY GATN. CAP	3.73	0.0971	CUX1; HOXB13 (directAnnotation).	5	EXT1; GXYLT2; OGT; POFUT2; TMTC2
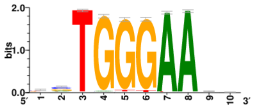	O-glycosylation (non-mucin)	jaspar.MA1116.1	3.71	0.0965	RBPJ (directAnnotation).	5	CHSY1; EOGT; LFNG; MFNG; XXYLT1
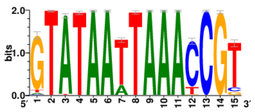	O-glycosylation (non-mucin)	transfac. pro.M06943	3.67	0.0959	PBX3 (directAnnotation).	10	B3GALNT2; B3GALT6; B3GLCT; CHSY3; COLGALT2; CSGALNACT1; MGAT5B; OGT; TMTC1; TMTC2

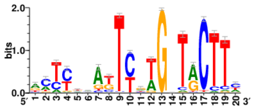	O-glycosylation (non-mucin)	swissregulon.hs.DMC1	3.61	0.0946	DMC1 (directAnnotation).	5	B3GLCT; CHSY1; LFNG; POFUT2; TMTC1
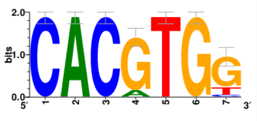	O-glycosylation (non-mucin)	metacluster.10.30	3.54	0.0934	MXI1 (inferredBy.Ort	5	CSGALNACT1; LFNG; POFUT2; TMTC1; TMTC2
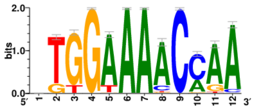	O-glycosylation (non-mucin)	transfac.pro.M05671	3.54	0.0933	ZNF25 (directAnnotation).	5	MGAT5B; POFUT2; TMTC1; TMTC2; XXYLT1
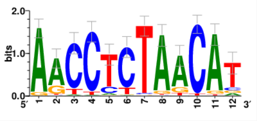	O-glycosylation (non-mucin)	kznf.ZN595.HUMAN.Schmitges2016.RCADE	3.52	0.0930	ZNF595 (directAnnotation).	15	CHSY3; COLGALT2; CSGALNACT1; CSGALNACT2; EOGT; EXT1; EXT2; LFNG; MGAT5B; POFUT2; POMGNT1; TMTC1; TMTC2; TMTC4; XYLT1

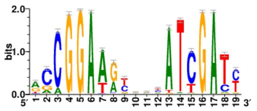	O-glycosylation (non-mucin)	taipale.tf.pairs.ETV2.ONECUT2.RCCGGAANNNNNNATCGATN.CAP.repr	3.46	0.0918	ETV2; ONECUT2 (directAnnotation).	4	CSGALNACT1; LFNG; POMGNT1; RFNG
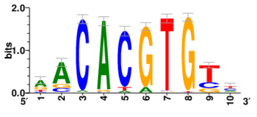	O-glycosylation (non-mucin)	swissregulon.	3.38	0.0903	CLOCK (directAnnotation).	5	CSGALNACT1; LFNG; POFUT2; TMTC1; TMTC2
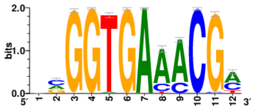	O-glycosylation (non-mucin)	transfac.pro.M05981	3.37	0.0902	ZNF233 (directAnnotation).	5	CSGALNACT1; CSGALNACT2; EXTL3; LFNG; TMTC1
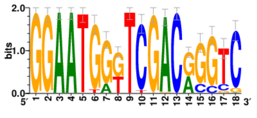	O-glycosylation (non-mucin)	metacluster.27.3	3.34	0.0896	ZNF658 (directAnnotation).	5	CHSY3; EXT1; EXTL1; TMTC3; XYLT2
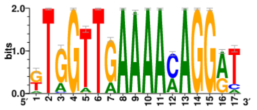	O-glycosylation (non-mucin)	transfac.pro.M05453	3.34	0.0896	ZBTB26 (directAnnotation).	4	CHSY1; EXTL3; MGAT5B; XYLT1
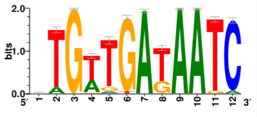	O-glycosylation (non-mucin)	transfac.pro.M06542	3.33	0.0894	ZNF502 (directAnnotation).	6	CSGALNACT2; EXT2; GXYLT2; POMGNT2; TMTC2; XXYLT1

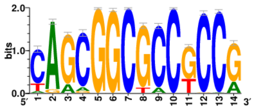	O-glycosylation (non-mucin)	transfac. pro.M05830	3.31	0.0890	ZNF66 (directAnnotation).	8	CHSY1; COLGALT2; CSGALNACT2; EXT1; GXYLT2; LFNG; POMGNT2; TMTC1
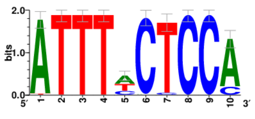	O-glycosylation (non-mucin)	metacluster. 168.17	3.30	0.0889	ZNF417; ZNF587 (directAnnotation).	4	EXTL3; GXYLT2; MGAT5B; XYLT1
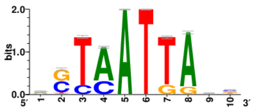	O-glycosylation (non-mucin)	taipale. tf. pairs.HOXA3. NSTAAT-TANN. HT	3.26	0.0881	HOXA3 (directAnnotation).	6	B3GLCT; EOGT; EXT1; EXTL1; MFNG; MGAT5B
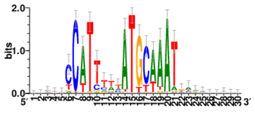	O-glycosylation (non-mucin)	tfdimers.MD	3.24	0.0877	POU2F2; YY1 (directAnnotation).	5	B3GALT6; LFNG; MGAT5B; POMGNT1; XXYLT1
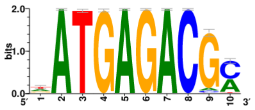	O-glycosylation (non-mucin)	transfac. pro.M06041	3.19	0.0867	ZNF726 (directAnnotation).	9	B4GALT7; CHPF; CHSY3; COLGALT2; EXTL1; OGT; POFUT2; TMTC1; TMTC2

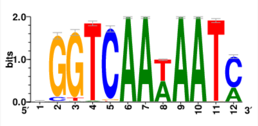	O-glycosylation (non-mucin)	transfac. pro.M06199	3.18	0.0866	ZNF135 (directAnnotation).	12	CHSY1; CHSY3; COLGALT2; EOGT; EXTL1; EXTL2; EXTL3; GXYLT2; POMGNT2; POMT2; TMTC3; XYLT1
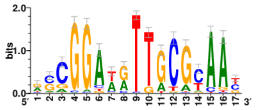	O-glycosylation (non-mucin)	taipale. tf. pairs.ETV5. CEBPD. NSCGGANNTTRCGYAAN. CAP	3.18	0.0866	CEBPD; ETV5 (directAnnotation).	5	B3GALT6; B3GAT3; EXTL2; EXTL3; OGT
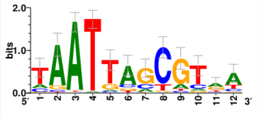	O-glycosylation (non-mucin)	metacluster. 150.8	3.17	0.0865	PAX4; PAX4 (directAnnotation).	12	B4GAT1; CSGALNACT1; EXT1; EXTL1; GXYLT1; MFNG; MGAT5B; OGT; POMT1; TMTC1; TMTC2; XXYLT1

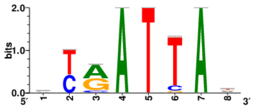	O-glycosylation (non-mucin)	jaspar.MA0707.1	3.15	0.0860	MNX1 (directAnnotation).	13	B3GLCT; CHSY3; EXT1; EXTL1; GXYLT2; LFNG; MFNG; MGAT5B; POFUT1; POMGNT1; POMGNT2; TMTC3; TMTC4
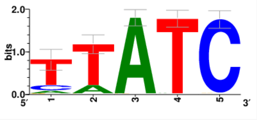	O-glycosylation (non-mucin)	metacluster.117.2	3.15	0.0859	GATA3; GATA3; GATA3; GATA5; GATA5; GATA5; GATA5; GATA6; GATA6 (directAnnotation).	7	B4GALT7; CSGALNACT1; GXYLT1; LFNG; POGLUT1; RFNG; TMTC2
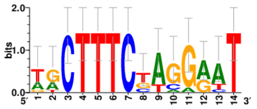	O-glycosylation (non-mucin)	transfac.pro.M01185	3.14	0.0858	BCL6 (directAnnotation).	4	LFNG; MFNG; TMTC2; XYLT1
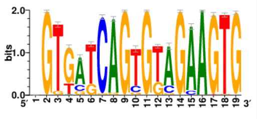	O-glycosylation (non-mucin)	transfac.pro.M05933	3.11	0.0853	ZNF552 (directAnnotation).	6	B3GALT6; CSGALNACT1; EXTL1; EXTL2; MFNG; MGAT5B

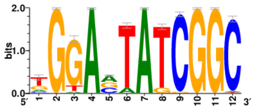	O-glycosylation (non-mucin)	transfac.pro.M06493	3.11	0.0852	ZNF668 (inferredBy.Orthology).	4	COLGALT2; POFUT2; POMGNT2; TMTC4
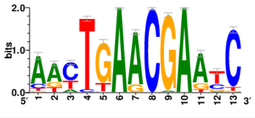	O-glycosylation (non-mucin)	transfac.pro.M05517	3.11	0.0852	IKZF2 (directAnnotation).	6	B3GLCT; B4GALT7; EXT1; EXTL1; POGLUT1; TMTC1
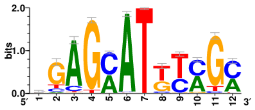	O-glycosylation (non-mucin)	transfac.pro.M06813	3.06	0.0844	SALL4 (inferredBy.Orthology).	4	B3GLCT; CSGALNACT2; POFUT1; TMTC1
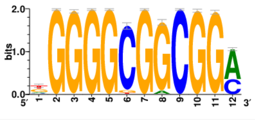	O-glycosylation (non-mucin)	transfac.pro.M05712	3.06	0.0844	ZNF134 (directAnnotation).	18	B3GALNT2; B3GLCT; B4GALT7; CHSY3; COLGALT2; CSGALNACT2; EXTL3; GXYLT1; GXYLT2; LFNG; OGT; POMGNT2; TMTC1; TMTC2; TMTC3; TMTC4; XYLT1; XYLT2

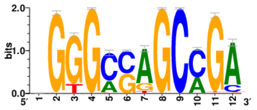	O-glycosylation (non-mucin)	transfac. pro.M06769	3.05	0.0842	ZNF391 (directAnnotation).	8	CHSY3; CSGALNACT1; EOGT; EXT2; EXTL2; EXTL3; TMTC2; XYLT2
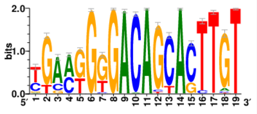	O-glycosylation (non-mucin)	transfac. pro.M06409	3.05	0.0841	ZNF329 (directAnnotation).	3	EXTL1; EXTL3; MGAT5B
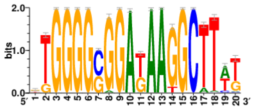	O-glycosylation (non-mucin)	transfac. pro.M05379	3.02	0.0836	ZNF740 (inferredBy.Orthology).	5	CHSY1; EXTL3; GXYLT2; MGAT5B; XYLT1
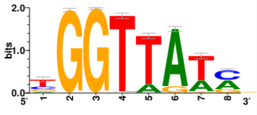	O-glycosylation (non-mucin)	transfac. pro.M05545	3.01	0.0834	IKZF4 (inferredBy.Ort	5	CSGALNACT2; LFNG; MFNG; OGT; POFUT2
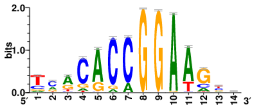	Secretory C-mannosylation	taipale. tf. pairs.ELK1. TBX21. TNRCACCG-GAAGNN. CAP. repr	5.71	0.5830	ELK1; TBX21 (directAnnotation).	3	DPY19L1; DPY19L2; DPY19L4

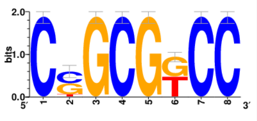	Secretory C-mannosylatic	kznf.ZNF783 Imbeault2017. OM. MEME	5.63	0.5760	ZNF783 (directAnnotation).	3	DPY19L1; DPY19L3; DPY19L4
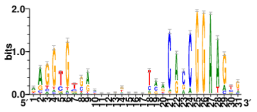	Secretory C-mannosylation	taipale. tf. pairs.ELK1. TBX21. RAGGT-SRNNNNNNNNNNNNNNNNCG-GAAGYN. CAP	5.49	0.5630	ELK1; TBX21 (directAnnotation).	3	DPY19L1; DPY19L2; DPY19L4
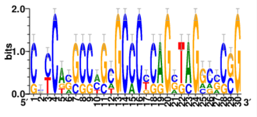	Secretory C-mannosylatic	kznf.ZNF792 Imbeault2017. OM. MEME	4.79	0.4980	ZNF792 (directAnnotation).	3	DPY19L1; DPY19L3; DPY19L4
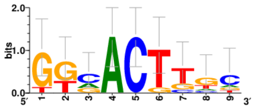	Secretory C-mannosylation	transfac. pro.M01692	4.49	0.4700	LTF (directAnnotation).	2	DPY19L1; DPY19L2
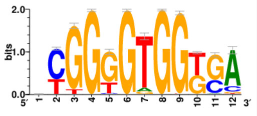	Secretory C-mannosylatic	transfac. pro.M05752	4.47	0.4680	ZNF57 (directAnnotation).	2	DPY19L1; DPY19L2
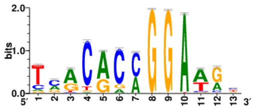	Secretory C-mannosylation	taipale. tf. pairs.ETV2. TBX21. TMACACMG-GAARN. CAP	4.40	0.4620	ETV2; TBX21 (directAnnotation).	3	DPY19L1; DPY19L2; DPY19L4

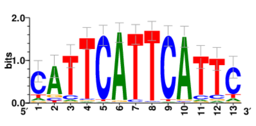	Secretory C-mannosylatic	metacluster. 145.10	4.37	0.4590	ZNF24 (directAnnotation).	2	DPY19L1; DPY19L4
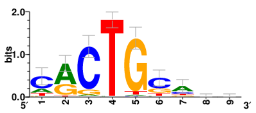	Secretory C-mannosylation	cisbp.M00152	4.37	0.4590	ZBTB3 (inferredBy.Orthology).	2	DPY19L1; DPY19L2
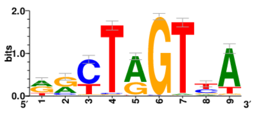	Secretory C-mannosylatic	hocomoco.HI HUMAN.H11M(	4.35	0.4570	HMBOX1 (directAnnotation).	3	DPY19L1; DPY19L3; DPY19L4
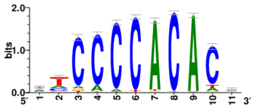	Secretory C-mannosylation	taipale. cyt. meth.FOXO3. NTCCCCACACN. eDBD	4.34	0.4560	FOXO3 (directAnnotation).	2	DPY19L1; DPY19L2
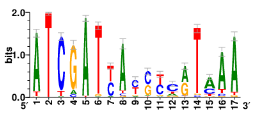	Secretory C-mannosylatic	taipale. tf. pairs.CUX1. HOXA13. ATCRATNN CRTAAA. CAP. repr	4.22	0.4450	CUX1; HOXA13 (directAnnotation).	2	DPY19L1; DPY19L3
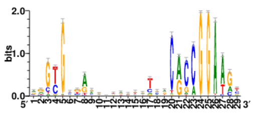	Secretory C-mannosylation	taipale. tf. pairs.ELK1. EOMES. RGGTGNGANNNNNNNNTNNCACCGGAAGY. CAP	4.22	0.4450	ELK1; EOMES (directAnnotation).	3	DPY19L1; DPY19L2; DPY19L4

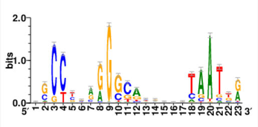	Secretory C-mannosylatic	taipale. tf. pairs.TFAP2 DLX3. NSCC-NNNRGGC CAP. repr	4.20	0.4440	DLX3; TFAP2C (directAn-notation).	3	DPY19L1; DPY19L2; DPY19L4
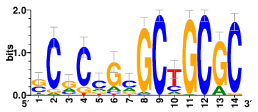	Secretory C-mannosylation	kznf.ZNF2. Im-beault2017. RP. ChIP-seq	4.18	0.4410	ZNF2 (directAn-notation).	4	DPY19L1; DPY19L2; DPY19L3; DPY19L4
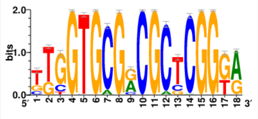	Secretory C-mannosylatic	transfac. pro.M06340	4.17	0.4410	ZNF790 (directAn-notation).	2	DPY19L1; DPY19L2
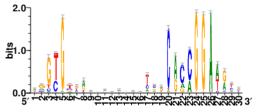	Secretory C-mannosylation	taipale. tf. pairs.ETV2. EOMES. NGGT-GTNNNNNNNNNNNNNNNC-CG-GAWNNN. CAP. repr	4.14	0.4380	EOMES; ETV2 (directAn-notation).	2	DPY19L1; DPY19L2
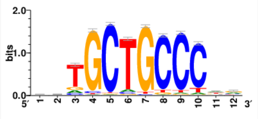	Secretory C-mannosylatic	jaspar.MA17	4.11	0.4350	ZNF549 (directAn-notation).	3	DPY19L1; DPY19L2; DPY19L4
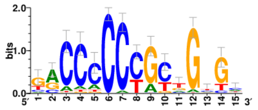	Secretory C-mannosylation	metacluster. 80.6	4.05	0.4290	ZIC4 (directAn-notation).	2	DPY19L1; DPY19L4
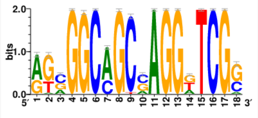	Secretory C-mannosylatic	transfac. pro.M06637	4.03	0.4270	ZNF577 (directAn-notation).	2	DPY19L3; DPY19L4

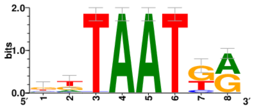	Secretory C-mannosylation	homer.GKTAATGR. Nkx6.1	4.02	0.4260	NKX6-1 (inferredBy.Orthology).	2	DPY19L1; DPY19L3
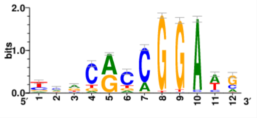	Secretory C-mannosylatic	taipale. tf. pairs.ETV5. EOMES. TNRCAC-CGGAWN. CAP	4.01	0.4260	EOMES; ETV5 (directAnnotation).	3	DPY19L1; DPY19L2; DPY19L4
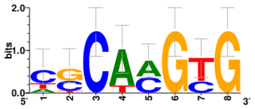	Secretory C-mannosylation	hdpi.MLX	4.00	0.4240	MLX (directAnnotation).	2	DPY19L1; DPY19L2
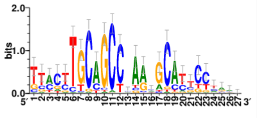	Secretory C-mannosylatic	kznf.ZNF582 Schmitges2016. RCADE	3.97	0.4220	ZNF582 (directAnnotation).	2	DPY19L2; DPY19L4
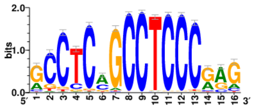	Secretory C-mannosylation	jaspar.MA1596.1	3.96	0.4210	ZNF460 (directAnnotation).	2	DPY19L1; DPY19L4
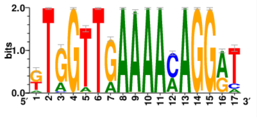	Secretory C-mannosylatic	transfac. pro.M05453	3.96	0.4210	ZBTB26 (directAnnotation).	2	DPY19L1; DPY19L2
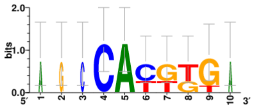	Secretory C-mannosylation	transfac. pro.M07350	3.93	0.4180	ARNTL (directAnnotation).	2	DPY19L1; DPY19L2

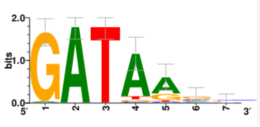	Secretory C-mannosylatic	transfac. pro.M01877	3.85	0.4110	GATA2 (directAnnotation).	3	DPY19L1; DPY19L2; DPY19L4
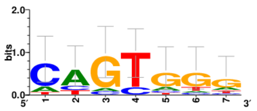	Secretory C-mannosylation	transfac. public.M00468	3.85	0.4110	KLF12 (directAnnotation).	2	DPY19L1; DPY19L2
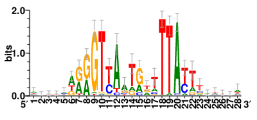	Secretory C-mannosylatic	tfdimers.MD	3.84	0.4100	HOXA13; NR1H4; NR1I2; NR1I3; RXRA (directAnnotation).	3	DPY19L1; DPY19L2; DPY19L4
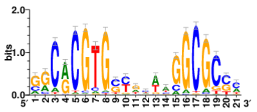	Secretory C-mannosylation	taipale. tf. pairs.E2F1. HES7. SRCRC-GYGSYNNNNS-GCGCSN. CAP. repr	3.84	0.4100	E2F1; HES7 (directAnnotation).	4	DPY19L1; DPY19L2; DPY19L3; DPY19L4
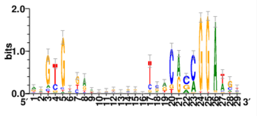	Secretory C-mannosylatic	taipale. tf. pairs.ETV5. EOMES. RNGT-GNNNNNNN CRCCG-GAWSN. CAP	3.83	0.4090	EOMES; ETV5 (directAnnotation).	3	DPY19L1; DPY19L2; DPY19L4
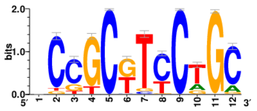	Secretory C-mannosylation	transfac. pro.M06444	3.82	0.4080	ZNF557 (directAnnotation).	2	DPY19L1; DPY19L4

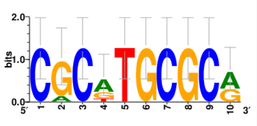	Secretory C-mannosylatic	transfac. pro.M00652	3.79	0.4050	NRF1 (directAnnotation).	2	DPY19L1; DPY19L2
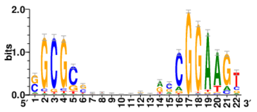	Secretory C-mannosylation	taipale. tf. pairs.E2F1. ELK1. SGCGC-NNNNNNNNNNNCG-GAAGN. CAP. repr	3.77	0.4040	E2F1; ELK1 (directAnnotation).	4	DPY19L1; DPY19L2; DPY19L3; DPY19L4
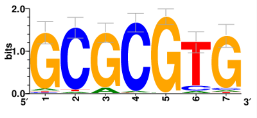	Secretory C-mannosylatic	metacluster. 198.5	3.77	0.4030	TCFL5; TCFL5 (directAnnotation).	2	DPY19L1; DPY19L2
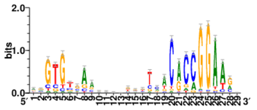	Secretory C-mannosylation	taipale. tf. pairs.ERF. TBX21. NRGTGT-NANNNNNNNNNNNNNC-CG-GAANN. CAP	3.75	0.4010	ERF; TBX21 (directAnnotation).	3	DPY19L1; DPY19L2; DPY19L4
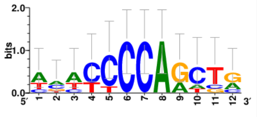	Secretory C-mannosylatic	transfac. pro.M03878	3.74	0.4010	HIVEP2 (directAnnotation).	2	DPY19L1; DPY19L2
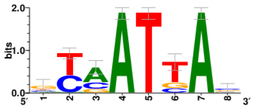	Secretory C-mannosylation	metacluster. 51.22	3.72	0.3990	MEOX1 (directAnnotation).	3	DPY19L1; DPY19L2; DPY19L3

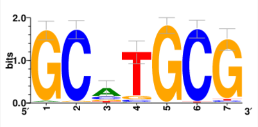	Secretory C-mannosylatic	metacluster. 55.3	3.68	0.3950	E2F6; EGR1; NRF1; NRF1; NRF1; NRF1; NRF1; NRF1; NRF1; NRF1; NRF1; NRF1; ZBTB2; ZSCAN10 (directAnnotation). NFE2L1; NRF1; NRF1; NRF1 (inferredBy.Ort	2	DPY19L1; DPY19L2
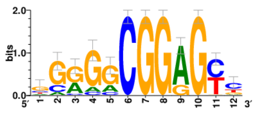	Secretory C-mannosylation	kznf.ZNF441. Imbeault2017. RP. RCADE	3.67	0.3940	ZNF441 (directAnnotation).	3	DPY19L1; DPY19L3; DPY19L4
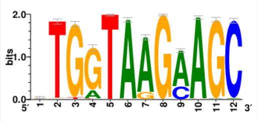	Secretory C-mannosylatic	transfac. pro.M06784	3.67	0.3940	ZNF780B (directAnnotation).	2	DPY19L1; DPY19L4
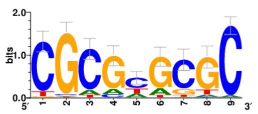	Secretory C-mannosylation	transfac. pro.M04866	3.64	0.3920	HDAC2 (directAnnotation).	2	DPY19L1; DPY19L2
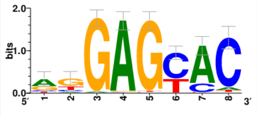	Secretory C-mannosylatic	c2h2. zfs.M0406	3.63	0.3910	ZNF691 (inferredBy.Ort	2	DPY19L1; DPY19L4

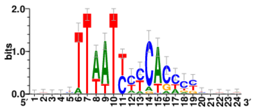	Secretory C-mannosylation	tfdimers.MD00423	3.63	0.3900	ARID3A; PAX4 (directAnnotation).	2	DPY19L1; DPY19L4
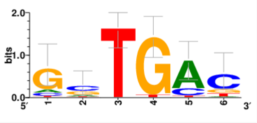	Secretory C-mannosylatic	transfac. pro.M01227	3.60	0.3880	MAFB (directAnnotation).	2	DPY19L1; DPY19L2
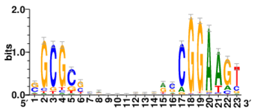	Secretory C-mannosylation	taipale. tf. pairs.E2F1. ELK1. SGCGC-NNNNNNNNNNNCG-GAAGN. CAP. repr	3.59	0.3870	E2F1; ELK1 (directAnnotation).	3	DPY19L1; DPY19L3; DPY19L4
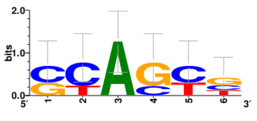	Secretory C-mannosylatic	transfac. pro.M03576	3.58	0.3860	UBP1 (inferredBy.Ort	2	DPY19L1; DPY19L2
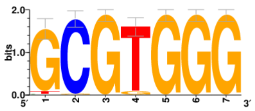	Secretory C-mannosylation	metacluster. 131.6	3.56	0.3840	EGR2; EGR4 (directAnnotation). EGR2; EGR3 (inferredBy.Orthology).	2	DPY19L1; DPY19L4
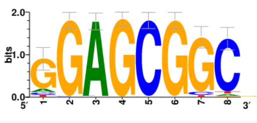	Secretory C-mannosylatic	metacluster. 144.5	3.50	0.3790	ZNF610; ZNF610; ZNF610 (directAnnotation).	3	DPY19L1; DPY19L3; DPY19L4

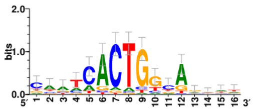	Secretory C-mannosylation	transfac. pro.M02929	3.42	0.3720	ZBTB3 (inferredBy.Orthology).	2	DPY19L1; DPY19L2
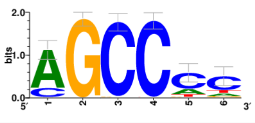	Secretory C-mannosylatio	metacluster. 195.1	3.41	0.3700	ZNF682 (directAnnotation).	4	DPY19L1; DPY19L2; DPY19L3; DPY19L4
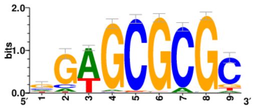	Secretory C-mannosylation	hocomoco.ZBTB14 HUMAN.H11MO.0.C	3.39	0.3680	ZBTB14 (directAnnotation).	2	DPY19L1; DPY19L2
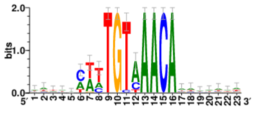	Secretory C-mannosylatio	tfdimers.MD	3.38	0.3670	FOXO1; SOX10 (directAnnotation).	2	DPY19L1; DPY19L3
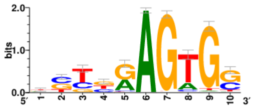	Secretory C-mannosylation	hocomoco.NKX25 MOUSE.H11MO.0.A	3.38	0.3670	NKX2-5 (inferredBy.Orthology).	2	DPY19L1; DPY19L2
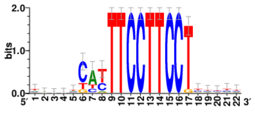	Secretory C-mannosylatio	tfdimers.MD	3.38	0.3670	ELF1; STAT1 (directAnnotation).	2	DPY19L1; DPY19L2
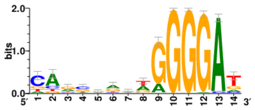	Secretory C-mannosylation	dbtfbs.MZF1. HEK293. ENCSR298QUH. merged. N1	3.37	0.3670	MZF1 (directAnnotation).	2	DPY19L1; DPY19L2

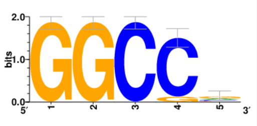	Secretory C- mannosylatic	metacluster. 7.13	3.36	0.3650	ZFX (directAn- notation).	3	DPY19L1; DPY19L3; DPY19L4
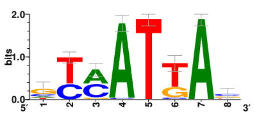	Secretory C- mannosylation	metacluster. 51.15	3.35	0.3640	HOXA5 (directAn- notation).	2	DPY19L2; DPY19L3
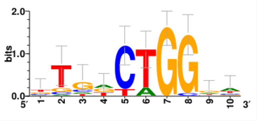	Secretory C- mannosylatic	transfac. pro.M03868	3.33	0.3630	TFCP2 (directAn- notation).	2	DPY19L1; DPY19L2
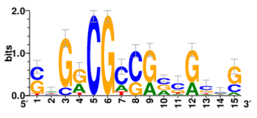	Secretory C- mannosylation	jaspar.MA0535.1	3.33	0.3620	SMAD9 (in- ferredBy.Orthology).	3	DPY19L1; DPY19L3; DPY19L4
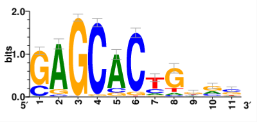	Secretory C- mannosylatic	transfac. pro.M12714	3.30	0.3600	ZNF691 (directAn- notation).	1	DPY19L1
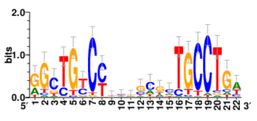	Secretory C- mannosylation	hocomoco.ZN335 HU- MAN.H11MO.0.A	3.29	0.3590	ZNF335 (directAn- notation).	3	DPY19L1; DPY19L2; DPY19L4
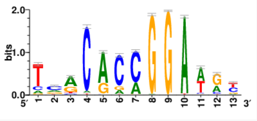	Secretory C- mannosylatic	taipale. tf. pairs.ETV2. EOMES. TCACAC- CG- GAWRY. CAP	3.26	0.3560	EOMES; ETV2 (directAn- notation).	2	DPY19L1; DPY19L2

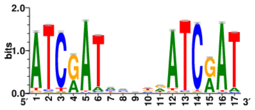	Secretory C-mannosylation	taipale.CUX1. DBD. ATCRATNNNNNAT-CRAT. repr	3.20	0.3510	CUX1 (directAn-notation).	2	DPY19L1; DPY19L4
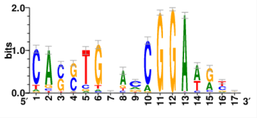	Secretory C-mannosylatic	taipale. tf. pairs.ETV2. TCF3. CASST-GNNCCG-GAWRYN. CAP. repr	3.16	0.3470	ETV2; TCF3 (directAn-notation).	2	DPY19L1; DPY19L2
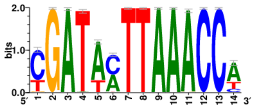	Secretory C-mannosylation	transfac. pro.M06936	3.16	0.3470	MAFF (in-ferredBy.Orthology).	2	DPY19L1; DPY19L2
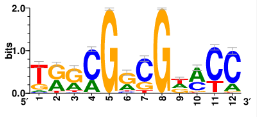	Secretory C-mannosylatic	transfac. pro.M05432	3.15	0.3460	ZSCAN18 (directAn-notation).	2	DPY19L1; DPY19L4
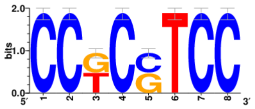	Secretory C-mannosylation	kznf.ZNF202. Im-beault2017. OM. MEME	3.14	0.3450	ZNF202 (directAn-notation).	2	DPY19L1; DPY19L4
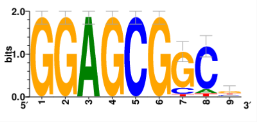	Secretory C-mannosylatic	kznf.ZNF610 Im-beault2017. OM. RCADE	3.12	0.3440	ZNF610 (directAn-notation).	3	DPY19L1; DPY19L3; DPY19L4

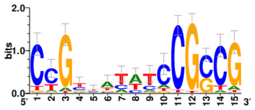	Secretory C-mannosylation	metacluster. 126.3	3.11	0.3420	LIN28B; LIN28B (directAn-notation).	2	DPY19L1; DPY19L4
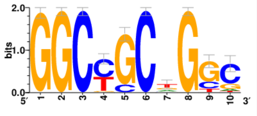	Secretory C-mannosylatic	dbtfbs.ZSCA HEK293. ENCSR768V merged. N1	3.08	0.3400	ZSCAN30 (directAn-notation).	3	DPY19L1; DPY19L3; DPY19L4
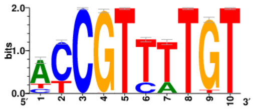	Secretory C-mannosylation	transfac. pro.M05299	3.06	0.3380	NKX2-6 (directAn-notation).	2	DPY19L1; DPY19L4
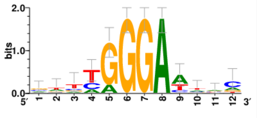	Secretory C-mannosylatic	transfac. pub-lic.M00087	3.04	0.3360	IKZF1 (directAn-notation).	3	DPY19L1; DPY19L2; DPY19L4
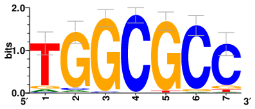	Secretory C-mannosylation	metacluster. 45.6	3.00	0.3330	E2F1; E2F1; E2F1; E2F1; E2F1; E2F2; E2F2; E2F2; E2F3; E2F3; E2F3; E2F4; E2F4 (directAn-notation). E2F2; E2F3 (in-ferredBy.Orthology).	2	DPY19L1; DPY19L4

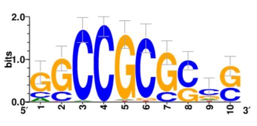	Secretory C-mannosylatio	transfac. pro.M01253	3.00	0.3330	CNOT3 (directAn-notation).	3	DPY19L1; DPY19L3; DPY19L4
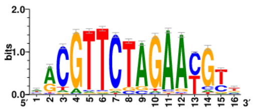	Elongation.bra	taipale cyt. meth.HSF5. NR-CGTTC-TA-GAAYGYN. eDBD. repr	6.01	0.2150	HSF5 (directAn-notation).	8	B3GALT2; B3GALT5; B3GNT2; B3GNT7; B4GALNT3; B4GALNT4; B4GALT2; GCNT2
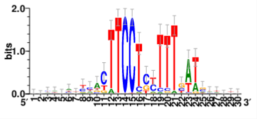	Elongation.b	tfdimers.MD	5.27	0.1920	ELF1; ELF2; ELK1; ELK4; ERF; ERG; ETS1; ETS2; ETV7; FLI1; TCF7L2 (directAn-notation).	7	B3GALT1; B3GALT5; B3GNT2; B4GALNT4; B4GALT1; B4GALT3; GCNT2
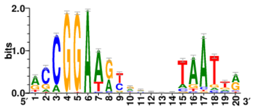	Elongation.bra	taipale tf. pairs.ETV2. DLX3. RSCG-GAANNNNNNNTAATKR. CAP. repr	5.16	0.1890	DLX3; ETV2 (directAn-notation).	4	B3GALT2; B4GALNT3; B4GALNT4; B4GALT1
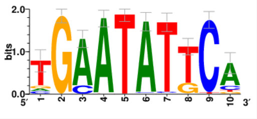	Elongation.b	metacluster. 62.9	5.00	0.1840	POU2F2 (directAn-notation).	4	B3GALT1; B3GNT3; B4GALNT4; B4GALT4

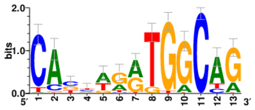	Elongation.branching	kzdf.CTCF. Transfac.6. Transfac	4.85	0.1790	CTCF (directAnnotation).	4	B3GNT2; B4GALNT3; B4GALNT4; GCNT2
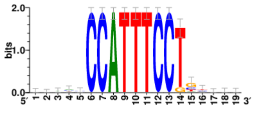	Elongation.b	tfdimers.MD	4.60	0.1710	GABPA; YY2 (directAnnotation).	4	B3GNT2; B4GALNT3; B4GALNT4; B4GALT1
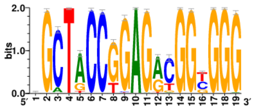	Elongation.branching	transfac. pro.M05429	4.59	0.1710	ZNF521 (directAnnotation).	5	B3GALT1; B3GALT5; B3GNT2; B3GNT4; B4GALNT3
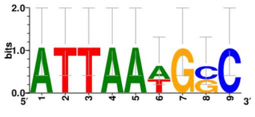	Elongation.b	hdpi.TBPL1	4.53	0.1690	TBPL1 (directAnnotation).	4	B3GNT2; B3GNT3; B4GALNT4; B4GALT2
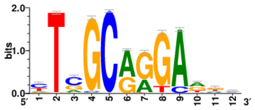	Elongation.branching	hocomoco.KAISO HUMAN.H11MO.2.A	4.41	0.1650	ZBTB33 (directAnnotation).	4	B3GALT5; B4GALNT3; B4GALT1; GCNT2
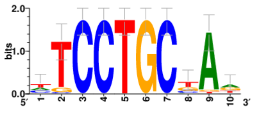	Elongation.b	transfac. pro.M01119	4.23	0.1600	ZBTB33 (directAnnotation).	4	B3GALT5; B4GALNT3; B4GALT1; GCNT2
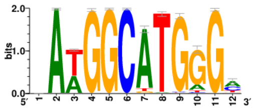	Elongation.branching	transfac. pro.M06755	4.21	0.1590	ZFPM1 (inferredBy.Orthology).	4	B3GALT1; B3GNT3; B4GALNT3; B4GALT1

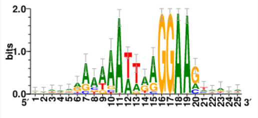	Elongation.b	tfdimers.MD	4.06	0.1550	GABPA; PAX4 (directAnnotation).	4	B3GNT2; B3GNT7; GCNT2; GCNT7
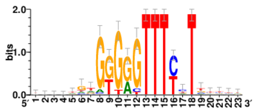	Elongation.branching	tfdimers.MD004[illegible]	[illegible]	0.1530	E2F6; OVOL2 (directAnnotation).	3	B3GNT2; B4GALNT3; B4GALT4
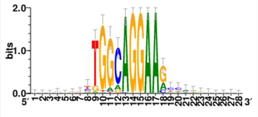	Elongation.b	tfdimers.MD	4.00	0.1530	ELF1; NFIA; NFIC (directAnnotation).	3	B3GNT8; B4GALNT3; B4GALNT4
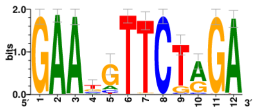	Elongation.branching	metacluster. 56.3	3.85	0.1480	HSF2 (directAnnotation).	7	B3GALT5; B3GNT2; B3GNT3; B3GNT7; B4GALNT3; B4GALT2; GCNT2
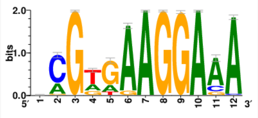	Elongation.b	transfac. pro.M06292	3.85	0.1480	ZNF766 (directAnnotation).	4	B3GALT1; B4GALNT3; B4GALT1; B4GALT3
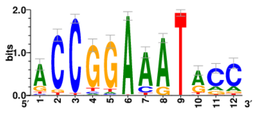	Elongation.branching	taipale. tf. pairs.TEAD4. ETV4. RCCG-GAAA-TRCC. CAP	3.84	0.1480	ETV4; TEAD4 (directAnnotation).	4	B3GALT2; B3GNT3; B4GALT1; GCNT2

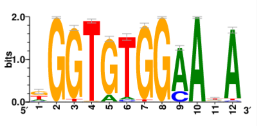	Elongation.b	transfac. pro.M06016	3.84	0.1480	ZNF729 (directAn- notation).	5	B3GALT1; B3GNT3; B4GALNT4; B4GALT3; GCNT2
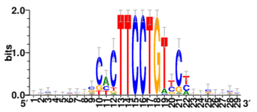	Elongation.bra[illegible]	tfdimers.MD00[illegible]	[illegible]	0.1450	NR3C1; STAT1 (directAn- notation).	5	B3GALT1; B3GNT2; B4GALNT3; B4GALNT4; B4GALT3
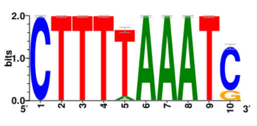	Elongation.b	transfac. pro.M05390	3.73	0.1440	TBPL2 (directAn- notation).	3	B3GALT2; B3GALT5; B4GALT3
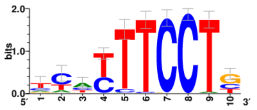	Elongation.bra[illegible]	transfac. pro.M04864	3.72	0.1440	GATA2 (directAn- notation).	3	B4GALNT3; B4GALNT4; B4GALT1
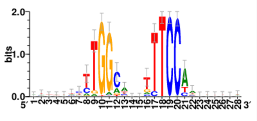	Elongation.b	tfdimers.MD	3.69	0.1430	NFATC1; NFIA; NFIC (directAn- notation).	3	B3GALT2; B4GALNT4; B4GALT4
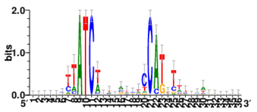	Elongation.bra[illegible]	tfdimers.MD00[illegible]	[illegible]	0.1430	GATA1; GATA2; GATA3; GATA4; GATA5; GATA6; YY1 (directAn- notation).	3	B3GNT7; B4GALNT4; B4GALT1

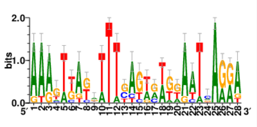	Elongation.b	kznf.ZNF334 Im- beault2017. OM. MEME	3.66	0.1420	ZNF334 (directAn- notation).	4	B3GALT5; B3GNT7; B4GALNT3; GCNT2
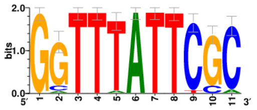	Elongation.bra[illegible]	metacluster. 27.29	3.65	0.1420	ZNF583 (directAn- notation). ZNF583 (in- ferredBy.Orthology).	4	B3GALT1; B3GNT2; GCNT2; GCNT7
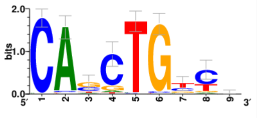	Elongation.b	transfac. pro.M02100	3.65	0.1420	MYOD1 (directAn- notation).	4	B3GALT2; B3GALT5; B4GALNT4; B4GALT1
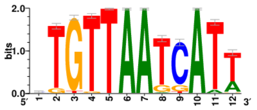	Elongation.bra[illegible]	transfac. pro.M06090	3.63	0.1410	ZNF12 (directAn- notation).	4	B3GALT2; B3GALT5; B3GNT2; GCNT2
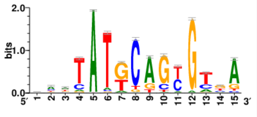	Elongation.b	taipale. tf. pairs.POU2F EOMES. NNNTAT- GCAGYGTI CAP. repr	3.61	0.1410	EOMES; POU2F1 (directAn- notation).	10	B3GALT1; B3GALT2; B3GALT5; B3GNT2; B3GNT7; B4GALT1; B4GALT2; B4GALT3; GCNT2; GCNT7

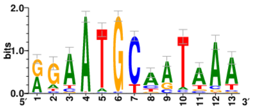	Elongation.branching	tandem tf. pairs.TEAD4. HOXB13. RRAATG-CAR-TAAA. CAP. repr	3.60	0.1400	HOXB13; TEAD4 (directAnnotation).	3	B3GNT2; B4GALNT4; B4GALT4

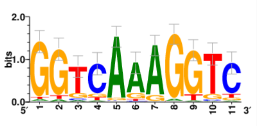	Elongation.b	metacluster. 120.1	3.57	0.1390	BCL11B; HNF4A; HNF4A; HNF4A; HNF4G; NR1H2; NR1H2; NR1H4; NR2C1; NR2C2; NR2C2; NR2C2; NR2C2; NR2C2; NR2E3; NR2F1; NR2F1; NR2F1; NR2F1; NR2F1; NR2F6; NR2F6; NR2F6; NR2F6; NR2F6; PPARA; PPARA; PPARA; PPARD; PPARD; PPARD; PPARD; PPARG; PPARG; PPARG; PPARG; PPARG; RXRA; RXRA; RXRA; RXRA; RXRA; RXRA; RXRA; RXRB; RXRB; RXRB; RXRG; RXRG; RXRG; RXRG; RXRG; THRB; THRB (directAnnotation). HNF4A;	4	B4GALNT3; B4GALNT4; B4GALT1; B4GALT4

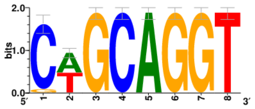	Elongation.bra	kznf.CTCF. Transfac.8. Transfac	3.54	0.1380	CTCF (directAnnotation).	3	B3GALT2; B3GALT5; B3GNT9
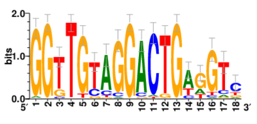	Elongation.b	kznf.ZNF181 Imbeault2017. OM. RCADE	3.53	0.1380	ZNF181 (directAnnotation).	3	B3GALT5; B3GNT7; B4GALT4
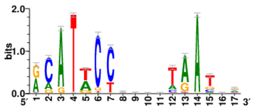	Elongation.bra	taipale. tf. pairs.TEAD4. DLX3. RCATTCC-NNNNTAATKR. CAP	3.50	0.1370	DLX3; TEAD4 (directAnnotation).	3	B3GNT7; B4GALNT3; B4GALNT4
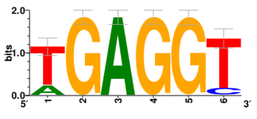	Elongation.b	kznf.ZNF10. Imbeault2017. RP. RCADE	3.48	0.1370	ZNF10 (directAnnotation).	2	B4GALNT3; B4GALNT4
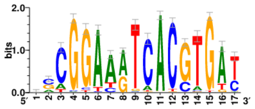	Elongation.bra	jaspar.MA1939	3.45	0.1360	SREBF2 (directAnnotation).	3	B3GALT1; B3GNT2; B3GNT7
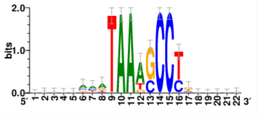	Elongation.b	tfdimers.MD	3.45	0.1360	HOXA13; TFAP2C (directAnnotation).	4	B3GALT5; B3GNT2; B4GALT1; B4GALT4

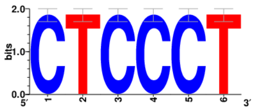	Elongation.branching	kznf.ZNF169. Imbeault2017. RP. RCADE	3.44	0.1350	ZNF169 (directAnnotation).	6	B3GNT2; B3GNT7; B3GNT8; B4GALNT3; B4GALNT4; B4GALT1
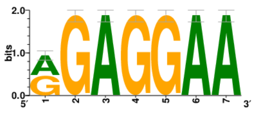	Elongation.b	kznf.ZNF695 Imbeault2017. OM. MEME	3.43	0.1350	ZNF695 (directAnnotation).	7	B3GALT1; B3GNT3; B3GNT8; B4GALNT3; B4GALNT4; B4GALT1; GCNT2
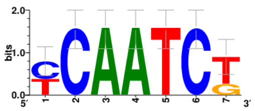	Elongation.branching	transfac. pro.M01108	3.43	0.1350	HOXA7 (directAnnotation).	3	B3GALT1; B3GNT2; B3GNT9
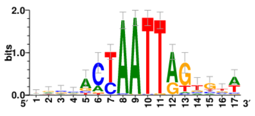	Elongation.b	metacluster. 9.7	3.41	0.1350	GBX1 (directAnnotation).	2	B3GALT5; B3GNT2
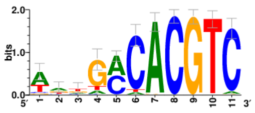	Elongation.branching	metacluster. 139.7	3.35	0.1330	XBP1 (directAnnotation). XBP1 (inferredBy.Orthology).	3	B3GNT2; B4GALNT3; GCNT2
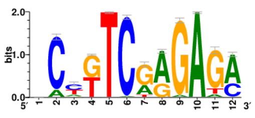	Elongation.b	transfac. pro.M05721	3.34	0.1320	ZNF235 (directAnnotation).	6	B3GALT2; B3GNT2; B4GALNT4; B4GALT3; GCNT2; GCNT7

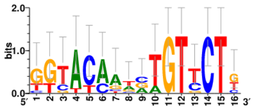	Elongation.branching	transfac. pub-lic.M00205	3.31	0.1310	NR3C1 (directAn-notation).	3	B3GALT1; B4GALNT3; GCNT2
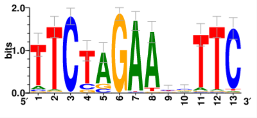	Elongation.b	metacluster. 56.4	3.26	0.1300	HSF1; HSF2; HSF2; HSF4 (directAn-notation).	5	B3GNT2; B3GNT3; B3GNT7; B4GALNT3; B4GALT2
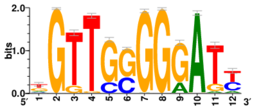	Elongation.branching	transfac. pro.M05637	3.20	0.1280	ZNF189 (directAn-notation).	4	B3GALT2; B3GNT3; B4GALNT4; GCNT2
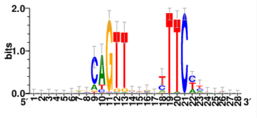	Elongation.b	tfdimers.MD	3.19	0.1280	MYB; STAT6 (directAn-notation).	10	B3GALT1; B3GALT2; B3GALT5; B3GNT2; B3GNT3; B3GNT7; B4GALNT3; B4GALNT4; B4GALT2; GCNT2
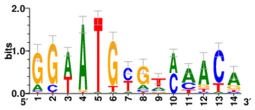	Elongation.branching	taipale. tf. pairs.TEAD4. FOXI1. GGWAT-GYGT-MAACA. CAP. repr	3.18	0.1270	FOXI1; TEAD4 (directAn-notation).	6	B4GALNT3; B4GALNT4; B4GALT2; B4GALT3; B4GALT4; GCNT2

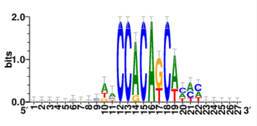	Elongation.b	tfdimers.MD	3.17	0.1270	DBP (directAnnotation).	5	B3GALT1; B3GNT2; B4GALNT3; B4GALT3; GCNT7
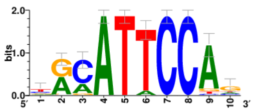	Elongation.branching	homer TRCATTCCAG TEAD3	3.17	0.1270	TEAD3 (directAnnotation).	3	B3GNT7; B4GALNT4; B4GALT1
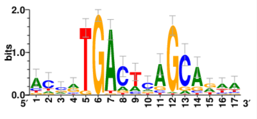	Elongation.b	metacluster. 22.39	3.16	0.1270	NFE2L2 (directAnnotation).	6	B3GALT1; B3GALT2; B3GNT9; B4GALNT3; B4GALNT4; GCNT2
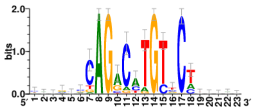	Elongation.branching	tfdimers.MD00524	3.14	0.1260	NR3C1; SMAD4 (directAnnotation).	2	B3GALT2; B3GNT9
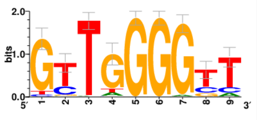	Elongation.b	kznf.ZNF749 Imbeault2017. RP. RCADE	3.13	0.1260	ZNF749 (directAnnotation).	3	B4GALNT3; B4GALNT4; B4GALT2
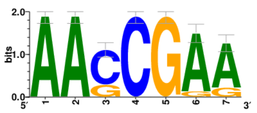	Elongation.branching	transfac. pro.M04887	3.09	0.1240	TCF12 (directAnnotation).	3	B3GNT4; B4GALNT3; B4GALT3
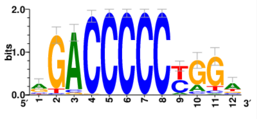	Elongation.b	flyfactorsurv F3-5-2. SOLEXA. 5	3.06	0.1240	GLIS2 (inferredBy.Ort	3	B3GALT2; B4GALT3; GCNT2

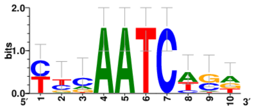	Elongation.branching	transfac. pro.M03825	3.05	0.1230	GFI1B (directAnnotation).	4	B3GALT5; B4GALT2; B4GALT3; B4GALT4
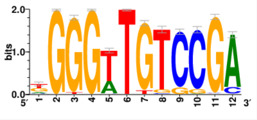	Elongation.b	transfac. pro.M06395	3.02	0.1220	ZNF534 (directAnnotation).	3	B3GALT2; B4GALNT3; B4GALT3
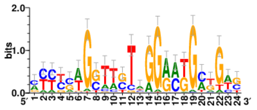	Elongation.branching	kz[illegible]fZNF181. Imbeault2017. RP. RCADE	3.01	0.1220	ZNF181 (directAnnotation).	4	B3GNT3; B3GNT8; B4GALNT4; B4GALT4
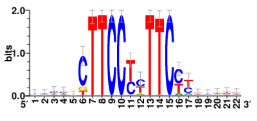	Capping	tfdimers.MD[illegible]	4.99	0.1470	GABPA; STAT6 (directAnnotation).	6	B3GAT2; B4GALNT2; FUT7; ST3GAL5; ST3GAL6; ST6GALNAC1
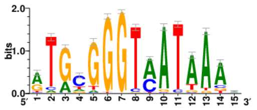	Capping	taipale. tf. pairs.GCM1. HOXB13. RT-GCGGGTAATAAAN. CAP	4.84	0.1430	GCM1; HOXB13 (directAnnotation).	5	A4GNT; B3GAT2; B4GALNT2; FUT5; ST3GAL4
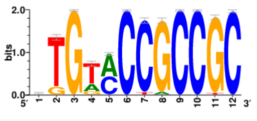	Capping	transfac. pro.M06163	4.63	0.1390	ZNF121 (directAnnotation).	7	B3GAT1; ST3GAL5; ST6GAL2; ST6GALNAC2; ST6GALNAC3; ST8SIA2; ST8SIA6

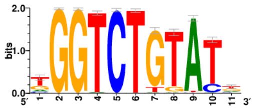	Capping	transfac. pro.M06434	4.49	0.1350	ZNF787 (inferredBy.Orthology).	9	B4GALNT2; FUT4; ST3GAL2; ST3GAL6; ST6GAL2; ST6GALNAC2; ST8SIA1; ST8SIA2; ST8SIA5
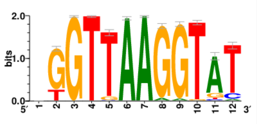	Capping	transfac. pro.M05722	4.21	0.1290	ZNF8 (directAnnotation).	6	A4GNT; B3GAT2; ST3GAL6; ST6GAL2; ST6GALNAC3; ST8SIA6
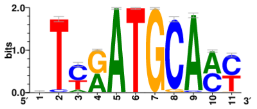	Capping	transfac. pro.M06845	4.06	0.1260	ZBTB38 (directAnnotation).	12	B3GAT2; FUT11; FUT5; FUT9; ST3GAL1; ST3GAL5; ST3GAL6; ST6GAL1; ST6GALNAC6; ST8SIA4; ST8SIA5; ST8SIA6
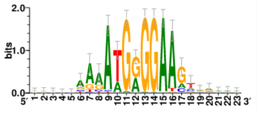	Capping	tfdimers.MD	3.96	0.1230	ETS1; YY1 (directAnnotation).	8	A4GNT; B3GAT1; B4GALNT2; ST3GAL1; ST3GAL5; ST6GALNAC1; ST6GALNAC3; ST8SIA3

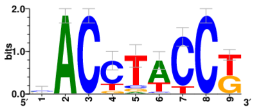	Capping	kznf.ZNF784. Schmit-ges2016. RCADE	3.91	0.1220	ZNF784 (directAn-notation).	3	B3GAT1; FUT5; FUT6
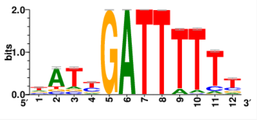	Capping	hocomoco.O[illegible] HU-MAN.H11M[illegible]	3.91	0.1220	ONECUT3 (directAn-notation).	7	B3GAT1; ST3GAL1; ST3GAL6; ST6GAL2; ST6GALNAC3; ST8SIA1; ST8SIA6
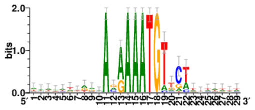	Capping	tfdimers.MD00[illegible]	3.89	0.1220	E2F6; NR3C1 (directAn-notation).	7	FUT10; FUT4; ST3GAL2; ST3GAL4; ST6GAL2; ST6GALNAC2; ST6GALNAC3
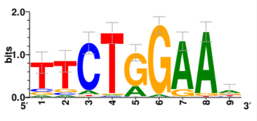	Capping	hocomoco.H[illegible] HU-MAN.H11M[illegible]	3.86	0.1210	HSF1 (directAn-notation).	6	B3GAT1; FUT1; FUT5; FUT6; ST3GAL3; ST8SIA2
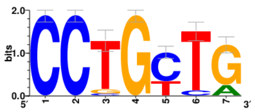	Capping	transfac. pro.M04912	3.79	0.1190	FOXA1 (directAn-notation).	7	FUT3; FUT5; FUT6; ST3GAL4; ST6GAL2; ST6GALNAC5; ST8SIA5

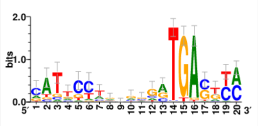	Capping	cisbp.M0783[illegible]	3.72	0.1180	JUN (directAn-notation).	6	B3GAT1; B3GAT2; B4GALNT2; ST3GAL5; ST3GAL6; ST6GALNAC5
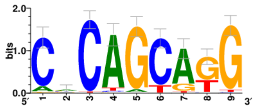	Capping	metacluster. 80.16	3.62	0.1160	ZIC1 (directAn-notation). ZIC2 (in-ferredBy.Orthology).	5	FUT3; FUT5; FUT6; ST3GAL5; ST6GALNAC6
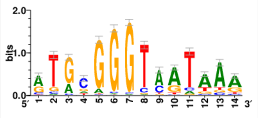	Capping	taipale. tf. pairs.GCM1. HOXA13. RKRNGGG[illegible] NATAAA. CAP	3.60	0.1150	GCM1; HOXA13 (directAn-notation).	8	A4GNT; B3GAT2; B4GALNT2; FUT5; ST3GAL4; ST6GALNAC1; ST6GALNAC5; ST8SIA6
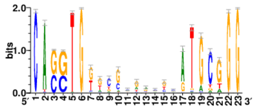	Capping	taipale. tf. pairs.GCM1. FIGLA. CAGCT-GNNNNNNNNNNNT-GCGGG. CAP	3.51	0.1130	FIGLA; GCM1 (directAn-notation).	12	A4GNT; B3GAT1; B3GAT2; FUT6; FUT9; ST3GAL2; ST3GAL5; ST3GAL6; ST6GAL1; ST6GAL2; ST6GALNAC1; ST8SIA2

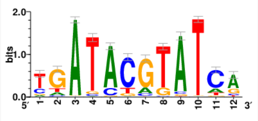	Capping	nitta.so. TATCGC20N KG. NGAT- ACG- TATCN. m1. c3	3.50	0.1130	SIX1 (in- ferredBy.Ort	11	ABO; FUT2; FUT9; ST3GAL4; ST6GAL1; ST6GAL2; ST6GALNAC1; ST6GALNAC3; ST6GALNAC4; ST8SIA2; ST8SIA4
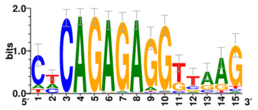	Capping	metacluster. 168.5	3.47	0.1120	ZNF768; ZNF768 (directAn- notation).	16	A4GNT; B3GAT1; B3GAT2; FUT4; FUT5; FUT6; FUT7; ST3GAL2; ST3GAL4; ST3GAL5; ST3GAL6; ST6GAL2; ST6GALNAC1; ST6GALNAC4; ST6GALNAC6; ST8SIA1
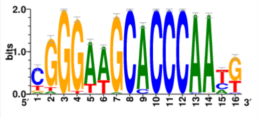	Capping	transfac. pro.M06543	3.45	0.1120	ZNF418 (directAn- notation).	4	FUT9; ST3GAL4; ST6GALNAC6; ST8SIA2
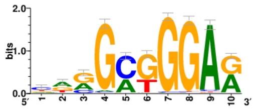	Capping	transfac. pro.M12598	3.43	0.1110	E2F3 (directAn- notation).	4	B3GAT1; FUT9; ST3GAL6; ST6GAL2

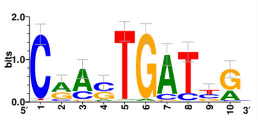	Capping	metacluster.33.3	3.41	0.1110	ZNF134; ZNF174 (directAnnotation).	3	FUT3; FUT5; FUT6
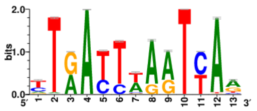	Capping	jaspar.MA0884.1	3.34	0.1090	DUXA (directAnnotation).	4	ABO; ST6GAL1; ST8SIA1; ST8SIA3
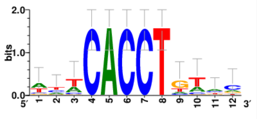	Capping	transfac.public.M00413	3.31	0.1080	ZEB1 (directAnnotation).	5	B3GAT1; B3GAT2; FUT2; FUT6; FUT9
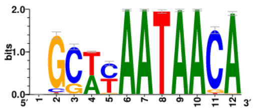	Capping	transfac.pro.M05699	3.30	0.1080	ZNF816 (directAnnotation).	7	ST3GAL4; ST6GAL1; ST6GALNAC3; ST6GALNAC5; ST6GALNAC6; ST8SIA2; ST8SIA3
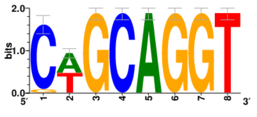	Capping	kznf.CTCF.Transfac.8.Transfac	3.26	0.1070	CTCF (directAnnotation).	10	B3GAT2; FUT1; FUT3; FUT4; FUT5; FUT6; FUT9; ST3GAL2; ST3GAL5; ST6GAL2

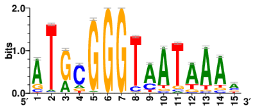	Capping	taipale. tf. pairs.GCM2. HOXA13. RTR- CGGGTAATAAAN. CAP. repr	3.26	0.1070	GCM2; HOXA13 (directAn- notation).	4	A4GNT; B4GALNT2; ST3GAL4; ST8SIA6
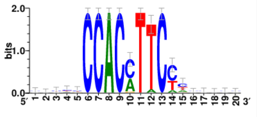	Capping	tfdimers.MD[illegible]	3.22	0.1060	STAT6; ZNF354C (directAn- notation).	4	B3GAT1; B4GALNT2; FUT9; ST6GAL2
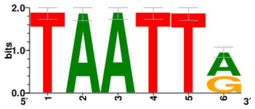	Capping	metacluster. 20.7	3.21	0.1060	EN2; EN2 (directAn- notation).	6	B3GAT2; ST3GAL4; ST3GAL6; ST6GAL2; ST8SIA3; ST8SIA6
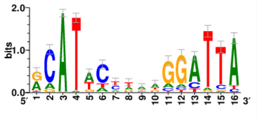	Capping	taipale. tf. pairs.TEAD4 PITX1. RCATWC- NNNNG- GATTA. CAP. repr	3.19	0.1060	PITX1; TEAD4 (directAn- notation).	4	B3GAT1; ST3GAL4; ST6GALNAC6; ST8SIA2
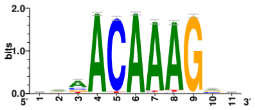	Capping	jaspar.MA0442[illegible]	3.12	0.1040	SOX10 (directAn- notation).	5	A4GNT; ST3GAL2; ST3GAL4; ST6GAL1; ST8SIA4

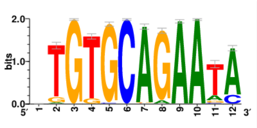	Capping	transfac. pro.M06577	3.12	0.1040	ZNF594 (directAn- notation).	9	B3GAT2; B4GALNT2; FUT10; FUT4; ST3GAL1; ST3GAL5; ST3GAL6; ST6GALNAC5; ST8SIA6
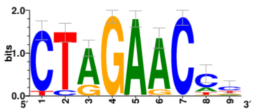	Capping	kznf.ZBTB12. Schmit- ges2016. RCADE	3.11	0.1040	ZBTB12 (directAn- notation).	6	B3GAT1; FUT1; FUT10; ST6GALNAC5; ST8SIA2; ST8SIA5
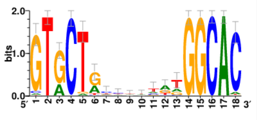	Capping	metacluster. 122.2	3.09	0.1030	ZBTB43; ZBTB43 (directAn- notation).	5	FUT4; ST3GAL4; ST6GAL1; ST6GAL2; ST8SIA5
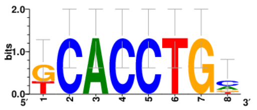	Capping	transfac. pro.M03565	3.09	0.1030	SNAI2 (directAn- notation).	5	FUT3; FUT5; FUT6; FUT9; ST6GAL2
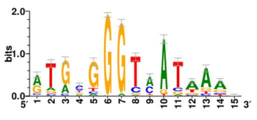	Capping	taipale. tf. pairs.GCM1. HOXB13. RTRNGGGT CAP	3.09	0.1030	GCM1; HOXB13 (directAn- notation).	11	A4GNT; B3GAT2; B4GALNT2; FUT3; FUT5; ST3GAL4; ST3GAL6; ST6GALNAC3; ST6GALNAC5; ST6GALNAC6; ST8SIA6

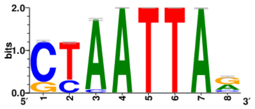	Capping	taipale. cyt. meth.LHX9. CYAAT-TAR. eDBD	3.07	0.1030	LHX9 (directAn-notation).	7	FUT1; ST3GAL4; ST3GAL6; ST6GAL2; ST8SIA1; ST8SIA3; ST8SIA6
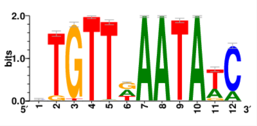	Capping	transfac. pro.M05738	3.07	0.1030	ZNF470 (directAn-notation).	4	B3GAT1; FUT9; ST3GAL5; ST6GALNAC6
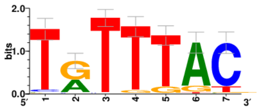	Capping	metacluster. 134.2	3.05	0.1020	FOXA1; FOXB1; FOXJ2; FOXJ2 (directAn-notation). FOXA2 (in-ferredBy.Orthology).	7	ABO; FUT2; ST3GAL3; ST6GAL1; ST8SIA1; ST8SIA4; ST8SIA5
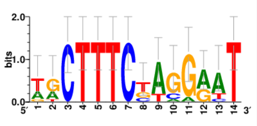	Capping	transfac. pro.M01185	3.04	0.1020	BCL6 (directAn-notation).	3	ST6GAL2; ST6GALNAC3; ST8SIA2
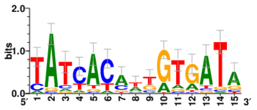	Capping	metacluster. 109.1	3.03	0.1020	NFX1 (in-ferredBy.Orthology).	6	B3GAT2; ST3GAL4; ST6GAL1; ST6GAL2; ST6GALNAC4; ST6GALNAC6
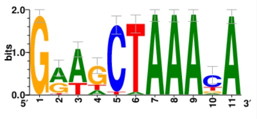	Capping	metacluster. 134.5	3.02	0.1020	ZNF184 (directAn-notation).	5	FUT3; FUT4; FUT9; ST3GAL1; ST3GAL4

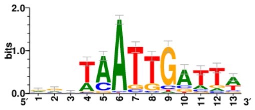	Capping	hocomoco.PAX3 MOUSE.H11MO.0.D	3.02	0.1020	PAX3 (inferredBy.Orthology).	6	B3GAT1; ST3GAL1; ST3GAL4; ST6GAL1; ST6GAL2; ST6GALNAC2
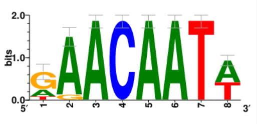	Sulfotransfer	cisbp.M0021	3.76	0.1430	SOX13 (inferredBy.Ort	9	CHST1; CHST8; GLCE; HS3ST2; HS3ST5; HS3ST6; HS6ST2; TPST1; TPST2
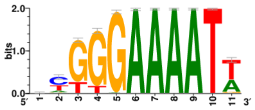	Sulfotransferases	transfac. pro.M06569	3.65	0.1400	ZNF775 (directAnnotation).	7	CHST2; CHST3; CHST8; CHST9; DSEL; GAL3ST1; HS3ST3A1
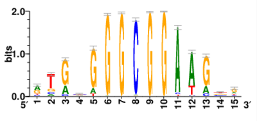	Sulfotransfer	taipale. tf. pairs.GCM1. ELK1. RT-GCGGGCG-GAAGTN. CAP. 1	3.44	0.1340	ELK1; GCM1 (directAnnotation).	7	CHST7; CHST9; DSE; DSEL; GLCE; NDST3; UST
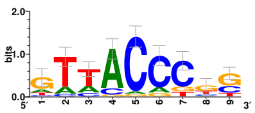	Sulfotransferases	metacluster. 155.11	3.44	0.1340	SPZ1 (directAnnotation).	7	CHST9; DSE; GLCE; HS3ST6; HS6ST3; NDST3; UST

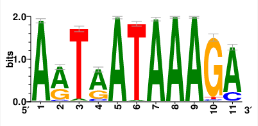	Sulfotransfer	transfac. pro.M05411	3.44	0.1340	ZNF784 (in- ferredBy.Ort	9	CHST5; DSE; DSEL; GAL3ST2; GLCE; HS3ST3A1; HS3ST4; HS3ST5; HS6ST3
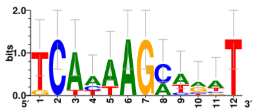	Sulfotransferasesdpi.NME1		3.34	0.1320	NME1 (directAn- notation).	13	CHST10; CHST13; CHST14; CHST15; CHST8; DSE; GAL3ST2; GLCE; HS3ST3A1; HS6ST1; HS6ST3; TPST1; UST
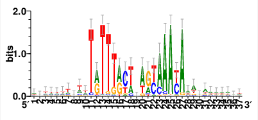	Sulfotransfer	tfdimers.MD	3.25	0.1290	FOXA1; FOXA2; FOXA3 (directAn- notation).	8	CHST1; CHST10; CHST12; CHST15; CHST8; GAL3ST2; GAL3ST4; HS6ST1
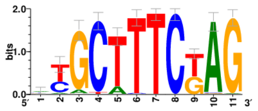	Sulfotransferasesmetacluster. 93.4		3.24	0.1290	BCL6B (directAn- notation).	6	CHST4; CHST8; DSEL; HS3ST6; HS6ST1; HS6ST3

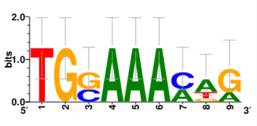	Sulfotransfer	hdpi.TFE3	3.22	0.1280	TFE3 (directAnnotation).	6	CHST15; CHST2; CHST8; GAL3ST2; GLCE; HS3ST3A1
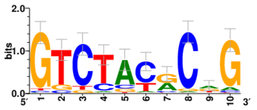	Sulfotransferase	metacluster. 172.26	3.19	0.1280	ZSCAN29 (directAnnotation).	7	CHST8; CHST9; DSEL; GLCE; HS3ST6; HS6ST3; NDST3
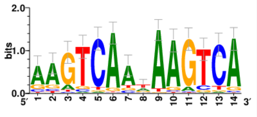	Sulfotransfer	metacluster. 140.7	3.18	0.1270	NR2E1; NR2E3 (directAnnotation). NR2E1; NR2E1; NR2E1 (inferredBy.Ort	5	CHST14; CHST8; DSE; GAL3ST2; HS6ST1
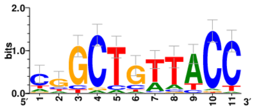	Sulfotransferase	metacluster. 79.30	3.16	0.1270	ZNF341; ZNF589 (directAnnotation).	8	CHST6; CHST9; DSE; GLCE; HS3ST6; HS6ST3; NDST3; UST

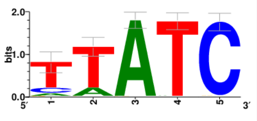	Sulfotransfer	metacluster. 117.2	3.13	0.1260	GATA3; GATA3; GATA3; GATA5; GATA5; GATA5; GATA5; GATA6; GATA6 (directAn-notation).	9	CHST12; CHST15; CHST8; DSEL; GAL3ST1; HS3ST1; HS6ST1; HS6ST2; NDST4
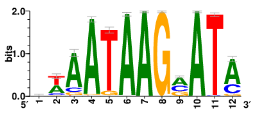	Sulfotransferasesransfac.	pro.M06357	3.08	0.1250	ZNF629 (in-ferredBy.Orthology).	7	CHST11; CHST13; CHST14; CHST3; CHST7; DSE; NDST4
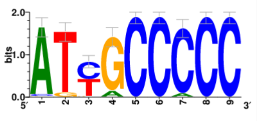	Sulfotransfer	metacluster. 159.8	3.08	0.1250	ZNF770 (directAn-notation). ZNF770 (in-ferredBy.Ort	6	CHST2; CHST9; GLCE; HS3ST4; HS6ST3; NDST3
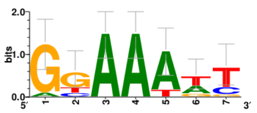	Sulfotransferasesransfac.	pro.M00750	3.02	0.1230	HMGA1; HMGA2 (directAn-notation).	7	CHST15; CHST2; CHST8; CHST9; GAL3ST2; HS3ST3B1; NDST4
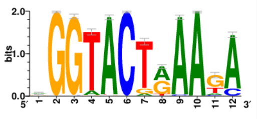	Sulfotransfer	transfac. pro.M06174	3.00	0.1230	ZNF93 (directAn-notation).	5	CHST8; GLCE; HS6ST1; NDST3; UST

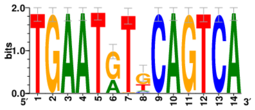	Hydrolase	metacluster. 65.6	6.87	0.3510	SOX8 (directAnnotation).	3	FUCA2; NEU3; NEU4
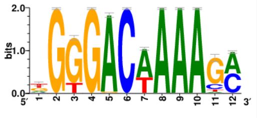	Hydrolase	transfac. pro.M06362	6.65	0.3410	ZNF730 (directAnnotation).	3	FUCA1; FUCA2; NEU2
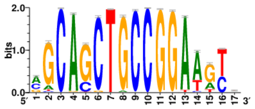	Hydrolase	taipale. tf. pairs.ETV2. NHLH1. NGCAGCTGCCGGAWRYN. CAP. repr	6.14	0.3160	ETV2; NHLH1 (directAnnotation).	4	FUCA1; FUCA2; NEU2; NEU3
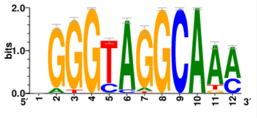	Hydrolase	transfac. pro.M06335	5.87	0.3030	ZNF343 (directAnnotation).	2	FUCA1; FUCA2
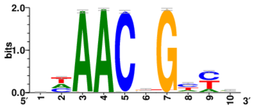	Hydrolase	swissregulon.hs.MYBL2	5.86	0.3030	MYBL2 (directAnnotation).	2	NEU3; NEU4
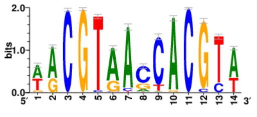	Hydrolase	taipale.GMEDBD. WRCGTAACCACGYN. repr	5.83	0.3020	GMEB2 (directAnnotation).	3	FUCA2; NEU1; NEU4
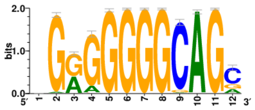	Hydrolase	transfac. pro.M05995	5.77	0.2990	ZNF180 (directAnnotation).	2	NEU3; NEU4

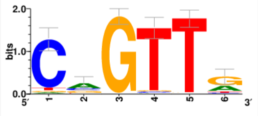	Hydrolase	metacluster. 113.6	5.77	0.2990	MYB; MYB; MYB; MYB; MYBL1; MYBL1; MYBL2 (directAn- notation). MYB; MYB; MYB (in- ferredBy.Ort	2	NEU3; NEU4
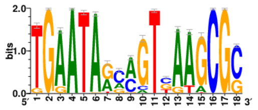	Hydrolase	transfac. pro.M06500	5.73	0.2970	ZNF614 (directAn- notation).	3	FUCA2; NEU2; NEU3
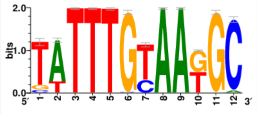	Hydrolase	transfac. pro.M06993	5.63	0.2920	NFE2L3 (in- ferredBy.Ort	2	FUCA1; FUCA2
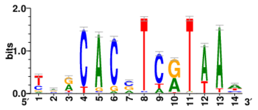	Hydrolase	taipale. tf. pairs.HOXD12. TBX21. YNRCAC- STCGT- WAA. CAP	5.55	0.2880	HOXD12; TBX21 (directAn- notation).	2	FUCA2; NEU2
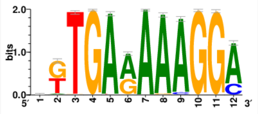	Hydrolase	transfac. pro.M06282	5.45	0.2830	ZSCAN10 (in- ferredBy.Ort	2	FUCA2; NEU4
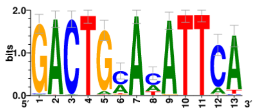	Hydrolase	metacluster. 58.2	5.39	0.2800	SOX10; SOX9; SOX9 (directAn- notation).	2	FUCA2; NEU4

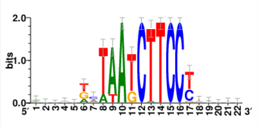	Hydrolase	tfdimers.MD	5.33	0.2770	GABPA; PITX3 (directAnnotation).	4	FUCA1; FUCA2; NEU3; NEU4
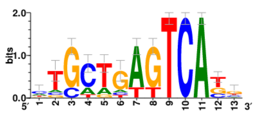	Hydrolase	metacluster. 22.38	5.32	0.2770	NFE2L2 (directAnnotation). MAFK (inferredBy.Orthology).	2	FUCA2; NEU4
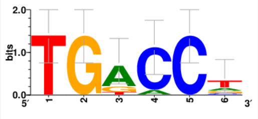	Hydrolase	transfac. pro.M03790	5.29	0.2750	NR1H4 (directAnnotation).	2	FUCA2; NEU4
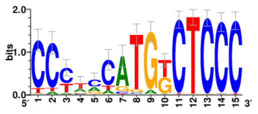	Hydrolase	metacluster. 155.33	5.22	0.2720	ZNF16; ZNF816 (directAnnotation).	2	FUCA1; NEU4
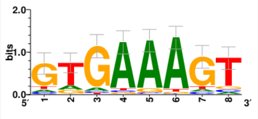	Hydrolase	kznf.PRDM1 modENCODE.1. ChIP-seq	5.20	0.2710	PRDM1 (directAnnotation).	2	NEU3; NEU4
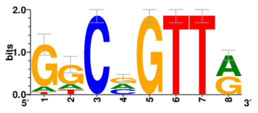	Hydrolase	homer.GGCVGTTR. MYB	5.18	0.2700	MYB (inferredBy.Orthology).	2	NEU3; NEU4

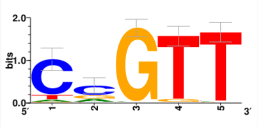	Hydrolase	metacluster. 113.4	5.15	0.2690	MYB; MYB; MYBL1; MYBL2 (directAn- notation). MYB; MYB; MYBL1; MYBL2 (in- ferredBy.Ort	2	NEU3; NEU4
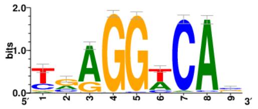	Hydrolase	hocomoco.THA MOUSE.H11MO.1.C	4.89	0.2560	THRA (in- ferredBy.Orthology).	2	FUCA2; NEU4
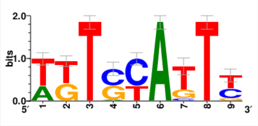	Hydrolase	kznf.ZNF620 Im- beault2017. RP. RCADE	4.77	0.2500	ZNF620 (directAn- notation).	2	FUCA2; NEU4
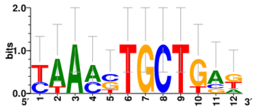	Hydrolase	transfac. pro.M07337	4.75	0.2500	BANP (directAn- notation).	2	FUCA1; NEU4
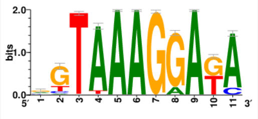	Hydrolase	transfac. pro.M05705	4.70	0.2470	ZNF239 (in- ferredBy.Ort	2	FUCA1; NEU4
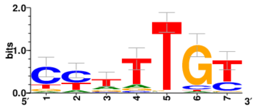	Hydrolase	metacluster. 16.1	4.68	0.2460	SOX9; ZIK1; ZIK1; ZNF782 (directAn- notation). SOX9 (in- ferredBy.Orthology).	2	NEU2; NEU4

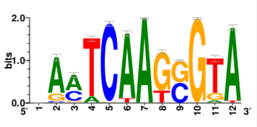	Hydrolase	transfac. pro.M06089	4.66	0.2450	ZNF26 (directAn- notation).	2	FUCA2; NEU1
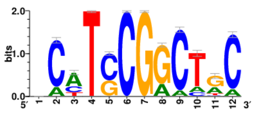	Hydrolase	transfac. pro.M06156	4.58	0.2410	ZNF75D (directAn- notation).	2	FUCA1; FUCA2
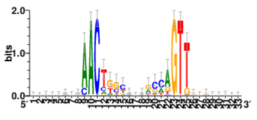	Hydrolase	tfdimers.MD	4.55	0.2400	MYB (directAn- notation).	3	NEU1; NEU3; NEU4
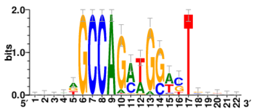	Hydrolase	tfdimers.MD00[illegible]	[illegible]	0.2390	FOXM1; TFAP2A (directAn- notation).	2	FUCA1; NEU2
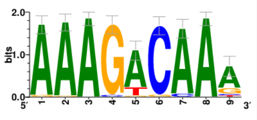	Hydrolase	kznf.ZNF197 Im- beault2017. OM. RCADE	4.48	0.2370	ZNF197 (directAn- notation).	2	FUCA2; NEU1
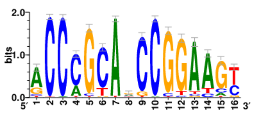	Hydrolase	taipale. tf. pairs.GCM1. ELK3. ACCCG- CANCCG- GAAGN. CAP. repr	4.43	0.2340	ELK3; GCM1 (directAn- notation).	2	FUCA1; FUCA2
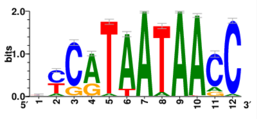	Hydrolase	transfac. pro.M06782	4.36	0.2310	ZNF225 (directAn- notation).	2	FUCA2; NEU3

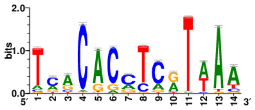	Hydrolase	taipale. tf. pairs.HOXD12. EOMES. TMRCAC-CTCRT-WAA. CAP. repr	4.24	0.2250	EOMES; HOXD12 (directAn-notation).	2	FUCA2; NEU2
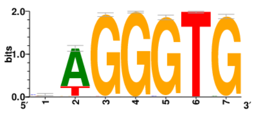	Hydrolase	transfac. pro.M06202	4.14	0.2200	ZXDB (directAn-notation).	3	FUCA2; NEU3; NEU4
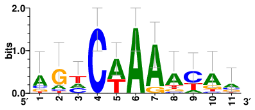	Hydrolase	transfac. pro.M08811	4.10	0.2180	FOXL2 (directAn-notation).	2	FUCA2; NEU4
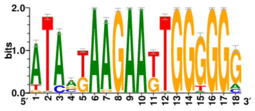	Hydrolase	transfac. pro.M06901	4.06	0.2160	ZNF484 (directAn-notation).	2	FUCA1; NEU4
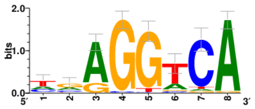	Hydrolase	homer.TRAGGTCA. THRb	4.01	0.2140	THRB (in-ferredBy.Orthology).	2	FUCA2; NEU4
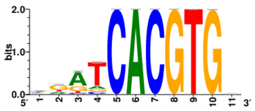	Hydrolase	hocomoco.M HU-MAN.H11M(	3.97	0.2120	MLX (directAn-notation).	2	FUCA1; NEU1
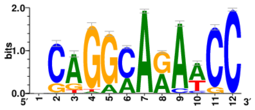	Hydrolase	transfac. pro.M06077	3.92	0.2090	ZNF716 (directAn-notation).	2	FUCA2; NEU4

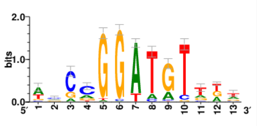	Hydrolase	taipale. tf. pairs.FOXO1 SPDEF. WMSCG-GAT-GTKNW. CAP. repr	3.84	0.2060	FOXO1; SPDEF (directAn-notation).	2	FUCA2; NEU4
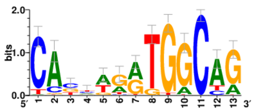	Hydrolase	kznf.CTCF. Transfac.6. Transfac	3.83	0.2050	CTCF (directAn-notation).	2	FUCA1; NEU1
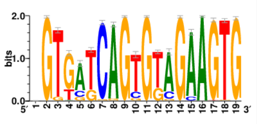	Hydrolase	transfac. pro.M05933	3.71	0.1990	ZNF552 (directAn-notation).	4	FUCA2; NEU1; NEU2; NEU3
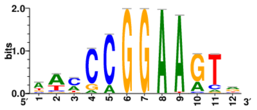	Hydrolase	jaspar.MA1483.2	3.68	0.1980	ELF2 (directAn-notation).	2	FUCA2; NEU4
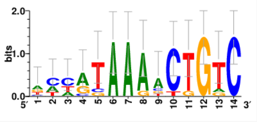	Hydrolase	taipale. tf. pairs.HOX13 MEIS1. NNNR-TAAANCT-GTN. ChIP. Exo	3.66	0.1970	MEIS1 (directAn-notation).	3	FUCA2; NEU3; NEU4
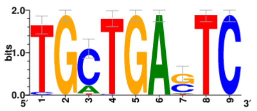	Hydrolase	transfac. pro.M04699	3.62	0.1950	MAFK (directAn-notation).	2	NEU3; NEU4
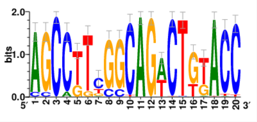	Hydrolase	metacluster. 172.14	3.60	0.1940	ZFX; ZFY (directAn-notation).	2	FUCA2; NEU4

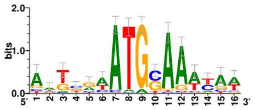	Hydrolase	hocomoco.PO[illegible] HU-MAN.H11MO.0.C	3.60	0.1940	POU2F1 (directAn-notation).	2	FUCA1; NEU4
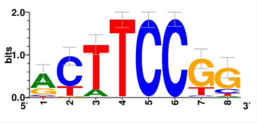	Hydrolase	metacluster. 166.2	3.57	0.1920	ELK4; ETS1; ETS1; ETV1; ETV3; ETV4; ETV5; ETV7 (directAn-notation). GABPA (in-ferredBy.Ort	3	FUCA1; FUCA2; NEU4
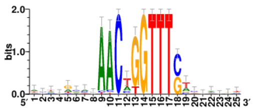	Hydrolase	tfdimers.MD00[illegible]	[illegible]	0.1910	MYB; ZEB1 (directAn-notation).	2	NEU3; NEU4
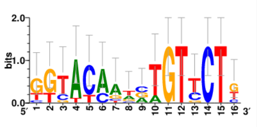	Hydrolase	transfac. pub-lic.M00205	3.51	0.1900	NR3C1 (directAn-notation).	2	NEU2; NEU4
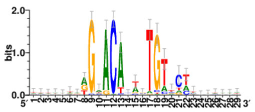	Hydrolase	tfdimers.MD00[illegible]	[illegible]	0.1870	NR3C1 (directAn-notation).	4	FUCA1; FUCA2; NEU3; NEU4
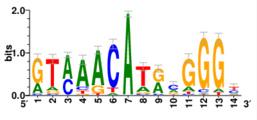	Hydrolase	taipale. tf. pairs.GCM1. FOXI1. RTMAA-CA-TRNGGGN. CAP. repr	3.42	0.1850	FOXI1; GCM1 (directAn-notation).	2	FUCA1; FUCA2

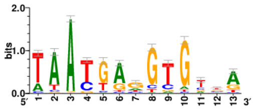	Hydrolase	taipale. tf. pairs.HOXA3. EOMES. TAATK-AGGT-GNKA. CAP. repr	3.42	0.1850	EOMES; HOXA3 (directAn-notation).	3	FUCA2; NEU2; NEU4
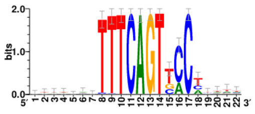	Hydrolase	tfdimers.MD	3.39	0.1840	E2F1; LTF (directAn-notation).	2	NEU3; NEU4
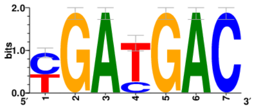	Hydrolase	kznf.ZNF669. Na-jafabadi2015. ChIP-seq	3.38	0.1830	ZNF669 (directAn-notation).	2	NEU2; NEU4
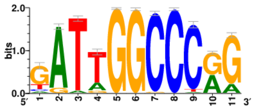	Hydrolase	transfac. pro.M05403	3.31	0.1800	ADNP2 (in-ferredBy.Ort	5	FUCA1; FUCA2; NEU2; NEU3; NEU4
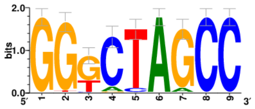	Hydrolase	kznf.ZKSCAN3. Im-beault2017. OM. RCADE	3.29	0.1790	ZKSCAN3 (directAn-notation).	2	NEU2; NEU3
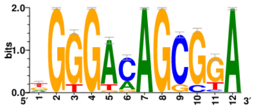	Hydrolase	transfac. pro.M06226	3.26	0.1780	ZNF460 (directAn-notation).	2	NEU1; NEU4
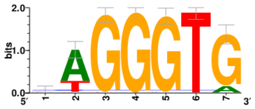	Hydrolase	metacluster. 66.10	3.25	0.1770	ZXDA (directAn-notation).	3	FUCA2; NEU3; NEU4

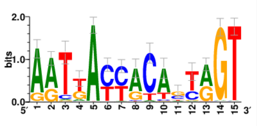	Hydrolase	metacluster. 112.1	3.24	0.1770	ZNF433 (directAn- notation).	4	FUCA1; FUCA2; NEU3; NEU4
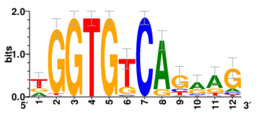	Hydrolase	metacluster. 158.2	3.16	0.1730	ZNF789; ZNF789 (directAn- notation).	2	FUCA2; NEU3
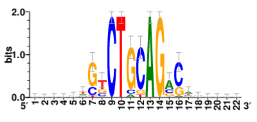	Hydrolase	tfdimers.MD	3.15	0.1730	SMAD4 (directAn- notation).	2	FUCA2; NEU4
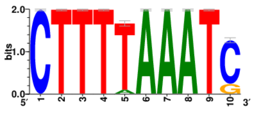	Hydrolase	transfac. pro.M05390	3.11	0.1710	TBPL2 (directAn- notation).	3	FUCA1; FUCA2; NEU1
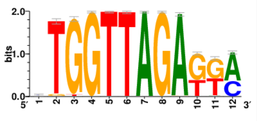	Hydrolase	transfac. pro.M06366	3.09	0.1690	ZNF530 (directAn- notation).	2	FUCA1; NEU2
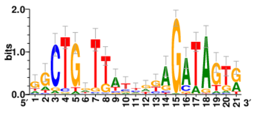	Hydrolase	kznf.ZNF792. Im- beault2017. RP. ChIP-seq	3.06	0.1680	ZNF792 (directAn- notation).	2	NEU3; NEU4
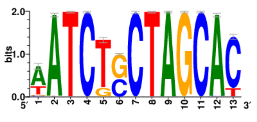	Hydrolase	transfac. pro.M05449	3.04	0.1670	ZNF414 (in- ferredBy.Ort	2	FUCA2; NEU1

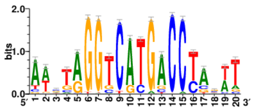	Hydrolase	taipale. tf. pairs.RORB. AWNTAG- GTCAT- GACC- TANWT. HT	3.04	0.1670	RORB (directAn- notation).	3	FUCA1; NEU2; NEU3
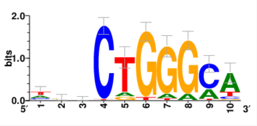	Hydrolase	homer.WDN ZNF416	3.03	0.1670	ZNF416 (directAn- notation).	1	NEU4
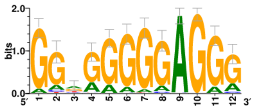	Hydrolase	kznf.ZNF467. Schmit- ges2016. RCADE	3.03	0.1670	ZNF467 (directAn- notation).	1	NEU4
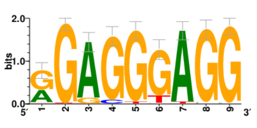	Hydrolase	swissregulon.	3.03	0.1670	GTF2I (directAn- notation).	1	NEU4
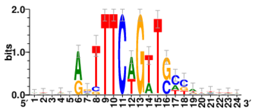	Hydrolase	tfdimers.MD00351	3.03	0.1670	IRF8; RFX1; RFX2; RFX3; RFX4; RFX5; RFXANK; RFXAP (directAn- notation).	1	NEU4
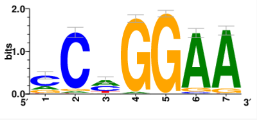	Hydrolase	transfac. pro.M01776	3.03	0.1670	NR2C2 (directAn- notation).	1	NEU4

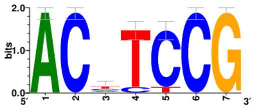	Hydrolase	transfac. pro.M04826	3.03	0.1670	EP300 (directAn- notation).	1	NEU4
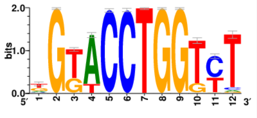	Hydrolase	transfac. pro.M06593	3.03	0.1670	ZNF799 (directAn- notation).	1	NEU4
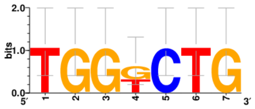	Hydrolase	hdpi.SMAD3	3.03	0.1670	SMAD3 (directAn- notation).	1	NEU4
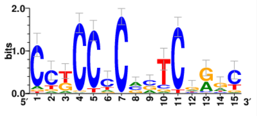	Hydrolase	metacluster. 133.1	3.03	0.1670	ZNF304; ZNF304; ZSCAN22 (directAn- notation).	1	NEU4
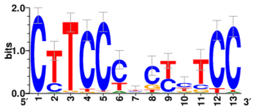	Hydrolase	metacluster. 35.8	3.03	0.1670	ZNF132; ZNF132 (directAn- notation).	1	NEU4
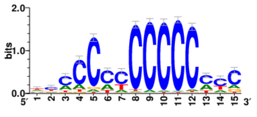	Hydrolase	swissregulon.	3.03	0.1670	ZNF281 (directAn- notation).	1	NEU4
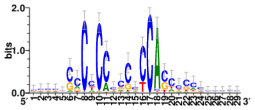	Hydrolase	tfdimers.MD00403	3.03	0.1670	SREBF2; WT1 (directAn- notation).	1	NEU4

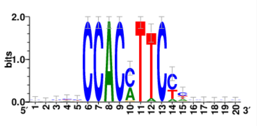	Hydrolase	tfdimers.MD[illegible]	3.03	0.1670	STAT6; ZNF354C (directAnnotation).	1	NEU4
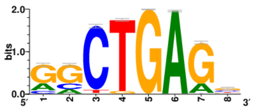	Hydrolase	swissregulon.hs.PAX5	3.02	0.1660	PAX5 (directAnnotation).	1	NEU4
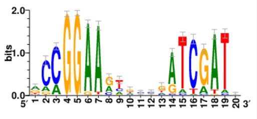	Hydrolase	taipale. tf. pairs.ERF. ONE-CUT2. RSCG-GAANNNNN[illegible] GATN. CAP	3.02	0.1660	ERF; ONECUT2 (directAnnotation).	1	NEU4
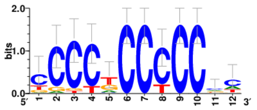	Hydrolase	transfac. pro.M07397	3.02	0.1660	ZNF148 (directAnnotation).	1	NEU4
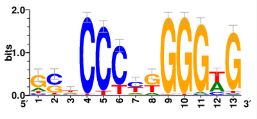	Hydrolase	dbtfbs.ZNF4[illegible] HEK293. ENCSR477O[illegible] merged. N1	3.02	0.1660	ZNF423 (directAnnotation).	1	NEU4
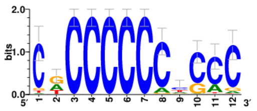	Hydrolase	transfac. pro.M01122	3.02	0.1660	ZNF219 (directAnnotation).	1	NEU4
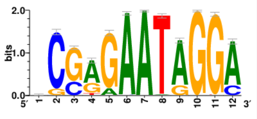	Hydrolase	transfac. pro.M06137	3.02	0.1660	ZNF250 (directAnnotation).	1	NEU4

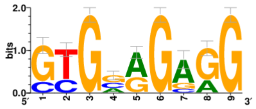	Hydrolase	kznf.ZNF267. Imbeault2017. OM. RCADE	3.02	0.1660	ZNF267 (directAnnotation).	1	NEU4
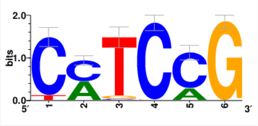	Hydrolase	metacluster. 135.8	3.02	0.1660	ZNF516; ZNF536 (inferredBy.Ort	1	NEU4
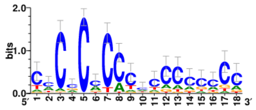	Hydrolase	metacluster. 3.2	3.02	0.1660	KLF8; TBX15 (directAnnotation).	1	NEU4
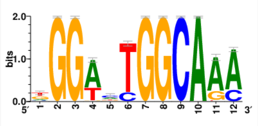	Hydrolase	transfac. pro.M06149	3.02	0.1660	ZNF132 (directAnnotation).	2	FUCA2; NEU2
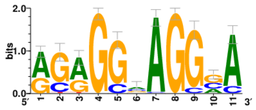	Hydrolase	kznf.ZNF540. Imbeault2017. RP. ChIP-seq	3.02	0.1660	ZNF540 (directAnnotation).	1	NEU4
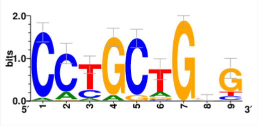	Hydrolase	metacluster. 80.9	3.02	0.1660	ZIC2 (directAnnotation).	1	NEU4
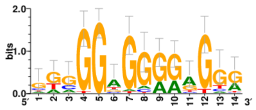	Hydrolase	transfac. pro.M02023	3.02	0.1660	MAZ (directAnnotation).	1	NEU4

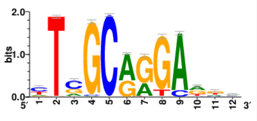	Hydrolase	hocomoco.KA HU-MAN.H11MO	3.01	0.1660	ZBTB33 (directAn-notation).	1	NEU4
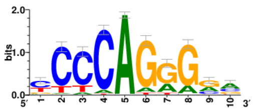	Hydrolase	hocomoco.PRD16 MOUSE.H11MO.0.B	3.01	0.1660	PRDM16 (in-ferredBy.Orthology).	1	NEU4
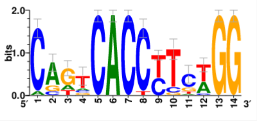	Hydrolase	metacluster. 198.1	3.01	0.1660	ZNF439 (directAn-notation).	3	FUCA2; NEU3; NEU4
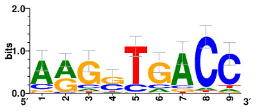	Hydrolase	transfac. pro.M04881	3.01	0.1660	ESR1 (directAn-notation).	1	NEU4
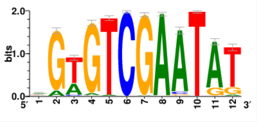	Hydrolase	transfac. pro.M06580	3.01	0.1660	ZNF560 (directAn-notation).	1	FUCA2
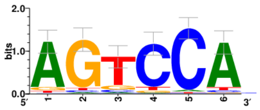	Hydrolase	cisbp.M08220	3.01	0.1660	HNF4A (directAn-notation).	1	NEU4
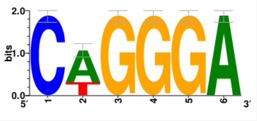	Hydrolase	kznf.ZNF557 Im-beault2017. OM. MEME	3.01	0.1660	ZNF557 (directAn-notation).	1	NEU4
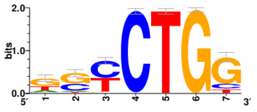	Hydrolase	hocomoco.HAND1. HU-MAN.H11MO.1.D	3.01	0.1660	HAND1 (directAn-notation).	1	NEU4

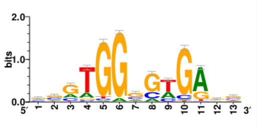	Hydrolase	hocomoco.SF HU- MAN.H11M(	3.01	0.1660	SREBF2 (directAn- notation).	1	NEU4
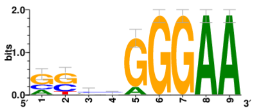	Hydrolase	kznf.IKZF3. Schmit- ges2016. RCADE	3.01	0.1660	IKZF3 (directAn- notation).	1	NEU4
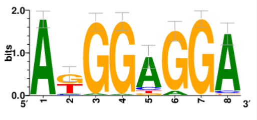	Hydrolase	metacluster. 24.11	3.01	0.1660	ZSCAN22; ZSCAN22 (directAn- notation).	1	NEU4
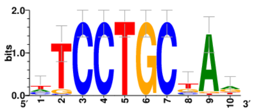	Hydrolase	transfac. pro.M01119	3.01	0.1660	ZBTB33 (directAn- notation).	1	NEU4
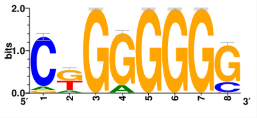	Hydrolase	transfac. pro.M06457	3.01	0.1660	ZNF451 (in- ferredBy.Ort	1	NEU4
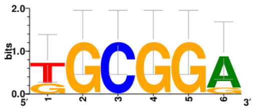	Hydrolase	hdpi.HIP2	3.00	0.1650	UBE2K (directAn- notation).	1	NEU4
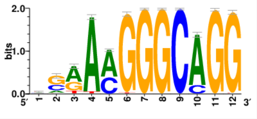	Hydrolase	transfac. pro.M05742	3.00	0.1650	ZNF57 (directAn- notation).	1	NEU4
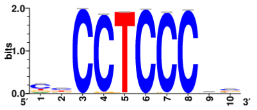	Hydrolase	jaspar.MA1965[illegible]	3.00	0.1650	SP5 (directAn- notation).	1	NEU4

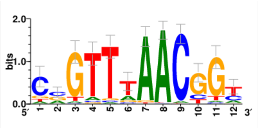	Hydrolase	metacluster. 113.1	3.00	0.1650	MYBL1; MYBL2 (directAn- notation).	1	NEU4
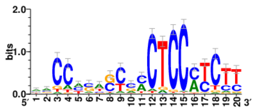	Hydrolase	transfac. pro.M12604	3.00	0.1650	ZNF444 (directAn- notation).	1	NEU4

geneSet: The glycosylation pathway to which the glycosyltransferes belong.

Motif: ID of the motif.

NES: Normalized enrichment score of the motif in the gene-set. It was calculated based on the AUC distribution of all the motifs for the gene-set [(x-mean)/sd].

AUC: area under the curve.

TF.highConf: Transcription factors with high confident.

nEnrGenes: The number of genes that are highly ranked for the given motif.

enrichedGenes: hgnc_symbol of genes that are highly ranked for the given motif.
